# Spatiotemporally programmed hybrid nano-prodrugs orchestrate multidimensionally synergistic cascade lethality in NSCLC

**DOI:** 10.7150/thno.135204

**Published:** 2026-08-24

**Authors:** Yan Wang, Chaozheng Zhang, Chen Sun, Yao Chen, Jielu Zhang, Yongsen He, Xue Sun, Jingping Wu, Xiaosong Xu, Xingtong Li, Li Deng, Fang Yan, Lang He, Tianbao Wang, Xin Zhou, Tingting Zhang, Maolin Wang, Jin Liu, Zhizhong Wang, Shilin Chen, Bo Ren, Jun Lu

**Affiliations:** 1 Chinese Medicine Germplasm Resources Innovation and Effective Uses Key Laboratory of Sichuan Province, Institute of Herbgenomics, School of Pharmacy, Chengdu University of Traditional Chinese Medicine, Chengdu 611137, China.; 2 School of Pharmacy, Ningxia Medical University, Yinchuan, Ningxia 750004, China.; 3 Department of Medical Cosmetology, Hospital of Chengdu University of Traditional Chinese Medicine, Chengdu 610075, China.; 4 The Second Clinical Medical College, Affiliated Fifth People’s Hospital of Chengdu University of Traditional Chinese Medicine, Chengdu 611130 Sichuan, China.; 5 Sichuan Clinical Research Center for Cancer, Sichuan Cancer Hospital & Institute, Sichuan Cancer Center, Affiliated Cancer Hospital of University of Electronic Science and Technology of China, Chengdu 610041, China.; 6 Department of Integrative Medical Biology (IMB), Faculty of Medicine, Umeå University, 90187 Umeå, Sweden.; 7 The First Affiliated Hospital of Shantou University Medical College, Shantou 515041, China.; 8 Institute for Advancing Translational Medicine in Bone & Joint Diseases, School of Chinese Medicine, Hong Kong Baptist University, Hong Kong 999077, China.

**Keywords:** membrane lipid peroxidation stress, GSH/GPX4 axis, DNA damage stress, non-small cell lung cancer, etoposide

## Abstract

**Rationale:**

Non-small cell lung cancer (NSCLC) develops a high GSH/GPX4 antioxidant phenotype under persistent oxidative pressure, which suppresses membrane lipid peroxidation and ferroptosis, constituting a core mechanism underlying chemotherapy resistance and suboptimal therapeutic efficacy. Breaking this resistance barrier demands not simply attacking the tumor but disarming the antioxidant defense to intensify oxidative damage and awaken durable antitumor immunity.

**Methods:**

A self-assembled nano-prodrug, E-R@ISSL, was engineered by co-assembling a carbamate-linked RGD-modified etoposide (ETP) prodrug and a disulfide-bonded indole derivative conjugated to linoleic acid (LA) at an optimal 1:2 molar ratio. The system thus possesses dual αvβ5/mitochondria targeting with CES2/GSH cascade-responsive drug release. Antitumor activity and mechanisms were evaluated *in vitro* and in both subcutaneous and orthotopic NSCLC mouse models.

**Results:**

E-R@ISSL exhibited efficient αvβ5-mediated internalization and mitochondria-targeted delivery. Upon sequential CES2/GSH-triggered disassembly, E-R@ISSL co-released ETP and LA, reduced the available intracellular GSH pool, and functionally dampened the GSH-dependent GPX4 antioxidant defense. ETP induced DNA double-strand breaks and elevated reactive oxygen species (ROS), while LA expanded the oxidizable lipid pool, synergistically driving lipid peroxidation and ferroptosis. Concurrently, the system activated immunogenic cell death (ICD), promoting dendritic cell maturation and enhancing CD8^+^ T cell infiltration. In orthotopic NSCLC models, E-R@ISSL significantly suppressed tumor progression, prolonged survival, and demonstrated a favorable safety profile with reduced systemic toxicity compared with free ETP.

**Conclusions:**

Together, these findings demonstrate that E-R@ISSL achieved coordinated DNA damage, ferroptosis, and ICD induction through its dual-targeted and sequentially activated co-delivery of ETP and LA, concurrently dismantling the GSH/GPX4 antioxidant defense and intensifying oxidative injury. This nano-prodrug thus represents a viable strategy for overcoming treatment resistance and extending durable antitumor responses in NSCLC.

## Introduction

Non-small cell lung cancer (NSCLC) arises in the oxygen-rich lung, while the abnormal vasculature within the tumor creates microscopic hypoxic zones. This coexistence of macro-scale oxygenation and micro-scale hypoxia jointly leads to its particularly pronounced membrane lipid peroxidation stress 1, 2. To survive under this oxidative burden, tumor cells mobilize a glutathione (GSH)-orchestrated antioxidant defense that maintains redox equilibrium and membrane integrity, effectively suppressing unchecked lipid peroxidation 3, 4. Beyond its role as a principal intracellular reductant buffer, GSH provides essential reducing equivalents to glutathione peroxidase 4 (GPX4), which catalyzes the conversion of polyunsaturated phospholipid hydroperoxides to inert alcohols, a step that halts the lipid peroxidation chain and blocks ferroptosis 5-7. Consequently, NSCLC cells frequently adopt a GSH-rich, GPX4-high antioxidant phenotype, buffering lipid peroxidation-induced lethal damage while simultaneously promoting resistance to diverse therapeutic modalities.

Linoleic acid (LA) can be activated and incorporated into membrane phospholipids via lipid remodeling, significantly expanding the intracellular supply of oxidizable lipid substrates 8-10. Under oxidative stress, membrane phospholipids bearing polyunsaturated fatty acids (PUFA) are more susceptible to radical-mediated chain autoxidation, a process further amplified by lipoxygenase catalysis, driving progressive generation and accumulation of lipid peroxides (LPOs) 11, 12. When the GSH/GPX4 axis is functionally impaired or overwhelmed, uncontrolled LPOs accumulation disrupts membrane integrity and triggers ferroptosis 13-16. Accordingly, flooding tumor cells with exogenous PUFA such as LA while simultaneously compromising their antioxidant defenses represents a rational strategy to weaponize lipid peroxidation susceptibility for tumor intervention 17-19. Furthermore, sustained LPOs accumulation exacerbates membrane lipid oxidative damage and disrupts cellular redox homeostasis, thereby compromising the cellular capacity to buffer and repair DNA damage stress. This provides a synergistic foundation for DNA-damaging agents to induce more potent cell death responses. Etoposide (ETP), a classic topoisomerase II inhibitor, traps the transient drug–enzyme–DNA ternary complex, leading to DNA double-strand breaks that trigger apoptosis 20-22. Concurrently, ETP significantly elevates intracellular reactive oxygen species (ROS) levels, which heightens membrane lipid oxidative stress and may act synergistically with LA-mediated LPO buildup to drive PUFA peroxidation and cell death 23. However, ETP lacks tumor-selective distribution *in vivo* and exhibits considerable systemic toxicity. DNA damage stress may also prompt tumor cells to reinforce their reductive capacity and activate stress adaptation programs, muting apoptotic signals and progressively undermining treatment response while driving resistance 24, 25. Consequently, achieving precise drug delivery and controlled intracellular release is crucial for reducing ETP nonspecific toxicity and enhancing its intratumoral therapeutic effect.

The RGD peptide specifically recognizes multiple integrin receptors overexpressed on tumor cells. Notably, integrin αvβ5, significantly upregulated in NSCLC, provides an ideal portal for RGD-directed endocytosis, substantially improving the tumor-targeting specificity and intracellular delivery 26-28. Carbamate bonds offer favorable systemic stability yet undergo selective hydrolysis in the presence of carboxylesterase (CES), enabling site-specific drug liberation 29, 30. NSCLC cells often display appreciable CES2 expression and corresponding hydrolytic activity, justifying CES-activated prodrug design for intracellular-selective drug release 31-33. In parallel, disulfide bonds can exploit the high GSH environment of NSCLC cells, undergoing thiol-disulfide exchange to trigger drug liberation 34, 35. This process may reduce the intracellular GSH reserve and disturb redox homeostasis, thereby functionally weakening the GSH-dependent GPX4 antioxidant defense rather than directly inhibiting GPX4 itself. In recent years, indocyanine derivatives have emerged as important vehicles for mitochondrial-targeted delivery owing to their unique photophysical properties, biocompatibility, and facile chemical conjugation 36, 37. One prominent example, indole-aza-anthocyanin (IAA), carries a lipophilic cationic center that drives mitochondrial accumulation in response to the mitochondrial membrane potential (MMP). Its hydrophobic π-conjugated backbone further promotes self-assembly through π-π stacking and hydrophobic interactions, yielding structurally stable nanosystems with intrinsic fluorescence suitable for imaging and tracking.

**Scheme 1:**
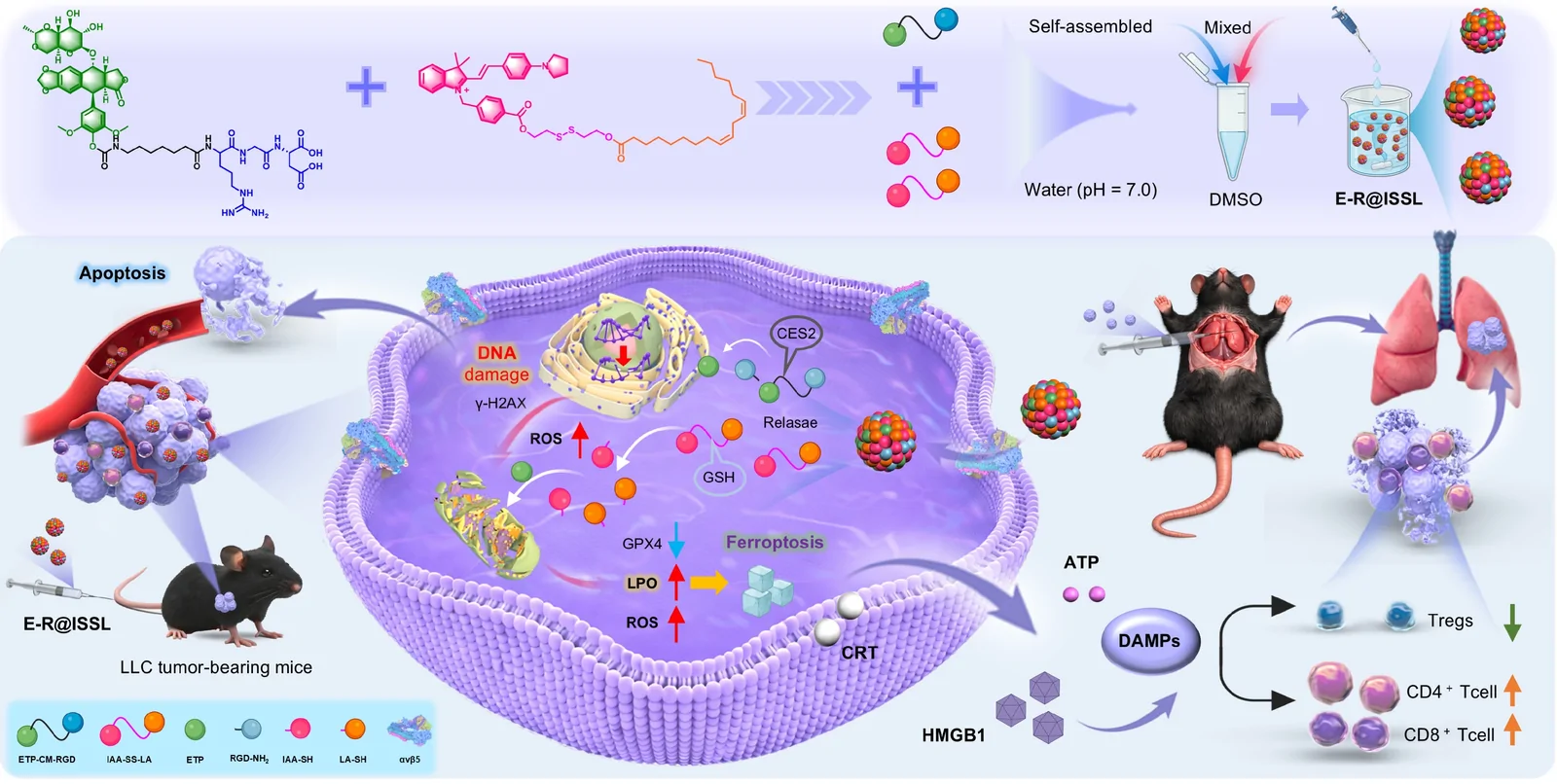
** Schematic illustration of the E-R@ISSL nano-prodrug for NSCLC therapy.** The self-assembled system integrates dual-targeting (RGD/αvβ5 and mitochondria) and cascade-responsive (CES2/GSH) release to co-deliver ETP and LA. This strategy simultaneously induces DNA damage, ferroptosis, and mitochondrial apoptosis, which collectively trigger immunogenic cell death and subsequent antitumor immune activation.

This study designed and constructed a self-assembled nano-prodrug, E-R@ISSL, possessing dual-targeting capabilities toward tumor cells and mitochondria, capable of cascade-responsive release following tumor tissue accumulation (**Scheme 1**). Through the “dual-targeting-cascade-responsive” nano-prodrug, tumor-specific enrichment and controlled release of chemotherapeutic agents were achieved, significantly reducing nonspecific tissue exposure and enhancing effective intratumoral drug load. Moreover, via a multi-mechanistic synergistic strategy of “DNA damage-ferroptosis-immune activation”, the nano-prodrug not only enhances the triggering efficiency of tumor cell death but also establishes an intrinsic link between cytotoxic therapy and immune effect amplification, laying a scientific foundation for improving the durability of antitumor efficacy.

## Materials and Methods

Materials, synthesis and preparation of hybrid nano-prodrugs, detailed characterization procedures, as well as* in vitro*, *in vivo* assays are provided in the **Supplementary Information**.

## Results and Discussion

### αvβ5 overexpression and glutathione metabolism in NSCLC

**Figure 1:**
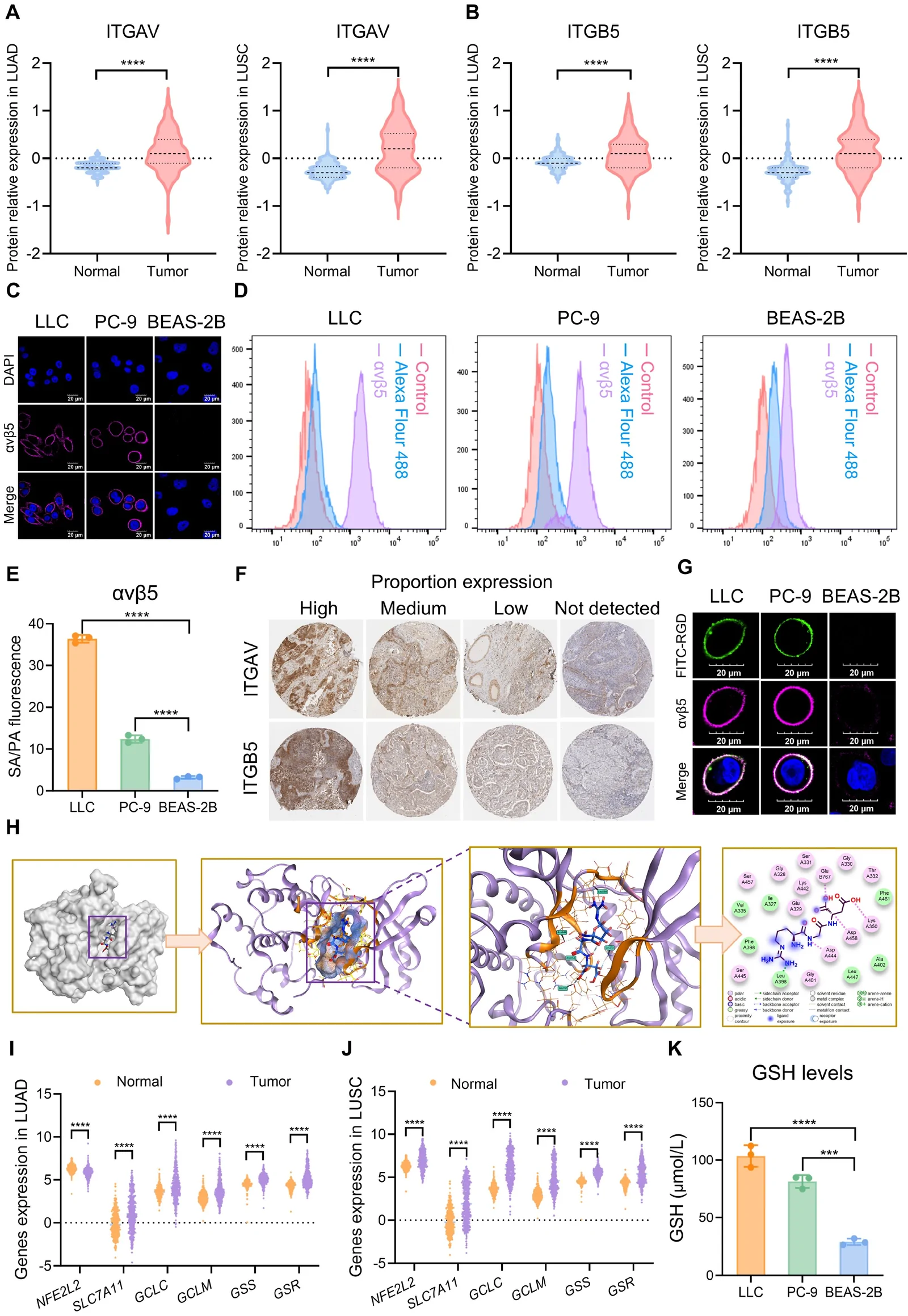
** Potential effects of αvβ5 overexpression and high GSH on NSCLC.** (**A**, **B**) Comparative analysis of ITGAV (A) and ITGB5 (B) relative expression levels in LUAD, LUSC, and normal tissue. (**C**) Detection of αvβ5 expression levels by immunofluorescence staining. (**D**, **E**) Detection and analysis of αvβ5 expression levels by flow cytometry. (**F**) Expression levels of ITGAV and ITGB5 in lung cancer immunohistochemistry. (**G**) Co-localization of RGD and αvβ5 by immunofluorescence staining. (**H**) Molecular docking analysis of RGD with αvβ5. (**I**, **J**) GSH-related gene expressions between tumor and normal tissues in LUAD (I) and LUSC (J). (**K**) Comparison of GSH concentrations in PC-9, LLC and BEAS-2B cells. ns, no substantial change, ***p < 0.001, ****p < 0.0001.

The αvβ5, a typical RGD sequence-recognizing receptor, is overexpressed in numerous solid tumors. According to CPTAC (Clinical Proteomic Tumor Analysis Consortium) proteomics data analysis, both αvβ5 subunits, ITGAV and ITGB5, were significantly elevated in lung adenocarcinoma (LUAD) and lung squamous cell carcinoma (LUSC) tissues compared with normal lung tissues, suggesting a distinct upregulation of αvβ5 in NSCLC (**Figure 1A-B**). Cellular validation confirmed that membrane αvβ5 was expressed at markedly higher levels in LLC and PC-9 cells relative to normal BEAS-2B bronchial epithelial cells, consistent with tumor-selective expression (**Figure 1C-E**). Corroborating this, tissue microarray immunohistochemistry from the Human Protein Atlas (HPA) gave ITGAV and ITGB5 positive rates of 83.3% and 72.7% in NSCLC specimens (**Figure 1F**). FITC-labeled RGD was next used to probe RGD–αvβ5 binding by immunofluorescence co-localization. Analysis revealed that RGD fluorescence strongly overlapped with αvβ5 in LLC and PC-9 cells. Conversely, in αvβ5-low BEAS-2B cells, only weak RGD signals with minimal co-localization were detected (**Figure 1G**). Molecular docking simulations further indicated that the binding pocket formed by ITGAV and ITGB5 engages the RGD peptide through a network of hydrogen bonds (ΔG = –7.22 kcal/mol), pointing to high binding affinity (**Figure 1H**). Furthermore, analysis of the TCGA and GTEx databases revealed significant upregulation of key genes involved in GSH synthesis and regeneration pathways (*GCLC, GCLM, GSS, GSR*) in both LUAD and LUSC (**Figure 1I-J**). Consistent with these findings, cellular assays confirmed intracellular GSH levels were substantially higher in LLC and PC-9 cells than in BEAS-2B cells (**Figure 1K**). These observations collectively indicate active GSH metabolism and a reducing redox homeostasis in NSCLC, thereby providing a potential mechanistic basis for the selective action of redox-responsive therapeutic agents.

### Design and construction of the dual stimulus-responsive prodrugs

**Figure 2:**
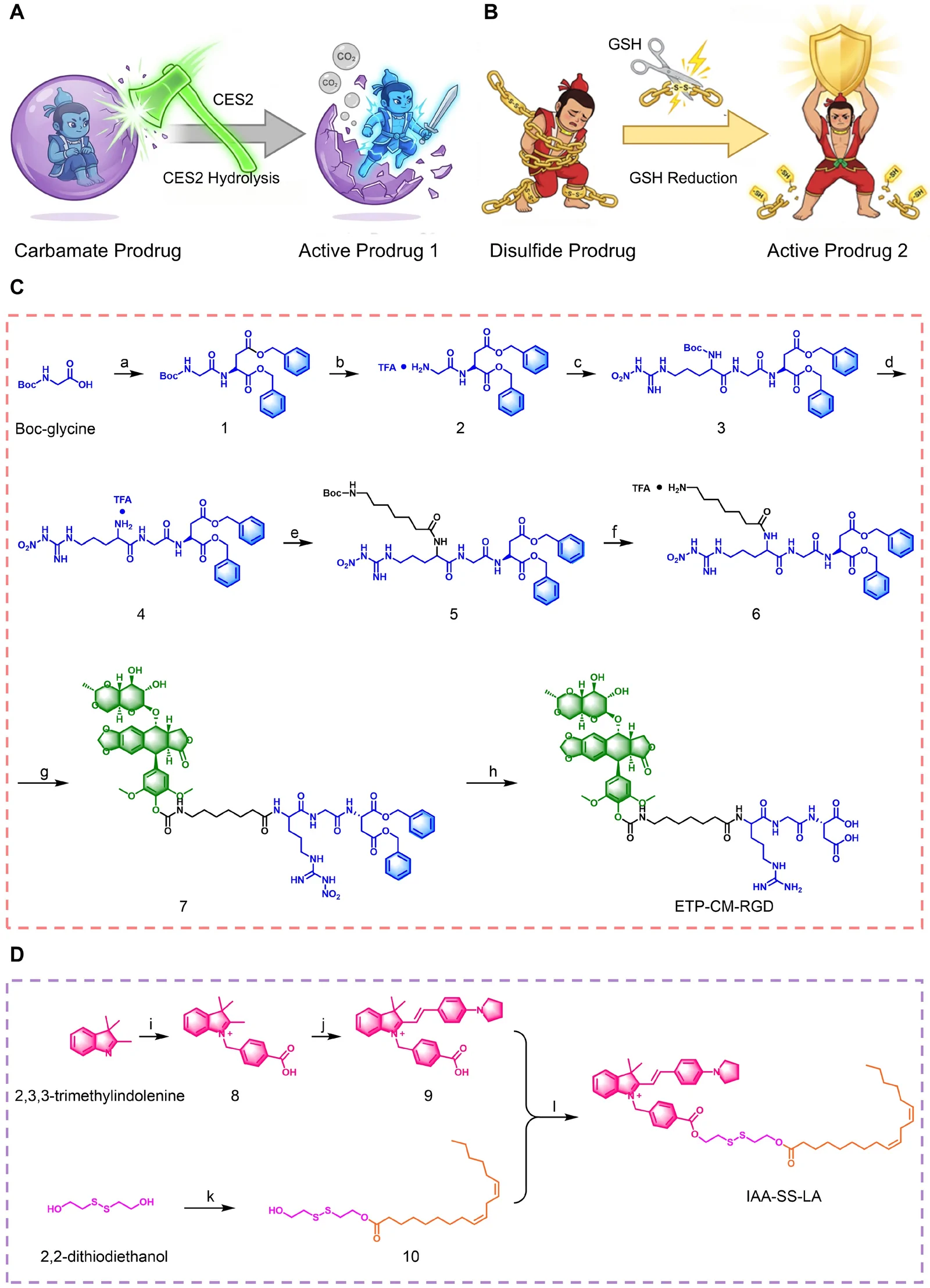
** Schematic illustration of the preparation procedures for ETP-CM-RGD and IAA-SS-LA.** (**A**) Mechanism of carbamate prodrug activation. (**B**) Mechanism of disulfide prodrug activation. (**C**) Synthesis of ETP-CM-RGD. Reagents and conditions: (a) L-Aspartic acid dibenzyl ester 4-toluenesulfonate, EDCI, HOBt, NMM, DIPEA, DCM, rt, 8 h, 90%; (b) Trifluoroacetic acid, DCM, rt, 1 h, 96%; (c) N-Boc-N'-nitro-L-arginine, EDCI, HOBt, DIPEA, rt, 12 h, 60%; (d) Trifluoroacetic acid, DCM, rt, 1 h, 90%; (e) 7-(boc-amino)heptanoic acid, DIPEA, HATU, rt, 12 h, 70%; (f) Trifluoroacetic acid, DCM, rt, 1 h, 90%; (g) ETP-PNP, Triethylamine, DMF, rt, 4 h, 60%; (h) Pd/C, H_2_, MeOH, 12 h, 60%. (D) Synthesis of IAA-SS-LA. Reagents and conditions: (i) 4-(Bromomethyl) benzoic acid, acetonitrile, 80 ℃, 8 h, 60%; (j) 4-Pyrrolidin-1-ylbenzaldehyde, piperidine, 80 ℃, 8 h, 50%; (k) Linoleic acid, EDCI, DMAP, rt, 4 h, 80%; (l) EDCI, DMAP, rt, 8 h, 50%.

Building on the previously identified NSCLC characteristics of αvβ5 overexpression and a highly reductive microenvironment, this study constructed a targeted prodrug system integrating dual molecular-recognition and dual stimulus-response mechanisms for precise synergistic therapy. This is not a simple combination of ETP and LA, but a pathology-oriented strategy to address two key barriers in NSCLC: insufficient tumor-selective delivery and systemic toxicity of free ETP, and the GSH/GPX4 antioxidant defense that buffers lipid peroxidation stress. To achieve selective intracellular activation in tumor cells, two microenvironment-responsive linkages were incorporated into the system: an enzyme-cleavable carbamate bond that remains relatively stable during systemic circulation but undergoes hydrolysis in response to tumor-overexpressed CES2, and a redox-responsive disulfide bond that can be rapidly cleaved by high intracellular GSH (**Figure 2A-B**).

Accordingly, two functional prodrug modules were synthesized. The first, ETP-CM-RGD, employed ETP as the therapeutic core with its 4′-hydroxyl group derivatized via a carbamate bond and conjugated to an RGD peptide. This design transiently masked ETP activity to reduce nonspecific exposure during circulation without permanently damaging the parent scaffold, conferred αvβ5-mediated tumor recognition and endocytosis, and enabled CES2-triggered release of active ETP within tumor cells. The synthetic route involved multiple protection/deprotection steps, followed by nucleophilic substitution and reduction to afford the target product (**Figure 2C**, and **Scheme S1**). The second module, IAA-SS-LA, incorporated an indole derivative (IAA) as a mitochondria-targeting unit linked to LA through a disulfide bond (**Figure 2D**). Under high intracellular GSH, the disulfide bond was cleaved to release LA, a PUFA substrate that expands the oxidizable lipid pool, while the concurrent thiol-disulfide exchange consumed GSH and weakened the GSH-dependent GPX4 antioxidant defense. Together, these two modules established a mechanistically coordinated platform, with ETP-CM-RGD providing tumor-recognition and intracellularly activatable DNA-damage induction, and IAA-SS-LA contributing redox-responsive mitochondrial lipid-peroxidation amplification, thereby establishing the molecular basis for the subsequent construction of the co-assembled nano-prodrug system.

### Self-assembly, physicochemical characterization, and tumor microenvironment-responsive properties of E-R@ISSL

**Figure 3:**
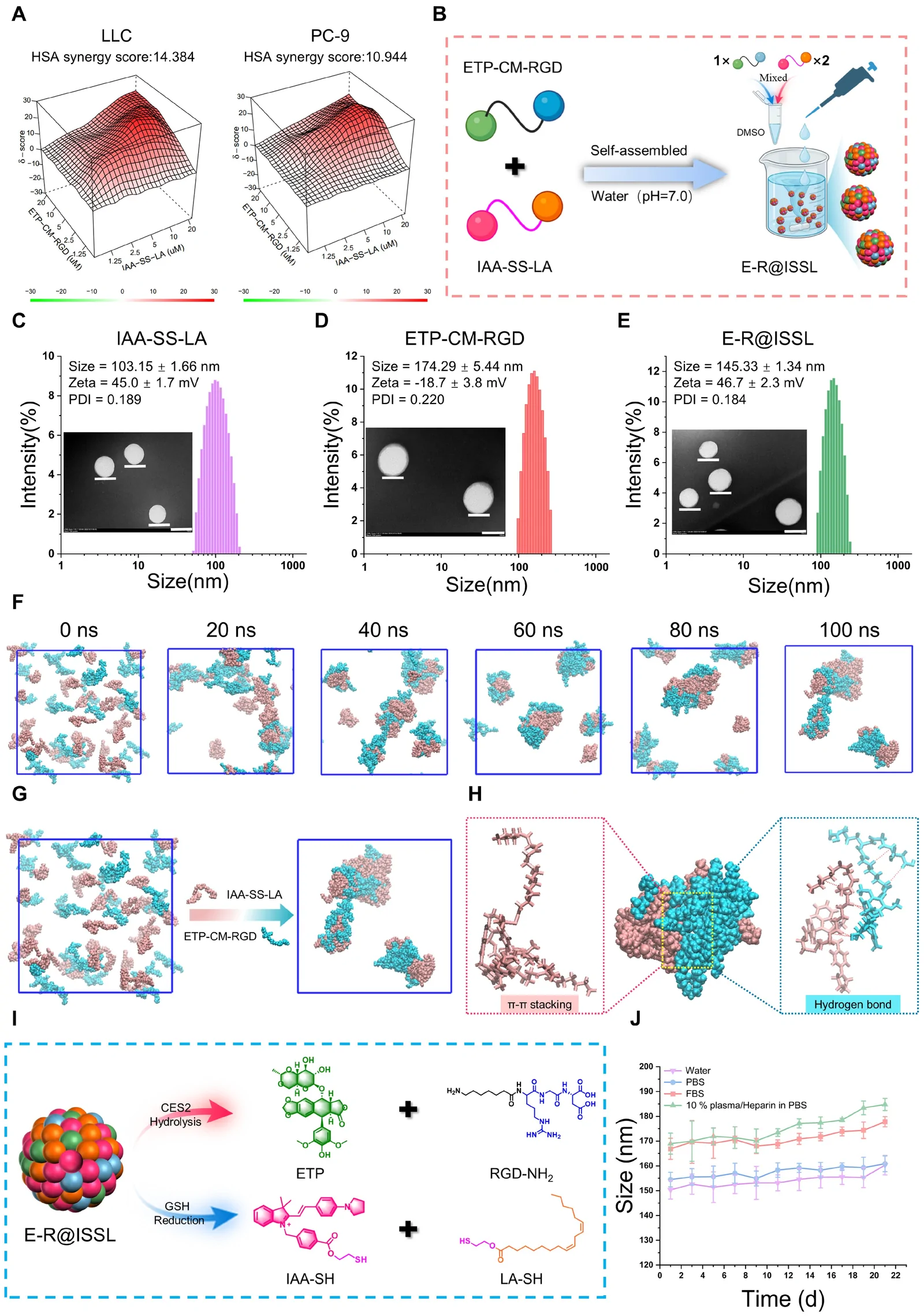
** Construction and characterization of E-R@ISSL.** (**A**) Visual inhibition rate of ETP-CM-RGD synergistic action with IAA-SS-LA in PC-9 and LLC cells. (**B**) Schematic diagram of E-R@ISSL preparation via nanoprecipitation. (**C**-**E**) Particle size distribution and TEM imaging of IAA-SS-LA NPs (C), ETP-CM-RGD NPs (D), and E-R@ISSL (E). Scale bar = 100 nm. (**F**) Time-course changes in structural variations of the E-R@ISSL per 20 ns during the simulation (red molecular clusters: IAA-SS-LA; blue molecular clusters: ETP-CM-RGD). (**G**) Structural changes of the E-R@ISSL from 0 to 100 ns. (**H**) Intermolecular interactions analysis in the E-R@ISSL. (**I**) CES2/GSH-responsive mechanism of E-R@ISSL. (**J**) Size stability of E-R@ISSL in DI water, PBS, 10% plasma/Heparin in PBS, and FBS within 21 d.

To determine the optimal co-assembly ratio, we first performed synergy analysis of ETP-CM-RGD and IAA-SS-LA using the Highest Single Agent (HSA) model. Co-treatment at a 1:2 molar ratio yielded HSA synergy scores of 10.944 and 14.384 in PC-9 and LLC cells, respectively, indicating pronounced synergistic inhibition at this ratio (**Figure 3A**). Accordingly, ETP-CM-RGD and IAA-SS-LA were co-assembled at a 1:2 molar ratio via nanoprecipitation to construct the combined nano-prodrug system, designated E-R@ISSL (**Figure 3B**). Each individual prodrug also proved capable of self-assembly under the same conditions. Dynamic light scattering (DLS) analysis measured a hydrodynamic diameter (145.33 ± 1.34 nm), a zeta potential (+46.7 ± 2.3 mV), and a polydispersity index (PDI) of 0.184 for E-R@ISSL, indicating favorable uniformity and colloidal stability. Transmission electron microscopy (TEM) further confirmed that all nanoformulations displayed regular spherical or elliptical morphologies (**Figure 3C-E**). The hydrophobic LA domain and positively charged IAA moiety dominated the particle surface, conferring a strong positive charge, whereas the hydrophilic RGD segment extended into the aqueous phase, preventing aggregation and stabilizing the uniform nanostructure. This synergistic hydrophobic-electrostatic assembly mechanism enabled efficient co-loading of both prodrug components. As a carrier-free system composed exclusively of the two prodrugs, E-R@ISSL achieved encapsulation efficiencies of 90.4 ± 1.5% and 94.2 ± 1.9% for ETP-CM-RGD and IAA-SS-LA, respectively, with corresponding drug loading proportions of 37.8 ± 1.3% and 62.2 ± 1.8%, approaching near-total loading. Moreover, the 100 ns all-atom molecular dynamics simulation revealed spontaneous co-aggregation within 30 ns (**Figure 3F**). The root-mean-square deviation (RMSD) converged to 8.10 ± 0.35 nm, signaling structural equilibration, while the radius of gyration (Rg) fell sharply within the first 40 ns before plateauing and the solvent-accessible surface area (SASA) contracted from approximately 750 nm² to 350 nm², reflecting progressive structural compaction (**Figure S1A-C**). π–π stacking among ETP-CM-RGD molecules together with intermolecular hydrogen bonding between IAA-SS-LA molecules propelled the assembly toward stable supramolecular nanoparticles with a hydrophobic core-hydrophilic shell architecture (**Figure 3G-H**). Spectroscopic data aligned well with these simulation findings. UV-Vis spectroscopy showed a red shift in the aromatic absorption peak of E-R@ISSL, suggesting enhanced π-π stacking, while Fourier-transform infrared spectroscopy recorded red shifts in the N-H/O-H and C=O vibrational bands, confirming strengthened hydrogen bonding (**Figure S1D**-**E**). These observations were highly consistent with the increased hydrogen bonding, reduced aromatic ring spacing, and structural compaction trend observed in the simulations.

Having confirmed the structural integrity of the nanoassembly, we next evaluated its dual-responsive drug release behavior. Under non-stimulatory conditions, negligible drug release was observed within 12 h (**Figure S2A**). In the presence of CES2, hydrolysis of the carbamate bond led to over 90% release of ETP within 12 h (**Figure S2B**). Under high GSH concentrations, cleavage of the disulfide bond resulted in a 95% release rate of IAA-SH (**Figure S2C**). Notably, when both stimuli coexisted, ETP and IAA-SH were released synchronously, demonstrating cooperative responsiveness (**Figure S2D-F**). LC-MS analysis further confirmed stimulus-triggered cleavage at the nanoassembly level: following incubation with 10 mM GSH + 0.01 U/mL CES2, characteristic cleavage products including ETP, RGD-NH_2_, IAA-SH, and LA-SH, were clearly detected, whereas no such products were observed under non-stimulatory conditions, confirming the chemical stability of the intact system (**Figure 3I**, and **S2G-L**). To determine whether chemical cleavage translated into nanoassembly disassembly, we monitored the hydrodynamic size distribution of E-R@ISSL under various conditions by DLS. In PBS, E-R@ISSL maintained a monomodal distribution throughout 12 h, indicating structural stability in the absence of stimuli (**Figure S3A**). Under CES2, GSH, or dual-stimulus conditions, the size distributions progressively broadened and shifted toward larger sizes, with multimodal distributions emerging, indicating compromised assembly integrity and structural rearrangement (**Figure S3B-D**). TEM imaging further confirmed that dual-stimulus treatment transformed the originally regular nanoparticles into irregular flocculent aggregates, providing morphological evidence for stimulus-induced disassembly (**Figure S3E**). To assess the translational relevance of this responsive behavior, we further examined drug release under physiologically relevant conditions. In PBS, FBS, and mouse plasma, both ETP and IAA-SH release remained low, whereas in tumor homogenate supernatant, release increased markedly in a time-dependent manner, indicating that E-R@ISSL remains relatively stable in circulation-mimicking environments while being preferentially activated in the tumor microenvironment (**Figure S3F-G**). Long-term stability evaluation over 21 days demonstrated minimal changes in hydrodynamic size and PDI across deionized water, PBS, FBS, and 10% plasma/heparin in PBS (**Figure 3J**, and **Figure S3H**). Zeta potential in deionized water remained stable, and drug-loading retention of both ETP-CM-RGD and IAA-SS-LA showed no significant decline, confirming excellent long-term physicochemical and biological colloidal stability (**Figure S3I-J**). Despite the high positive surface charge, hemolysis assays demonstrated that E-R@ISSL induced negligible hemolysis across the tested concentration range, with hemolysis rates remaining below the 5% safety threshold, supporting its blood compatibility for subsequent *in vivo* application (**Figure S3K**).

### E-R@ISSL achieved selective DNA damage and potent cytotoxicity against NSCLC cells through a dual-targeting strategy

To verify that the dual RGD/mitochondria-targeting strategy enhanced tumor-specific delivery, we first examined the cellular uptake behavior of E-R@ISSL in NSCLC cells. Flow cytometry analysis revealed a concentration-dependent increase in intracellular fluorescence intensity in both LLC and PC-9 cells, indicating efficient cellular internalization (**Figure S4A**). Time-dependent uptake experiments further demonstrated that E-R@ISSL exhibited stronger intracellular accumulation than IAA-SS-LA alone, suggesting that nanoassembly and RGD modification facilitated cellular entry (**Figure S4B**). Moreover, E-R@ISSL uptake was significantly higher in PC-9 and LLC tumor cells than in normal bronchial epithelial BEAS-2B cells, and pretreatment with excess free RGD substantially reduced this uptake, confirming RGD-mediated αvβ5 targeting (**Figure S4C**). These results established that E-R@ISSL possesses efficient and tumor-selective internalization capability.

**Figure 4:**
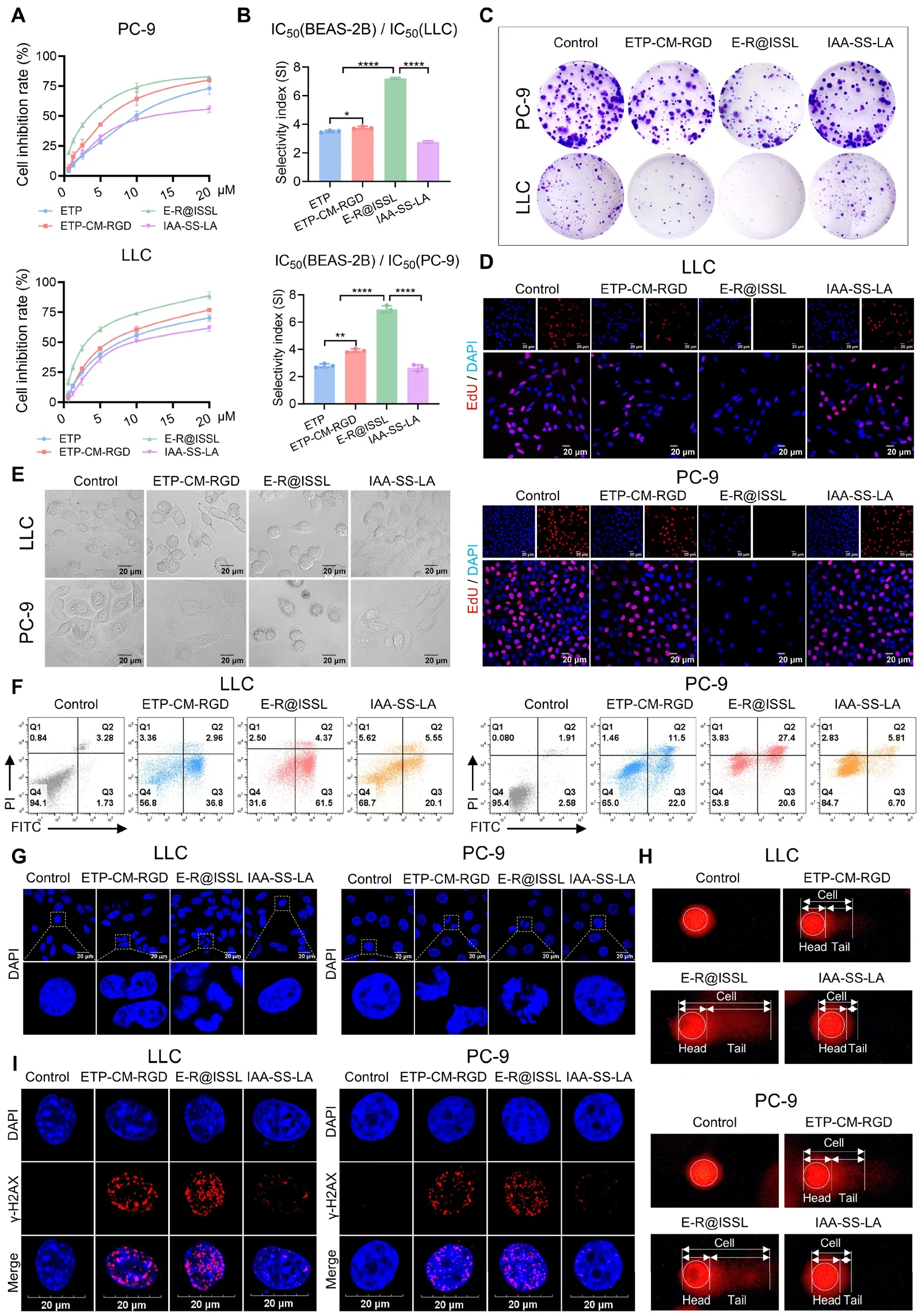
** E-R@ISSL suppressed tumor cell proliferation and triggered apoptosis by disrupting DNA integrity.** (**A**) Cytotoxicity of ETP, ETP-CM-RGD, E-R@ISSL, and IAA-SS-LA against PC-9 and LLC cell lines. (**B**) SI of different drug groups relative to BEAS-2B cells compared to PC-9 and LLC cells, respectively. (**C**) Clonogenic potential of NSCLC cells exposed to diverse pharmacological treatments. (**D**) Representative images of cell growth suppression in NSCLC cells subjected to various treatments (40X, scale bar indicates 20 μm). (**E**) Morphological changes in NSCLC cells following various treatment. (**F**) Apoptosis in NSCLC cells following different drug treatments. (**G**) Nuclear modifications in NSCLC cells following different drug treatments. (**H**) Comet tailing of NSCLC cells after different pharmacological treatments. (**I**) Immunofluorescence study of γ-H2AX in NSCLC cells post-treatment. ns, no substantial change, **p < 0.01, ***p < 0.001, ****p < 0.0001.

We next evaluated whether this enhanced tumor-cell delivery translated into superior antitumor activity. MTT assays revealed that ETP-CM-RGD exhibited stronger antiproliferative activity than free ETP, while the dual-targeted nanoformulation E-R@ISSL showed the most potent inhibitory effect in both LLC and PC-9 cells (**Figure 4A**). IAA-SS-LA alone also displayed moderate cytotoxicity, likely attributable to its cationic-hydrophobic character facilitating non-specific cellular uptake and subsequent GSH-triggered LA release; however, its potency was limited by the absence of RGD-mediated active targeting. Notably, free ETP displayed considerable toxicity toward normal BEAS-2B cells (**Figure S4D**). Further analysis using the selectivity index (SI = IC_50_ of normal cells/IC_50_ of tumor cells) revealed that E-R@ISSL possessed the highest tumor selectivity (LLC: 7.15 ± 0.13; PC-9: 6.68 ± 0.07), indicating that this nanosystem possessed favorable biosafety properties while enhancing therapeutic efficacy (**Figure 4B**). Moreover, E-R@ISSL markedly suppressed the long-term proliferative capacity of cancer cells via colony formation assays (**Figure 4C**). EdU staining further confirmed a significant decrease in the proportion of proliferating cells after 24 h treatment with E-R@ISSL (**Figure 4D**). Morphological examination revealed characteristic structural damage, including cell rounding, swelling, and fragmentation, in the E-R@ISSL-treated group (**Figure 4E**). Annexin V/PI double-staining apoptosis assays demonstrated that all drug treatments induced apoptosis, with E-R@ISSL exhibiting the strongest pro-apoptotic effect (LLC: 65.87%; PC-9: 41.37%) (**Figure 4F,** and **Figure S4E**). DAPI staining showed nuclear condensation, fragmentation, and enhanced fluorescence, indicating severe nuclear damage (**Figure 4G**).

To elucidate the underlying mechanism, DNA damage was evaluated. Comet assays revealed that E-R@ISSL induced substantial DNA strand breaks (**Figure 4H**). Concurrently, treatment with E-R@ISSL significantly elevated intracellular 8-OHdG levels (LLC: 5.33 to 27.95 ng/mL; PC-9: 3.83 to 30.22 ng/mL) (**Figure S4F**), suggesting aggravated oxidative DNA single-strand damage. γ-H2AX immunofluorescence further confirmed that E-R@ISSL effectively provoked DNA double-strand breaks, as evidenced by the most prominent formation of γ-H2AX foci (**Figure 4I**). In summary, E-R@ISSL induced both single- and double-strand DNA breaks, potently inhibited proliferation, and promoted apoptosis, thereby demonstrating remarkable *in vitro* anti-tumor activity together with excellent selectivity.

### Targeting mitochondria with E-R@ISSL triggered ROS-mediated apoptosis

**Figure 5:**
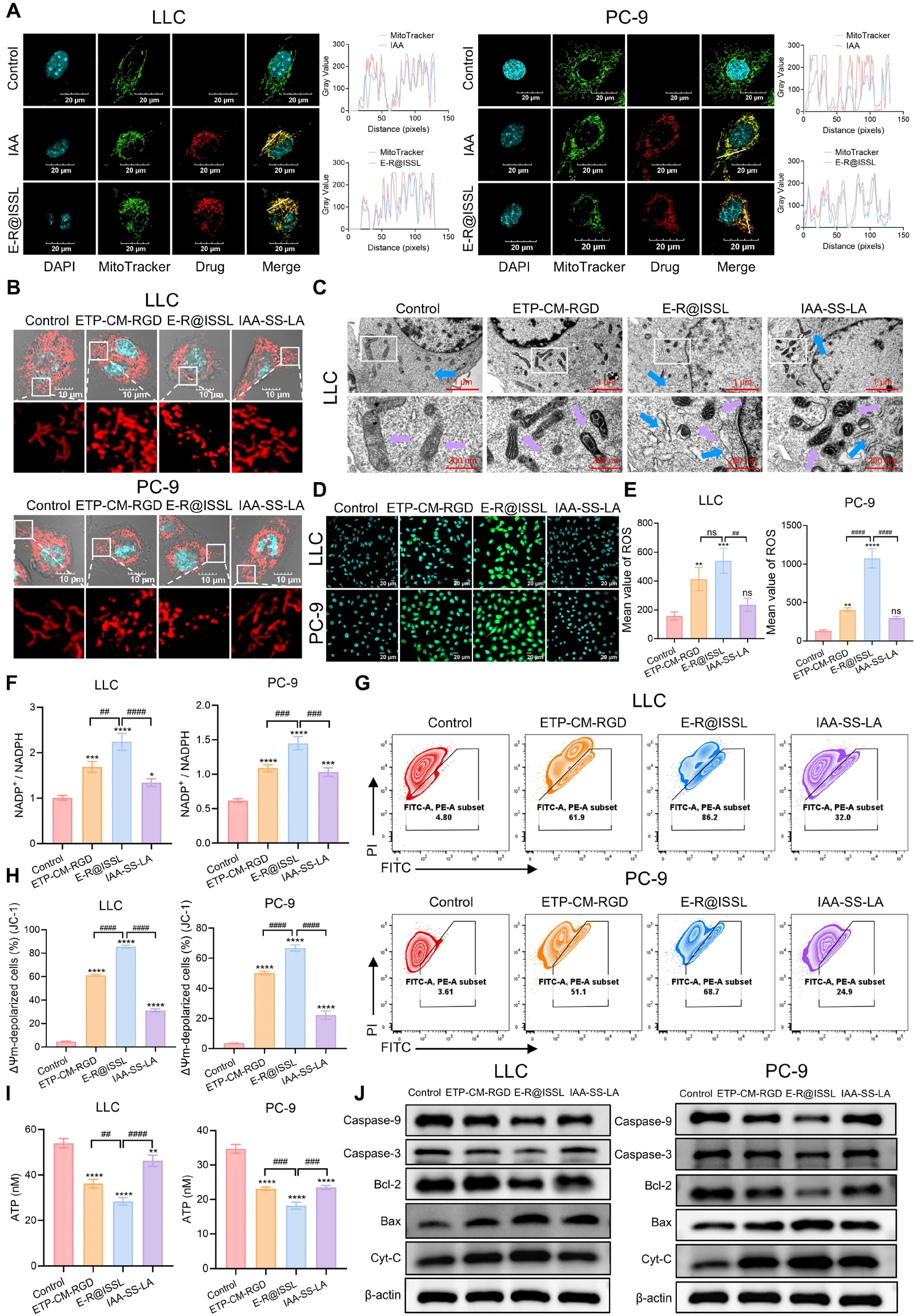
** Mitochondrial targeting and dysfunction induction by E-R@ISSL.** (**A**) Co-localization of E-R@ISSL with mitochondria in NSCLC cells (100X, scale bar indicates 20 μm). (**B**) Mitochondrial morphological changes (100X, scale bar denotes 10 μm). (**C**) Mitochondrial ultrastructural alterations observed via the TEM. (**D**) Accumulation of ROS (40X, scale bar indicates 20 μm). (**E**) Quantitative evaluation of ROS levels via the flow cytometry. (**F**) Measurement of the NADP⁺/NADPH ratio. (G) Assessment of MMP in NSCLC cells post-treatment. (**H**) Quantification of JC-1 fluorescence to assess MMP (ΔΨm). (**I**) ATP levels in NSCLC cells after the indicated treatments. (**J**) Western blot detection of Caspase-3/9, Bax, Bcl-2, and Cyt-C in LLC and PC-9 cells. ns, no substantial change, **p < 0.01, ***p < 0.001, ****p < 0.0001 vs. the control group. ^##^*p* < 0.01, ^###^*p* < 0.001, ^####^*p* < 0.0001, vs. the E-R@ISSL group.

To evaluate the mitochondrial targeting and delivery capability of the E-R@ISSL system, Mito Tracker co-localization assays in LLC and PC-9 cells revealed a pronounced overlap between the drug-derived fluorescence of E-R@ISSL and mitochondrial signals. Moreover, the corresponding line-scan intensity profiles exhibited highly consistent peak patterns, confirming the efficient mitochondrial targeting and accumulation (**Figure 5A**). Further examination of mitochondrial morphology indicated that whereas control cells exhibited continuous tubular networks, E-R@ISSL treatment induced pronounced mitochondrial fragmentation (**Figure 5B**). TEM analysis revealed ultrastructural damage, including mitochondrial swelling, cristae disorganization, and matrix rarefaction, accompanied by concomitant endoplasmic reticulum morphological abnormalities (**Figure 5C**). Both confocal live-cell imaging and flow cytometry analyses indicated that E-R@ISSL substantially promoted ROS accumulation (**Figure 5D-E** and **Figure S5A**). MitoSOX Red assays further confirmed that the ROS elevation originated predominantly from mitochondria, as evidenced by its partial reversal upon pretreatment with the mitochondria-targeted antioxidant MitoQ (**Figure S5B-C**). Concurrently, E-R@ISSL treatment markedly increased the cellular NADP⁺/NADPH ratio (LLC: 1.0 to 2.2; PC-9: 0.6 to 1.5), indicating depletion of mitochondrial reductive capacity (**Figure 5F**). JC-1 staining showed a significant increase in the proportion of cells with depolarized MMP (ΔΨm), accompanied by a notable decrease in ATP levels, collectively indicating compromised mitochondrial energy metabolism **(Figure 5G-I)**. Consistent with the observed mitochondrial dysfunction, Western blot analysis revealed that E-R@ISSL downregulated the anti-apoptotic protein Bcl-2 while upregulating the pro-apoptotic proteins Bax and cytochrome c (Cyt-C), accompanied by markedly increased levels of cleaved Caspase-9/3 (**Figure 5J**, and **S5D-E**). These results collectively indicated that E-R@ISSL activated the mitochondria-mediated apoptotic pathway.

In summary, E-R@ISSL achieved efficient mitochondrial targeting, leading to structural disruption, enhanced oxidative stress, and bioenergetic dysfunction in mitochondria, which ultimately culminated in the activation of the mitochondrial pathway of apoptosis.

### E-R@ISSL exhibited favorable targeting, antitumor activity, and safety in the LLC allograft model

**Figure 6:**
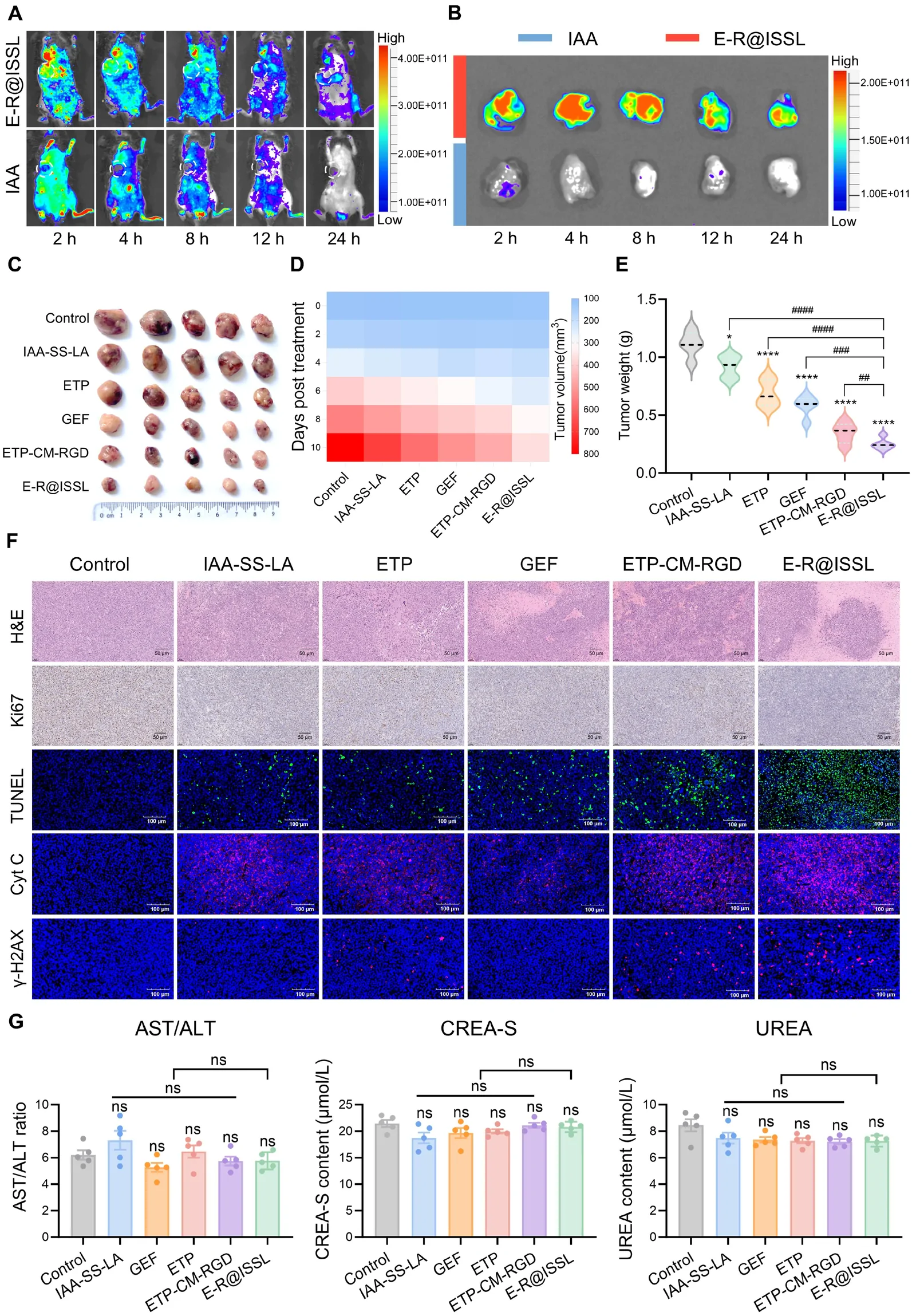
** E-R@ISSL exerted potent antitumor effects through efficient tumor targeting and favorable safety profiles.** (**A**) *In vivo* distribution of IAA and E-R@ISSL in mouse tissues via imaging techniques. (**B**) Fluorescence intensity of IAA and E-R@ISSL in tumors over time. (**C**) Representative images of tumor tissue obtained from mice after various treatments. (**D**) Variations in tumor volume in mice over time after various treatments. (**E**) Tumor weights in mice with tumors following different treatments. (**F**) Representative tumor tissue slices stained with H&E and Ki67, along with immunofluorescence staining for TUNEL, γ-H2AX, and Cyt-C. (**G**) Serum levels of AST/ALT, CREA-S, and UREA in mice receiving different therapies. Compared with the control group: ns, no significant change. **p < 0.01, ***p < 0.001, ****p < 0.0001; vs. E-R@ISSL: ^##^*p* < 0.01, ^###^*p* < 0.001, ^####^*p* < 0.0001.

The *in vivo* antitumor efficacy of E-R@ISSL was systematically evaluated in an LLC allograft model established in C57BL/6J mice. Fluorescence imaging revealed that, compared with free IAA, E-R@ISSL generated stronger and more prolonged tumor signals with retention exceeding 12 h, indicating enhanced tumor-targeted accumulation (**Figure 6A-B,** and **Figure S6A-B**). *Ex vivo* imaging of major organs further showed that E-R@ISSL was predominantly distributed in the liver at early time points and gradually cleared, whereas free IAA exhibited stronger early renal fluorescence, indicating that nanoassembly reshaped systemic biodistribution and reduced rapid renal elimination (**Figure S6C-E**). E-R@ISSL exhibited a half-life of 5.85 h, over ten-fold longer than that of free IAA (0.53 h), confirming that nanoassembly prolonged circulation and improved tumor delivery (**Figure S6F**, and **Table S1**). These pharmacokinetic gains translated into superior antitumor efficacy, as E-R@ISSL-treated mice showed the smallest tumor volumes and lowest terminal tumor weights among all groups (**Figure 6C-E**). Tumor sections from E-R@ISSL-treated mice exhibited widespread necrosis, reduced Ki67 positivity, and abundant TUNEL signals, indicating that the treatment concurrently inhibited proliferation and triggered apoptosis. Moreover, the highest levels of γ-H2AX and Cyt-C fluorescence were observed in tumors from the E-R@ISSL group, corroborating *in vivo* that the treatment elicited DNA damage and activated the mitochondrial apoptotic pathway (**Figure 6F**). Furthermore, safety evaluation indicated no significant differences in body weight or major organ weights among the groups during the treatment period (**Figure S6G-H**). Serum biochemical parameters also remained within normal ranges (**Figure 6G**). Notably, renal histology from gefitinib (GEF)-treated mice exhibited pathological alterations such as glomerular atrophy and tubular dilation, whereas no obvious toxicological changes were observed in the E-R@ISSL group (**Figure S6I**). In summary, E-R@ISSL demonstrated outstanding tumor-targeted accumulation and retention *in vivo*, and under the premise of systemic safety, exhibited potent and comprehensive antitumor efficacy.

### E-R@ISSL exerted potent antitumor activity via coordinated ferroptosis and immune activation

**Figure 7:**
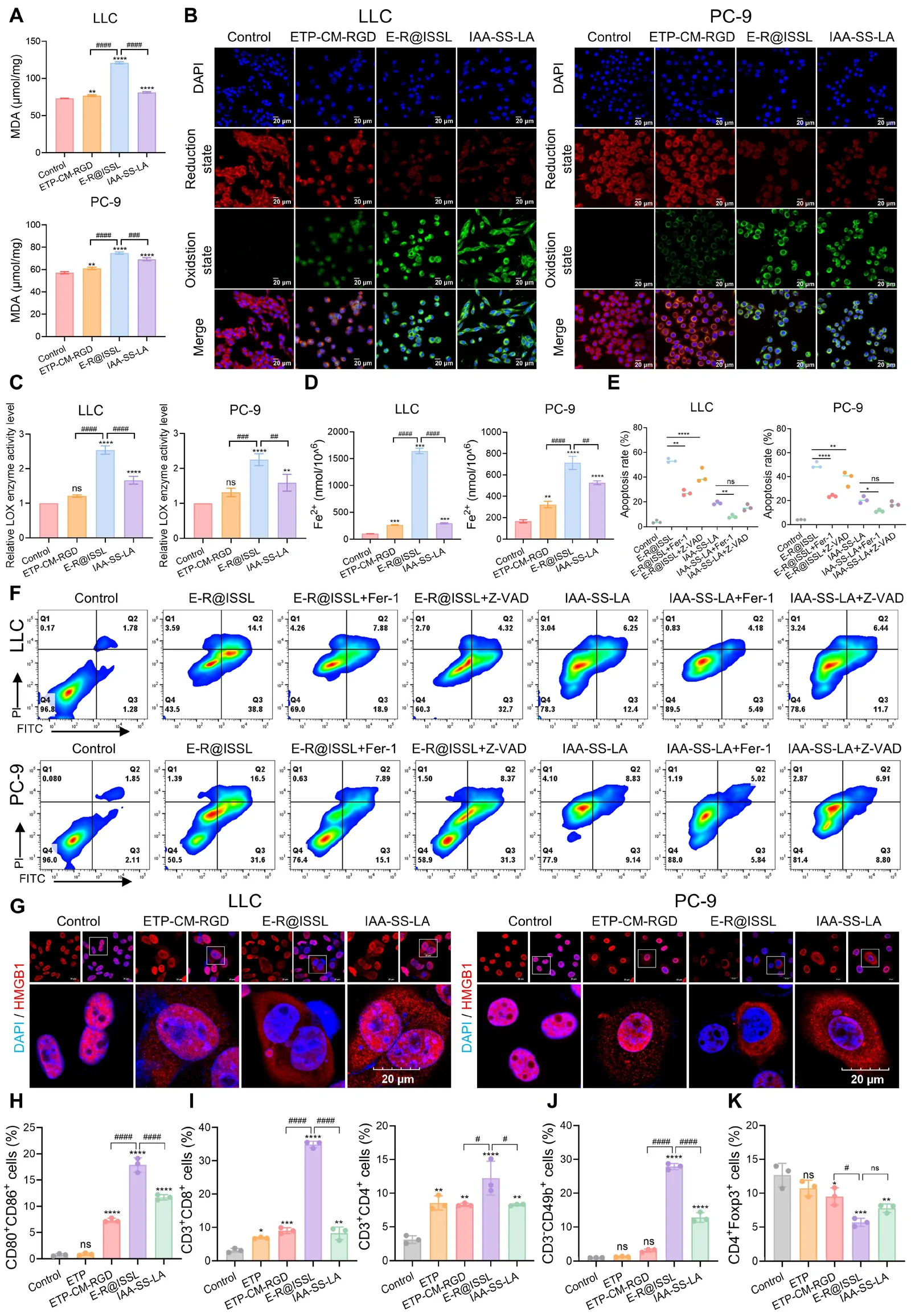
** E-R@ISSL revealed mechanisms of inducing ferroptosis, activating immune cells, and enhancing antitumor immune responses.** (**A**) Evaluation of MDA levels in NSCLC cells treated with different drugs. (**B**) Immunofluorescence imaging of lipid peroxidation in NSCLC cells induced by different drugs, scale bar = 20 μm. (**C**) Relative LOX enzyme activity alterations in NSCLC cells treated with different drugs. (**D**) Quantification of intracellular Fe^2+^ in NSCLC cells subjected to different drugs. (**E**) Quantitative assessment of apoptosis across different treatments. (**F**) Cell apoptosis in different drug treatment groups identified by flow cytometry. (**G**) Representative confocal microscopy images depicting HMGB1 release following diverse treatments. (**H**) Quantitative investigation of DC maturation (CD80⁺CD86⁺) in tumor tissue via flow cytometry. (**I**) Quantitative analysis of CD4⁺ and CD8⁺ T cell populations in tumor tissue. (**J**) Proportion of NK cells (CD3⁻CD49b⁺) in the tumor. (**K**) Representative flow cytometry quantification of regulatory T cells (Tregs, CD4⁺Foxp3⁺) in tumor tissue. vs. Control: ns, no substantial change, **p < 0.01, ***p < 0.001, ****p < 0.0001; vs. E-R@ISSL:^ ##^*p* < 0.01, ^###^*p* < 0.001, ^####^*p* < 0.0001.

An in-depth investigation into the mechanism revealed that upon E-R@ISSL treatment, intracellular GSH levels were markedly reduced, accompanied by a parallel downregulation of GPX4 protein expression (**Figure S7A-B**). Specifically, GSH concentrations decreased from 105.01 μmol/L to 31.58 μmol/L in LLC cells and from 81.16 μmol/L to 34.62 μmol/L in PC-9 cells. Notably, the disulfide bond in IAA-SS-LA functioned primarily as a GSH-responsive cleavage unit, and its thiol-disulfide exchange with intracellular GSH reduced the available GSH pool rather than directly inhibiting GPX4. Since GSH is an essential reducing cofactor for GPX4-mediated detoxification of phospholipid hydroperoxides, this GSH depletion, together with ETP-induced ROS elevation and LA-mediated expansion of the oxidizable lipid substrate pool, collectively weakened the GSH-dependent GPX4 antioxidant defense. Consistent with this impaired antioxidant capacity, E-R@ISSL-treated cells exhibited a pronounced increase in the lipid peroxidation product malondialdehyde (MDA) and enhanced lipid oxidation as confirmed by BODIPY-C11 staining (**Figure 7A-B**, and **Figure S7C**). Lipoxygenase (LOX) activity was notably increased, and intracellular Fe^2+^ accumulated substantially, indicating that E-R@ISSL disrupted intracellular iron homeostasis and expanded the labile iron pool (**Figure 7C-D**). To further verify the involvement of iron-dependent events, deferoxamine (DFO), an iron chelator, was introduced. DFO pretreatment partially reversed E-R@ISSL-induced Fe^2+^ accumulation and attenuated the enhancement of BODIPY-C11 fluorescence, suggesting that iron-dependent lipid peroxidation contributed to E-R@ISSL-mediated ferroptotic damage (**Figure S7D-F**). Specific inhibitor assays further indicated that the ferroptosis inhibitor Fer-1 could reverse the aforementioned phenotypes and improve cell viability, whereas the apoptosis inhibitor Z-VAD exhibited limited effects, establishing ferroptosis as the predominant mode of E-R@ISSL-induced cell death (**Figure 7E-F** and **Figure S7G-H**). These results indicated that E-R@ISSL simultaneously engaged both mitochondrial apoptosis and ferroptosis, with the ETP/LA synergistic cytotoxicity predominantly executed through the ferroptotic pathway. Consistent with *in vitro* findings, E-R@ISSL-treated mouse tumor tissues exhibited noticeably reduced GPX4 expression, implying the occurrence of ferroptosis* in vivo* (**Figure S7I**). Meanwhile, E-R@ISSL treatment potently induced the exposure and release of immunogenic cell death (ICD)-associated molecules, including surface translocation of calreticulin (CRT), nucleo-cytoplasmic translocation of HMGB1, and increased extracellular ATP secretion (**Figure 7G**, and **Figure S7J**, **S8A**). Compared to monocomponent treatments, ETP-CM-RGD significantly amplified ICD-associated DAMPs signals, thereby providing stronger “danger signal” input for immune activation. Building upon the observed ICD effects, E-R@ISSL effectively promoted dendritic cell maturation, as evidenced by an elevated proportion of CD80⁺CD86⁺ cells (**Figure 7H,** and **Figure S8B**). Meanwhile, E-R@ISSL treatment significantly increased intratumoral infiltration of both CD4⁺ T cells (12.23%) and CD8⁺ T cells (34.87%), reflecting effective activation and recruitment of effector T cells to the tumor (**Figure 7I** and **Figure S8C**). The proportion of NK cells (CD3⁻CD49b⁺) also markedly increased, further enhancing innate immune clearance (**Figure 7J** and **Figure S8D**). At the same time, the Treg compartment (CD4⁺Foxp3⁺) dropped from 12.67% to 5.69%, indicating substantial reversal of the intratumoral immunosuppressive milieu (**Figure 7K** and **Figure S8E**). Taken together, E-R@ISSL triggered ferroptosis and ICD in tandem, activating both innate and adaptive immune responses while reshaping the tumor immune microenvironment toward an immunostimulatory state.

### E-R@ISSL induced ferroptosis and inhibited progression in orthotopic lung cancer

**Figure 8:**
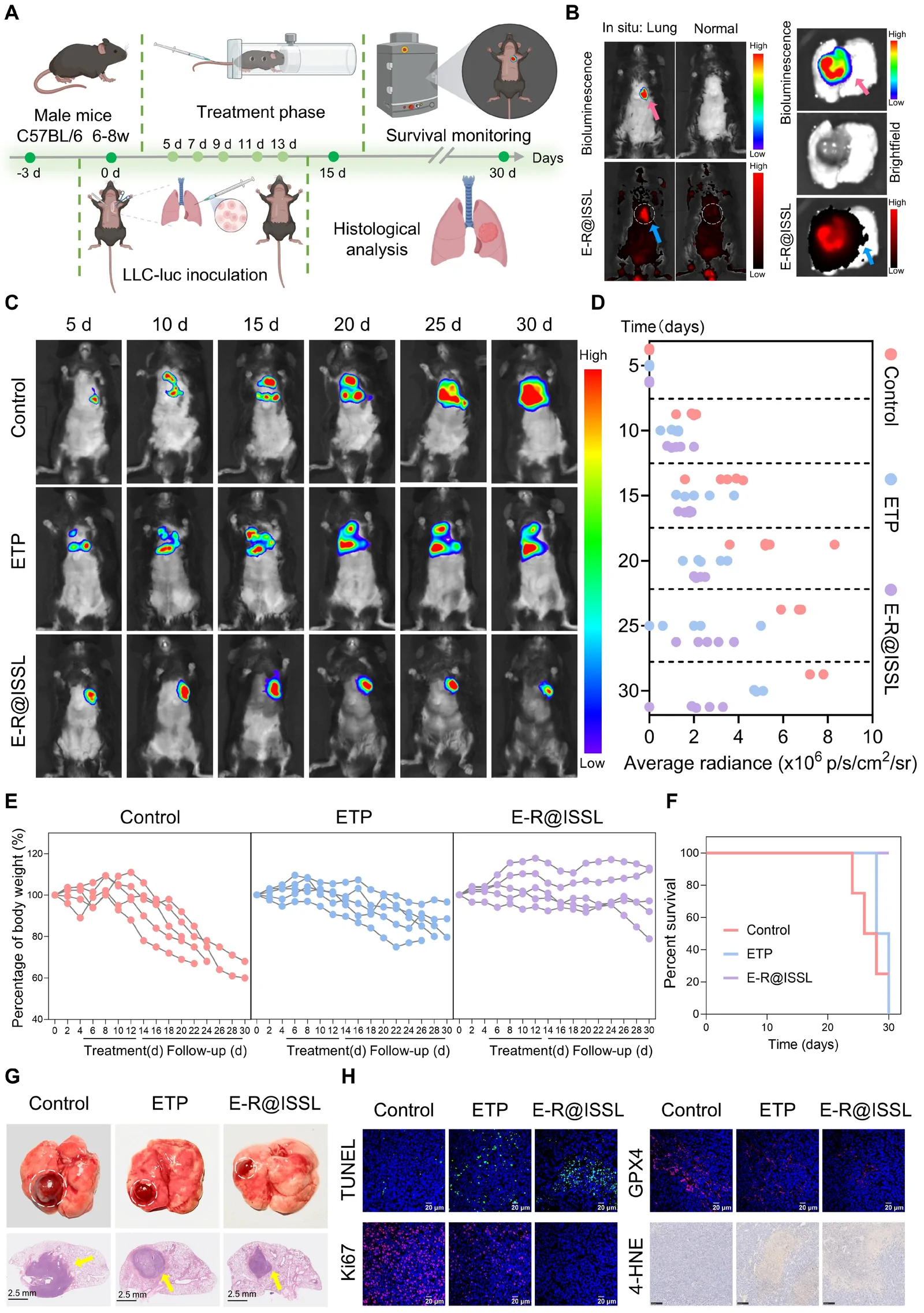
** E-R@ISSL induced ferroptosis and inhibited progression in orthotopic lung cancer.** (**A**) Schematic diagram of LLC-luc cells *in situ* orthotopic injection, followed by the timeline for subsequent treatment and analysis. (**B**) *In vivo* bioluminescence imaging demonstrating the targeting and aggregation properties of E-R@ISSL. (**C**) *In vivo* bioluminescence imaging revealed tumor burden in mice receiving different therapies at designated time intervals. (**D**) *In vivo* bioluminescence images depicting fluorescence intensity of tumor burden in mice subjected to various therapies at designated time intervals. (**E**) Body weight of mice from various treatment groups, standardized to day 0 (n=5 mice). (**F**) Kaplan-Meier survival curves for tumor-bearing mice (n=6 mice). (**G**) Tumor proliferation and H&E staining in mice pulmonary tissue. (**H**) Immunofluorescence sections (TUNEL, Ki67, GPX4) and immunohistochemical sections (4-HNE) of lung tissue. vs. Control: ns, no substantial change, **p < 0.01, ***p < 0.001, ****p < 0.0001; vs. E-R@ISSL: ^##^*p* < 0.01, ^###^*p* < 0.001, ^####^*p* < 0.0001.

The *in vivo* antitumor efficacy of E-R@ISSL was subsequently evaluated in an orthotopic lung cancer mouse model. The therapeutic agents were administered via tail vein injection, while tumor progression during treatment was monitored via IVIS spectral imaging (**Figure 8A**). Remarkably, E-R@ISSL exhibited pronounced accumulation in the lungs, whereas no aberrant aggregation was observed in the pulmonary regions of non-tumor-bearing animals, indicating a tumor-selective distribution profile of this delivery system (**Figure 8B**). Quantification of luciferase signals showed that tumor burden in untreated mice rose steeply over time. The ETP group exhibited only a modest delay in tumor growth, whereas E-R@ISSL treatment produced strong and durable antitumor suppression (**Figure 8C**-**D**).

In addition, body weight monitoring revealed pronounced weight loss in both the control and ETP groups, indicative of aggressive tumor progression and underlying systemic toxicity. In contrast, mice treated with E-R@ISSL maintained stable body weight, underscoring the favorable safety profile of this formulation (**Figure 8E**). Mortality occurred from day 22 onward in control mice and from day 28 in the ETP arm, whereas E-R@ISSL treatment prevented all deaths throughout the study, resulting in substantially higher survival rates (**Figure 8F**). These findings suggested that E-R@ISSL significantly delayed orthotopic tumor progression and improved survival outcomes. Following endpoint sampling, macroscopic examination of lung tissue morphology revealed the most pronounced tumor regression in the E-R@ISSL group, with notable tumor area reduction and comparatively lower lung tissue weight (**Figure 8G**). Furthermore, TUNEL and Ki67 staining of lung tissue sections corroborated the potent *in vivo* antitumor effects, demonstrating enhanced apoptosis and suppressed proliferation (**Figure 8H**). Mechanistically, immunohistochemical analysis revealed downregulated GPX4 expression accompanied by substantial accumulation of 4-HNE in the E-R@ISSL group, suggesting that this nanodrug disrupted GPX4-mediated anti-lipid peroxidation defenses, thereby promoting uncontrolled lipid peroxidation damage and ultimately inducing and potentiating ferroptosis in orthotopic lung tumor tissues.

### E-R@ISSL exhibited excellent biosafety* in vivo*

**Figure 9:**
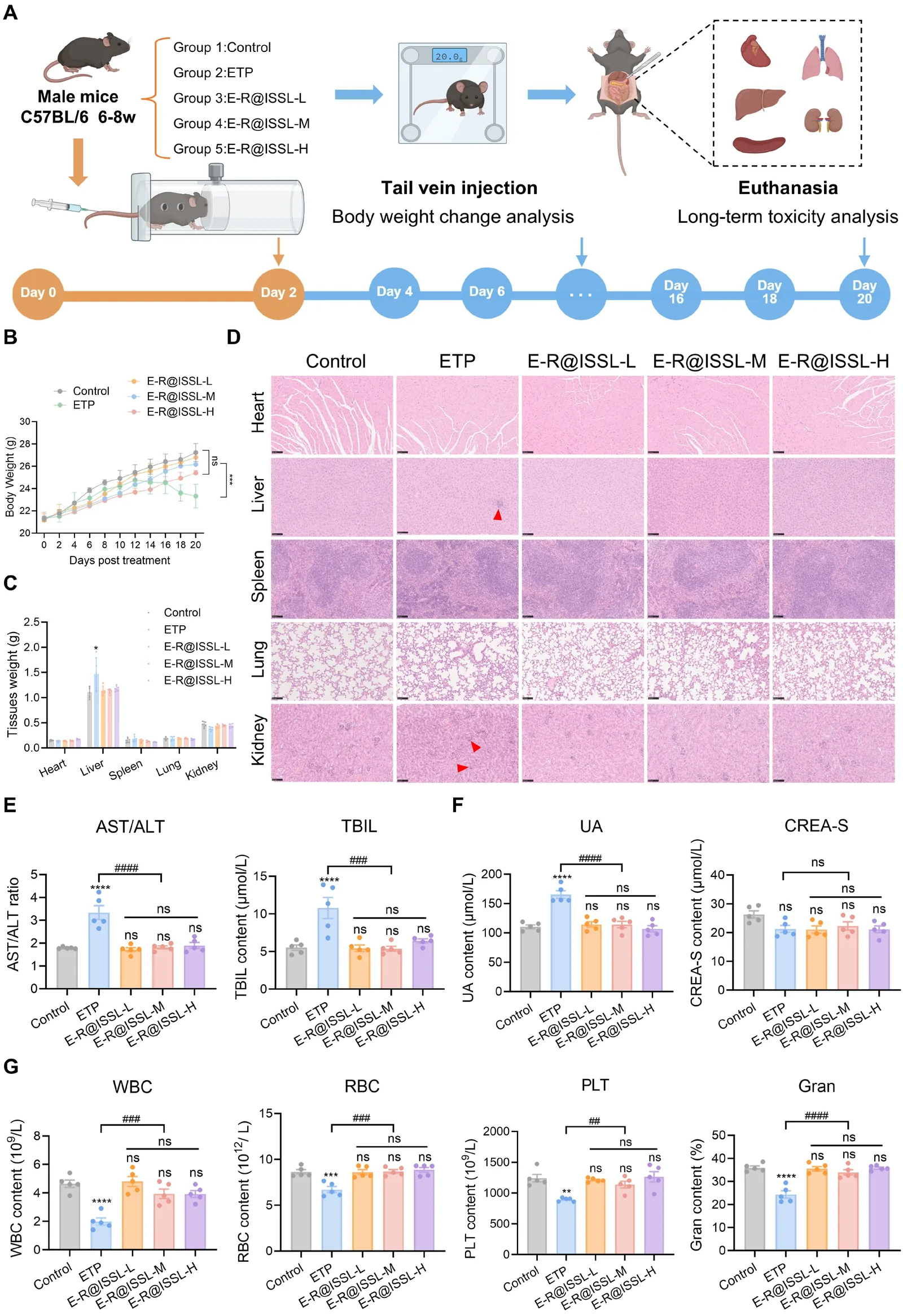
** E-R@ISSL demonstrated exceptional biocompatibility.** (**A**) Schematic diagram of the long-term toxicity model establishment and dosing regimen in mice. (**B**) Weight change curves for each group during the treatment period. (**C**) Tissue weights of heart, liver, spleen, lung and kidney in different treatment groups. (**D**) H&E staining images of major organs (heart, liver, spleen, lung and kidney), scale bar = 100 μm. (**E**) Serum AST/ALT, TBIL levels in mice following different treatments. (**F**) Serum CREA-S, UA levels in mice following different treatments. (**G**) Changes in hematological parameters (WBC, RBC, PLT, and Gran) after different treatments. vs. Control: ns, no substantial change, **p < 0.01, ***p < 0.001, ****p < 0.0001; vs. E-R@ISSL: ^##^*p* < 0.01, ^###^*p* < 0.001, ^####^*p* < 0.0001.

In the assessment of the *in vivo* antitumor efficacy of E-R@ISSL, no significant systemic toxicity was observed. To systematically evaluate its *in vivo* safety profile, a long-term administration study was conducted in C57 mice (**Figure 9A**). During the experimental period, mice in the free ETP group exhibited a decrease in body weight on day 14 of treatment, whereas body weight in all other groups showed a consistent upward trend. Notably, none of the E-R@ISSL dosage groups resulted in significant weight loss, indicating favorable overall tolerability of the formulation (**Figure 9B**). Analysis of organ weights revealed no statistically significant differences in the heart, liver, spleen, lungs, or kidneys between the control group and the low-, medium-, or high-dose E-R@ISSL groups. In contrast, the free ETP group displayed mild hepatomegaly (**Figure 9C**). Systemic toxicity was further assessed by H&E staining of major tissues together with hematological and serum biochemical assays. Liver and kidney sections from free ETP-treated mice showed prominent inflammatory cell infiltration, suggesting drug-induced local injury. In all E-R@ISSL dose groups, by contrast, no pathological changes were evident in these organs (**Figure 9D**). Serum biochemistry confirmed that free ETP markedly raised AST/ALT ratio and TBIL level, consistent with significant hepatic injury. In the E-R@ISSL groups, by contrast, all liver function markers stayed within normal limits, showing no evidence of hepatotoxicity (**Figure 9E**). Furthermore, free ETP caused a significant increase in uric acid (UA), whereas the E-R@ISSL-treated groups showed no notable changes in creatinine (CREA-S) or UA, demonstrating that the nanoformulation effectively alleviated renal burden (**Figure 9F**). Myelosuppression represents the dose-limiting toxicity of etoposide. Hematological parameters further confirmed the toxicity-mitigating effect of E-R@ISSL: the free ETP group exhibited significantly reduced white blood cell (WBC), red blood cell (RBC), platelet (PLT), and granulocyte (Gran) counts compared with the control group. In contrast, all hematological indices in the E-R@ISSL dosage groups showed no statistically significant differences from the control group, indicating no apparent myelosuppression (**Figure 9G**). In summary, E-R@ISSL demonstrated excellent biosafety *in vivo* and effectively mitigated the systemic toxicity induced by free etoposide.

## Conclusion

This study successfully developed a self-assembled nano-prodrug, E-R@ISSL, which integrated dual-targeting capabilities toward tumor cells and mitochondria, and enabled a cascade of responsive release specifically within tumor cells. The nano-prodrug was designed to synergistically amplify dual stresses: DNA damage and lipid peroxidation, thereby inducing ICD to enhance the comprehensiveness and durability of antitumor efficacy.

After selective αvβ5-mediated internalization by NSCLC cells, the nano-prodrug underwent CES2/GSH-coordinated disassembly that depleted intracellular GSH, disrupted redox homeostasis, and weakened GPX4-dependent antioxidant defense, thereby establishing conditions permissive for lipid peroxidation. ETP, upon release, poisoned TOP-II and caused DNA double-strand breaks that activated the apoptotic pathway. The freed LA entered membrane phospholipids and greatly enlarged the pool of oxidizable lipid substrates. Meanwhile, ETP-induced ROS created a strongly oxidizing intracellular environment. Together, under a functionally impaired GSH/GPX4 antioxidant defense, these two inputs converged to sustain an autocatalytic chain reaction and uncontrolled LPO buildup, pushing cells into ferroptosis. Mitochondrial targeting by IAA additionally channeled this oxidative assault onto the organelle at the core of energy metabolism and apoptosis, exacerbating mitochondrial dysfunction and thereby amplifying the overall cell death signal at the subcellular level. Concomitant membrane disruption and cellular stress triggered the release of immunostimulatory DAMPs, including CRT surface translocation, ATP secretion, and HMGB1 release, which collectively promoted dendritic cell maturation and initiated antigen-specific T-cell responses. This cascade may transform local chemo-ferroptotic cytotoxicity into systemic antitumor immunity, offering a strategy to overcome the immunosuppressive tumor microenvironment and achieve durable therapeutic benefits 38.

In an orthotopic lung cancer model, E-R@ISSL demonstrated significant tumor growth suppression, prolonged survival, and a favorable safety profile, validating its therapeutic potential. This work uniquely chains apoptosis, ferroptosis, and ICD into a single therapeutic cascade, thereby converting direct tumor cell killing into amplified antitumor immunity. Unlike typical co-delivery nanoparticles or ferroptosis-focused platforms that rely on a single trigger or targeting mechanism, E-R@ISSL combines carrier-free self-assembly with dual αvβ5/mitochondria targeting and sequential CES2/GSH-responsive drug liberation, culminating in mitochondria-localized oxidative stress amplification. These features position E-R@ISSL as particularly suitable for redox-adapted or therapy-resistant NSCLC, and its ICD-promoting effect also provides a rational basis for future combination with immune checkpoint inhibitors such as anti-PD-1/PD-L1 therapy. Nevertheless, further studies are still required to optimize administration routes, dosing regimens, and treatment schedules, and to validate long-term safety and translational feasibility in patient-derived models, humanized immune models, and large-animal systems. This work provides a novel nanomedicine paradigm for NSCLC combination therapy, characterized by profound mechanistic insight and promising translational prospects.

## Supplementary Material

Supplementary materials and methods, figures, ^1^H NMR and ^13^C NMR of synthesized compounds.

## Data Availability

All data are available in the main text or the supplementary materials.

## Abbreviations

CES: carboxylesterase
CRT: calreticulin
Cyt-C: Cytochrome C
ETP: etoposide
GSH: glutathione
GPX4: glutathione peroxidase 4
Gran: granulocyte
HSA: highest single agent
IAA: indole-aza-anthocyanin
ICD: immunogenic cell death
LA: linoleic acid
LPO: lipid peroxidation
LOX: lipoxygenase
LUAD: lung adenocarcinoma
LUSC: lung squamous cell carcinoma
MDA: malondialdehyde
NSCLC: non-small cell lung cancer
PLT: platelet
PDI: polydispersity index
PUFA: polyunsaturated fatty acids
ROS: reactive oxygen species
RBC: red blood cell
RMSD: root-mean-square deviation
SI: selectivity index
SASA: solvent-accessible surface area
TEM: transmission electron microscopy
UA: uric acid
WBC: white blood cell
