# Supplementary material for: Spatiotemporally programmed hybrid nano-prodrugs orchestrate multidimensionally synergistic cascade lethality in NSCLC
Source: Theranostics. 2026 Aug 24;16(15):8808–26. doi: 10.7150/thno.135204 (PMC13549233; doi:10.7150/thno.135204)
Supplement: Supplementary file 1 [file thnov16p8808s1.pdf]

Supplementary Information

Table of Contents

1. Materials and methods 3

1.1. Materials and reagents 3

1.2. Compounds synthesis and identification 4

1.3. Combination drug treatment analysis 8

1.4. Preparation of E-R@ISSL 8

1.5. Molecular dynamics simulation of nanoparticle assembly and spectroscopic evaluation 8

1.6. *In vitro* verification of CES2/GSH-responsive drug release mechanisms 9

1.7. Dynamic light scattering (DLS) analysis under stimulatory conditions and TEM observation after stimulation 9

1.8. Responsive release behavior under physiologically relevant conditions 10

1.9. Evaluation of colloidal stability of E-R@ISSL 10

1.10. Hemolysis assay of E-R@ISSL 10

1.11. Difference in protein expression between ITGAV and ITGB5 11

1.12. Immunohistochemical examination of ITGAV and ITGB5 in pulmonary carcinoma 11

1.13. Detection of receptor expression 11

1.14. Molecular docking 11

1.15. Differential expression analysis of GSH-related genes 11

1.16. GSH detection and analysis 11

1.17. Cell viability assay 11

1.18. Colony formation assay 11

1.19. Cell morphology observation 12

1.20. Cell proliferation assay 12

1.21. Cell apoptosis assay 12

1.22. Nuclear morphological changes 12

1.23. Immunofluorescence analysis of γ-H2AX 12

1.24. Comet assay 12

1.25. Analysis of human 8-hydroxydeoxyguanosine (8-OHdG) 13

1.26. MitoTracker staining and confocal colocalization 13

1.27. Mitochondrial morphological changes 13

1.28. Reactive oxygen species (ROS) assay 13

1.29. Determination of $NADP^+$/NADPH levels and the $NADP^+$/NADPH ratio 13

1.30. Mitochondrial membrane potential (MMP) assay 13

1.31. ATP detection and analysis 13

1.32. Western blot analysis 14

1.33. Tumor model 14

1.34. *In vivo* imaging of mice 14

1.35. Pharmacokinetic analysis 14

1.36. Hematoxylin and eosin (H&E) and immunohistochemistry 14

1.37. Immunofluorescence analysis of HMGB1/CRT 15
1.38. Measurement of lipid peroxidation (MDA) levels 15
1.39. Immunofluorescence analysis of GPX4 15
1.40. Confocal Imaging of lipid peroxidation using BODIPY 581/591 C11 15
1.41. Measurement of lipoxygenase (LOX) activity 15
1.42. Flow cytometric analysis of intracellular ferrous iron (Fe^{2+}) 15
1.43. Flow cytometric analysis of apoptosis 16
1.44. Flow cytometry analysis of tumor immune microenvironment 16
1.45. Creation of an orthotopic LLC-luc lung tumor model and *in vivo* bioluminescence imaging 16
1.46. Hematological assessment in murine models 17
1.47. Statistical analysis 17
2. Supplementary Figures 17
3 ^{1}H NMR (A) and ^{13}C NMR (B) of final compounds 32

1. Materials and methods

1.1. Materials and reagents

Etoposide (ETP, ≥ 98%) and gefitinib (CAS 184475-35-2) were bought from Shanghai Dibai Biotechnology Co., Ltd. and Shanghai Macklin Biochemical Co., Ltd., respectively (Shanghai, China). Linoleic acid (LA, ≥ 98%) was procured from Shanghai Bide Pharmaceutical Technology Co., Ltd. (Shanghai, China). Unless stated otherwise, all other reagents (Aladdin or Yuanye, Shanghai, China) were of ≥ 95% purity and used as received. Analytical-grade solvents were supplied by Chengdu Jinshan Chemical Reagent Co., Ltd. The reactions were monitored via thin-layer chromatography (TLC) on silica gel GF254 plates (UV, 254 nm). All products underwent purification by flash column chromatography utilizing silica gel (200-300 mesh). High-performance liquid chromatography (HPLC) analysis was conducted on a Shimadzu LC-20AT system (SPD-16 UV-vis detector, 280 nm). High-resolution mass spectra (HRMS) were obtained utilizing an Agilent 1260-Bruker tims TOF mass spectrometer with electrospray ionization (ESI). ^{1}H and ^{13}C NMR spectra were recorded on Bruker 600 or 700 MHz spectrometers. Chemical shifts (δ, ppm) are referenced to tetramethylsilane (TMS) with residual solvent peaks as internal standards ($CDCl_3$: δ 7.26 ppm for ^{1}H, δ 77.2 ppm for ^{13}C; CD_3OD-d_4: δ 3.31 ppm for ^{1}H, δ 49.0 ppm for ^{13}C; DMSO-d_6: δ 2.50 ppm for ^{1}H, δ 39.5 ppm for ^{13}C). Signal multiplicities are classified as follows: singlet (s), doublet (d), triplet (t), double doublet (dd), double triplet (dt), triple doublet (td), quartet doublet (qd), wide signal (br), and multiplet (m). Coupling constants (*J*) are expressed in hertz (Hz).

The integrin αvβ5 antibody was from Shanghai YaJi Biological Technology (Shanghai, China; Catalog No. Ys-1356R); 5-FITC-(Acp)-RGD from Qiangyao Biotechnology Co., Ltd.; PKMITO™ Deep Red (PKMDR-2) from Nanjing Puhai Jingshan Biotechnology Co., Ltd. (Nanjing, China). AF488-labeled goat anti-rabbit IgG (H+L) (Cat. No. A0423), BeyoClick™ EdU-647 Cell Proliferation Kit (Cat. No. C0081S), Annexin V-FITC/PI Apoptosis Detection Kit (Cat. Nos. C1062L/C1062S), DNA Damage Assay Kit (γ-H2AX; Cat. No. C2036S), Comet Assay Kit (Cat. No. C2041M), Reactive Oxygen Species (ROS) Assay Kit (Cat. No. S0033S), $NADP^+$/NADPH Assay Kit with WST-8 (Cat. No. S0179), Mitochondrial Membrane Potential Assay Kit with JC-1 (Cat. No. C2006), ATP Assay Kit (Cat. No. S0026), Lipid Peroxidation MDA Assay Kit (Cat. No. S0131S), Lipid Peroxidation Assay Kit (BODIPY 581/591 C11; Cat. No. S0043S), and Lipoxygenase Activity Assay Kit (Fluorometric; Cat. No. P0365S) were acquired from Beyotime Biotechnology (Shanghai, China). The DAPI staining reagent was from Boster Biological Technology (Wuhan, China; Catalog No. AR1177). The Reduced Glutathione (GSH) Content Assay Kit was from Solarbio Science & Technology Co., Ltd. (Beijing, China; Catalog No. BC1175). Thiazolyl Blue Tetrazolium Bromide (MTT, >98%, CAS 298-93-1) was from Sigma. The Human 8-Hydroxydeoxyguanosine (8-OHdG) ELISA Kit (Catalog No. E-EL-0028) and the Cell Ferrous Iron (Fe^{2+}) Fluorometric Assay Kit (Catalog No. E-BC-F101) were from Elabscience Biotechnology in Wuhan, China. Anti-Caspase 9/CASP9 antibody (A00080-7), Anti-BCL2 antibody (BA0412), Anti-BAX antibody (A00183), and Anti-Cytochrome c/CYCS antibody (BA0781) were from Wuhan Boster Biological Technology Co., Ltd. (Wuhan, China). The Omni-Easy™ One-Step PAGE Gel Fast Preparation Kit was from Yeasen Biotechnology (Shanghai, China; Catalog No. PG210). Collagenase IV (1 mg/mL) and DNase I (0.1 mg/mL) were from Biosharp (China). For flow cytometry, Fixable Viability Stain 780 (Catalog No. 565388) and Purified Rat Anti-Mouse CD16/CD32 (Catalog No. 553141; BD OptiBuild™, BD Biosciences, San Jose, CA, USA) were utilized. The APC anti-mouse F4/80 antigen (Catalog No. 20-4801-U100) was acquired from Tonbo Biosciences, located in San Diego, CA, USA. FITC anti-mouse CD11c (Cat. No. 35-0114-U100), APC anti-mouse CD80 (Cat. No. 20-0801-U025), PE anti-mouse CD86 (Cat. No. 50-0862-U100), PE anti-mouse CD3 (Cat. No. 50-0032-U100), FITC anti-mouse CD4 (Cat. No. 35-0041-U100), and FITC anti-mouse CD49b (Cat. No. 35-0491-U025) were procured from Suzhou 4A Biotech Co., Ltd. (Suzhou, China).

1.2. Compounds synthesis and identification

Synthesis of compound ETP-PNP:

Etoposide (588.0 mg, 1.0 mmol, 1.0 equivalent) and triethylamine (4.0 mL, 4.0 mmol, 4.0 equivalents) were dissolved in anhydrous THF (20 mL) and cooled to 0 °C. A solution of 4-nitrophenyl chloroformate (300.0 mg, 1.5 mmol, 1.5 equivalents) in THF (10 mL) was added dropwise with stirring. The reaction mixture was subsequently allowed to equilibrate to room temperature and agitated for 4 h. The reaction was monitored by TLC. After completion, the solvent was eliminated under reduced pressure, and the crude residue was purified using silica gel column chromatography (PE/EA=1:5), yielding compound **8** as a white powder (737 mg, 98%). ^{1}H NMR (600 MHz, $CDCl_3$) δ 8.27 (dd, J = 9.3, 0.9 Hz, 2H), 7.46 (d, J = 9.2 Hz, 2H), 6.86 (s, 1H), 6.52 (s, 1H), 6.32 (s, 2H), 6.02 – 5.95 (m, 2H), 4.94 (d, J = 3.4 Hz, 1H), 4.73 (q, J = 5.0 Hz, 1H), 4.63 (d, J = 5.3 Hz, 1H), 4.62 – 4.58 (m, 1H), 4.42 (dd, J = 10.6, 8.8 Hz, 1H), 4.24 (t, J = 8.2 Hz, 1H), 4.17 (dd, J = 10.4, 3.8 Hz, 1H), 3.74 (s, 6H), 3.66 – 3.60 (m, 1H), 3.57 (d, J = 9.7 Hz, 1H), 3.44 – 3.34 (m, 3H), 3.34 – 3.26 (m, 2H), 2.87 (dddd, J = 14.1, 10.9, 7.8, 3.4 Hz, 1H), 1.37 (d, J = 5.0 Hz, 3H). ^{13}C NMR (151 MHz, $CDCl_3$) δ 175.05, 155.64, 151.29, 150.23, 148.86, 147.34, 145.40, 138.87, 132.18, 128.29, 127.78, 125.31, 121.65, 110.70, 109.30, 107.57, 101.79, 101.75, 99.80, 79.80, 73.31, 72.97, 68.06, 68.01, 66.39, 56.28, 43.96, 41.02, 37.59, 20.26. HRMS (ESI) m/z caled for: $C_{36}H_{35}NO_{17}H$ $[M+H]^+$:754.1978, found 754.1985.

Synthesis of compound 1: Boc-Glycine (3.00 g, 17.14 mmol, 1.2 equiv) was solubilized in DCM (60 mL) and cooled in an ice bath. EDCI (4.90 g, 25.68 mmol, 1.5 equivalents), HOBt (3.50 g, 25.68 mmol, 1.5 equivalents), DIPEA (3.3 g, 25.68 mmol, 1.0 equivalent), and NMM (2.60 g, 25.68 mmol, 1.5 equivalents) were gradually introduced under stirring, followed by L-aspartic acid dibenzyl ester p-toluenesulfonate (7.40 g, 15.41 mmol, 0.9 equivalent). The reaction mixture was agitated at ambient temperature for 8 h. Following completion, as monitored by TLC, the solvent was evaporated under reduced pressure. Ethyl acetate was added to dissolve the residue, and the solution was sequentially washed with water, 5% HCl, 5% $NaHCO_3$, and saturated NaCl. The organic layer was then dehydrated using anhydrous Na_2SO_4, filtered, and concentrated under reduced pressure. The crude product was purified using silica gel column chromatography (PE/EA=1:1), yielding a white oil (7.20 g, 90.0% yield). ^{1}H NMR (700 MHz, $CDCl_3$) δ 7.40 (d, J = 8.1 Hz, 1H), 7.34 – 7.22 (m, 10H), 5.59 (s, 1H), 5.12 – 5.06 (m, 2H), 5.02 (q, J = 12.2 Hz, 2H), 4.96 – 4.90 (m, 1H), 3.87 – 3.70 (m, 2H), 3.03 (dd, J = 17.3, 4.9 Hz, 1H), 2.90 (dd, J = 17.5, 4.7 Hz, 1H), 1.43 (d, J = 3.4 Hz, 9H). ^{13}C NMR (176 MHz, $CDCl_3$) δ 170.65, 170.44, 169.85, 156.12, 135.40, 135.20, 128.61, 128.59, 128.43, 128.36, 128.23, 79.95, 67.51, 66.80, 48.69, 44.05, 36.33, 28.34. HRMS (ESI) m/z caled for: $C_{25}H_{30}N_2O_7$ $Na[M]^+$: 493.1950, found 493.1948.

Synthesis of compound 2: Compound **1** (5.0 g, 10.63 mmol, 1.0 equiv) was dissolved in DCM (20 mL), and trifluoroacetic acid (10 mL) was added dropwise with stirring at room temperature The reaction mixture was stirred for 1 h at room temperature. After completion, the solvent was evaporated under reduced pressure to obtain compound **2** as a white solid (4.9 g, 96% yield), which was utilized immediately in the subsequent step without additional purification.

Synthesis of compound 3: N-Boc-N′-NO_2-L-arginine (3.00 g, 9.40 mmol, 1.1 equiv) was solubilized in DCM (30 mL) and subjected to cooling in an ice bath. EDCI (4.90 g, 25.65 mmol, 1.5 equivalents), HOBt (3.46 g, 25.65 mmol, 1.5 equivalents), and DIPEA (7.43 mL, 42.74 mmol, 2.5 equivalents) were sequentially introduced under

agitation, followed by the addition of compound **2** (8.27 g, 17.10 mmol, 1.0 equivalent). The mixture was stirred at room temperature for 12 h. Once TLC indicated completion, the solvent was evaporated under reduced pressure. The crude residue was dissolved in ethyl acetate and subsequently washed with water, 5% HCl, 5% $NaHCO_3$, and saturated NaCl. The organic layer was dehydrated using anhydrous Na_2SO_4, subjected to filtration, and concentrated under reduced pressure. The crude product underwent purification via silica gel column chromatography (DCM/MeOH = 30:1), yielding a pale-yellow solid (6.90 g, 60% yield). ^{1}H NMR (600 MHz, DMSO-d_6) δ 8.51 (s, 1H), 8.43 (d, J = 8.0 Hz, 1H), 8.10 (t, J = 5.7 Hz, 1H), 7.40 – 7.29 (m, 10H), 6.97 (d, J = 7.8 Hz, 1H), 5.09 (d, J = 11.9 Hz, 4H), 4.78 (dt, J = 8.1, 6.6 Hz, 1H), 3.96 (q, J = 5.2 Hz, 1H), 3.82 – 3.71 (m, 2H), 3.14 (q, J = 6.5 Hz, 2H), 2.92 (dd, J = 16.6, 6.3 Hz, 1H), 2.81 (dd, J = 16.6, 6.8 Hz, 1H), 1.72 – 1.64 (m, 1H), 1.52 (t, J = 8.7 Hz, 3H), 1.38 (s, 9H).^{13}C NMR (151 MHz, DMSO-d_6) δ 172.71, 170.76, 170.23, 169.32, 155.93, 136.26, 136.13, 128.89, 128.53, 128.51, 128.42, 128.17, 79.63, 78.69, 66.82, 66.41, 54.37, 49.06, 42.11, 36.31, 28.65. HRMS (ESI) m/z caled for: $C_{31}H_{41}N_7O_{10}Na$ $[M+Na]^+$:694.2812, found 694.2818.

Synthesis of compound 4: Compound **4** (5.00 g, 7.45 mmol, 1.0 eq) was dissolved in DCM (20 mL), and trifluoroacetic acid (10 mL) was added dropwise while stirring at ambient temperature. The reaction mixture was agitated for 1 h at room temperature. After completion, the solvent was removed under decreased pressure, yielding compound **4** as a white solid (4.60 g, 90% yield), which was utilized immediately in the subsequent step without additional purification.

Synthesis of compound 5: 7-Aminoheptanoic acid (500 mg, 3.45 mmol, 1.0 eq) was dissolved in DCM (20 mL) and cooled in an ice bath. HATU (2.0 g, 5.17 mmol, 1.5 eq) and DIPEA (1.50 mL, 8.6 mmol, 2.5 eq) were added successively, followed by compound **4** (2.6 g, 3.79 mmol, 1.1 eq). The reaction mixture was stirred at room temperature for 12 h. Upon completion (monitored by TLC), the mixture was diluted with DCM (20 mL) and successively washed with 0.2 M aqueous HCl (20 mL), saturated NaCl solution (20 mL), and water (20 mL). The organic layer was dried over anhydrous Na_2SO_4, filtered, and concentrated under reduced pressure. The crude product was purified by silica gel column chromatography (DCM/MeOH = 30:1) to afford compound **5** as a colorless solid (1.90 g, 70% yield). ^{1}H NMR (600 MHz, DMSO-d_6) δ 8.56 – 8.46 (m, 1H), 8.38 (d, J = 8.0 Hz, 1H), 8.24 (t, J = 5.9 Hz, 1H), 8.03 (dd, J = 7.5, 2.0 Hz, 1H), 7.36 (d, J = 6.9 Hz, 4H), 7.33 (d, J = 7.1 Hz, 6H), 6.73 (t, J = 5.9 Hz, 1H), 5.10 (s, 2H), 5.07 (s, 2H), 4.78 (q, J = 7.0 Hz, 1H), 4.21 (d, J = 6.9 Hz, 1H), 3.79 – 3.68 (m, 2H), 3.19 – 3.11 (m, 2H), 2.89 (tt, J = 13.7, 6.6 Hz, 4H), 2.81 (ddd, J = 16.6, 6.9, 1.6 Hz, 1H), 2.11 (h, J = 6.9 Hz, 2H), 1.66 (d, J = 13.0 Hz, 1H), 1.59 – 1.50 (m, 2H), 1.49 – 1.44 (m, 3H), 1.37 (s, 10H), 1.25 – 1.19 (m, 6H). ^{13}C NMR (151 MHz, DMSO-d_6) δ 174.98, 173.19, 172.50, 170.79, 170.22, 169.33, 156.06, 136.26, 136.16, 128.88, 128.52, 128.49, 128.39, 128.15, 77.77, 66.78, 66.39, 52.90, 49.00, 36.22, 35.51, 34.07, 29.83, 29.37, 28.87, 28.74, 26.53, 25.60, 24.92. HRMS (ESI) m/z caled for: $C_{38}H_{54}N_8O_{11}Na$ $[M+Na]^+$: 821.3809, found 821.3816.

Synthesis of compound 6: Compound **5** (1.00 g, 1.25 mmol, 1.0 eq) was solubilized in DCM (10 mL), then trifluoroacetic acid (5 mL) was incrementally added while stirring at ambient temperature. The reaction mixture was agitated for 1 h at room temperature. Upon completion, the solvent was evaporated under reduced pressure to obtain compound **6** as a white solid (915.5 mg, 90% yield), which was utilized directly in the subsequent step without additional purification.

Synthesis of compound 7: ETP-PNP (200 mg, 0.26 mmol, 1.0 equiv) and compound **6** (215 mg, 0.26 mmol,

1.0 equiv) were solubilized in anhydrous DMF (10 mL) with dry triethylamine (0.5 mL). The reaction mixture was agitated at ambient temperature for 12 h. Upon completion, the mixture was diluted with 20 mL of dichloromethane and subsequently washed with 20 mL of 0.2 M aqueous HCl, saturated brine, and water. The organic layer was dehydrated using anhydrous Na_2SO_4, filtered, and concentrated under reduced pressure to provide the crude product. The residue underwent purification via flash chromatography (DCM/MeOH = 30:1), resulting in compound 7 as a white solid (209.6 mg, 60% yield). ^{1}H NMR (600 MHz, MeOD-d_4) δ 7.35 – 7.27 (m, 10H), 6.98 (s, 1H), 6.50 (s, 1H), 6.33 (s, 2H), 5.94 (dd, J = 3.5, 1.2 Hz, 2H), 5.13 – 5.04 (m, 4H), 5.02 (d, J = 3.4 Hz, 1H), 4.90 (t, J = 6.2 Hz, 1H), 4.75 (q, J = 5.0 Hz, 1H), 4.63 (dd, J = 17.6, 6.6 Hz, 2H), 4.42 (dd, J = 10.7, 8.7 Hz, 1H), 4.27 (dt, J = 15.9, 7.6 Hz, 2H), 4.16 (dd, J = 10.3, 4.8 Hz, 1H), 3.92 – 3.84 (m, 2H), 3.65 (s, 6H), 3.55 (q, J = 9.8 Hz, 2H), 3.47 (dd, J = 14.1, 5.4 Hz, 1H), 3.26 (dd, J = 9.0, 7.6 Hz, 2H), 3.21 (t, J = 6.9 Hz, 2H), 3.11 (t, J = 6.7 Hz, 2H), 2.98 – 2.88 (m, 3H), 2.22 (t, J = 7.4 Hz, 2H), 1.83 (s, 1H), 1.76 – 1.56 (m, 6H), 1.50 (p, J = 7.0 Hz, 2H), 1.33 – 1.30 (m, 6H), 1.28 (s, 2H). ^{13}C NMR (151 MHz, MeOD-d_4) δ 176.18, 175.17, 170.38, 170.36, 169.93, 155.52, 152.17, 148.63, 147.07, 138.17, 135.78, 135.56, 132.40, 129.03, 128.21, 128.00, 127.97, 127.96, 127.94, 127.83, 109.84, 109.53, 107.58, 101.95, 101.56, 99.38, 80.32, 78.10, 77.89, 77.67, 74.46, 73.08, 72.67, 68.42, 67.78, 67.01, 66.43, 66.15, 55.27, 48.96, 43.85, 41.95, 40.83, 40.46, 37.87, 35.62, 35.21, 30.45, 29.40, 29.35, 29.11, 28.37, 25.84, 25.29, 19.27. HRMS (ESI) m/z caled for: $C_{63}H_{76}N_8O_{23}H$ [M+H] $^+$:1313.5096, found 1313.5099.

Synthesis of compound ETP-CM-RGD: Compound **7** (170 mg, 0.13 mmol, 1 eq) was solubilized in methanol (10 mL), and 10% palladium on carbon (200 mg) was included. The reaction mixture was purged with hydrogen and stirred under a hydrogen atmosphere at ambient temperature for 12 h. Upon completion, the mixture was filtered to exclude Pd/C, and the filtrate was concentrated under reduced pressure at low temperature to get the crude product. The residue was successively washed three times with methyl tert-butyl ether (10 mL) and CH_2Cl_2 (10 mL), yielding ETP-CM-RGD as a white solid (84.5 mg, 60% yield). ^{1}H NMR (600 MHz, MeOD-d_4) δ 7.00 (s, 1H), 6.51 (s, 1H), 6.34 (s, 2H), 5.96 (d, J = 4.6 Hz, 2H), 5.03 (d, J = 3.3 Hz, 1H), 4.77 (t, J = 5.1 Hz, 2H), 4.64 (dd, J = 15.5, 6.6 Hz, 2H), 4.47 – 4.42 (m, 2H), 4.36 (dd, J = 7.9, 5.5 Hz, 1H), 4.28 (t, J = 8.1 Hz, 1H), 4.16 (dd, J = 10.3, 4.9 Hz, 1H), 4.05 (d, J = 16.9 Hz, 1H), 3.77 (d, J = 16.9 Hz, 1H), 3.67 (s, 6H), 3.56 (dt, J = 14.0, 9.5 Hz, 2H), 3.49 (dd, J = 14.2, 5.5 Hz, 1H), 3.28 – 3.25 (m, 2H), 3.25 – 3.19 (m, 2H), 3.13 (t, J = 7.1 Hz, 2H), 3.00 – 2.93 (m, 1H), 2.86 (dd, J = 16.3, 4.9 Hz, 1H), 2.67 – 2.62 (m, 1H), 2.28 (t, J = 7.5 Hz, 2H), 1.99 (ddd, J = 10.8, 8.1, 5.4 Hz, 1H), 1.80 (dd, J = 9.6, 4.0 Hz, 1H), 1.68 (dd, J = 9.9, 5.5 Hz, 2H), 1.64 (ddd, J = 14.4, 7.3, 3.6 Hz, 3H), 1.53 (t, J = 7.0 Hz, 2H), 1.42 – 1.29 (m, 12H). ^{13}C NMR (151 MHz, MeOD-d_4) δ 176.81, 176.24, 176.16, 175.01, 173.26, 169.35, 157.33, 155.55, 152.18, 148.64, 147.08, 138.18, 132.41, 129.01, 127.99, 109.84, 109.53, 107.54, 101.91, 101.57, 80.32, 74.47, 73.10, 72.63, 68.42, 67.77, 66.15, 55.35, 55.25, 53.22, 50.85, 43.85, 42.25, 40.80, 40.61, 40.53, 38.25, 37.90, 35.30, 30.43, 29.45, 29.38, 29.18, 28.47, 25.97, 25.38, 24.78, 19.25. HRMS (ESI) m/z caled for: $C_{49}H_{65}N_7O_{21}H$[M+H]$^+$: 1088.4307, found 1088.4310.

Synthesis of compound 8: A combination of 4-(bromomethyl) benzoic acid (6.00 g, 29.8 mmol, 1.0 equivalent) and 2,3,3-trimethylindole (4.40 g, 30.0 mmol, 1.0 equivalent) in acetonitrile (40 mL) was subjected to reflux at 80 °C for 8 h. After completion, the reaction mixture was cooled to ambient temperature, and 200 mL of ethyl acetate was added to facilitate precipitation. The resultant product was obtained via filtration, further washed with ethyl acetate (3 × 50 mL) and methyl tert-butyl ether (3 × 50 mL), and dried under reduced pressure, yielding compound **8** as a pale pink solid (5.54 g, 50% yield). ^{1}H NMR (600 MHz, DMSO-d_6) δ 7.97 (d, J = 7.9 Hz, 2H), 7.90 (d, J = 7.5 Hz, 1H), 7.80 (d, J = 8.2 Hz, 1H), 7.61 (t, J = 7.5 Hz, 1H), 7.55 (d, J = 7.7 Hz, 3H), 5.97 (s, 2H), 3.01 (s, 3H), 1.63 (s,

6H). ^{13}C NMR (151 MHz, DMSO-*d*$_6$) δ 199.36, 167.21, 142.38, 141.54, 137.35, 131.46, 130.58, 130.07, 129.48, 128.07, 124.17, 116.32, 55.08, 50.90, 22.71, 15.24. HRMS (ESI) m/z caled for: $C_{19}H_{20}NO_2^+$ $[M]^+$: 294.1488, found 294.1491.

Synthesis of compound 9: A combination of compound **8** (2.00 g, 6.80 mmol, 1.0 equivalent) and 4-(1-pyrrolidinyl) benzaldehyde (1.20 g, 6.80 mmol, 1.0 equivalent) in anhydrous ethanol (30 mL) was subjected to reflux at 80 °C for 8 h. The reaction process was assessed by TLC (CH_2Cl_2/MeOH = 15:1), revealing the total consumption of compound **8**. The solvent was eliminated at decreased pressure, and the residue was refined using silica gel column chromatography (DCM/MeOH = 15:1), yielding compound **9** as a purple solid (1.54 g, 50% yield). ^{1}H NMR (600 MHz, MeOD-*d*$_4$) δ 8.33 (d, *J* = 15.2 Hz, 1H), 8.01 (d, *J* = 8.4 Hz, 2H), 7.84 (s, 2H), 7.67 (dd, *J* = 6.7, 1.5 Hz, 1H), 7.50 – 7.43 (m, 3H), 7.38 (d, *J* = 8.1 Hz, 2H), 7.14 (d, *J* = 15.3 Hz, 1H), 6.75 (d, *J* = 8.9 Hz, 2H), 5.75 (s, 2H), 3.57 – 3.50 (m, 4H), 2.09 (q, *J* = 3.4 Hz, 3H), 1.86 (s, 6H). ^{13}C NMR (151 MHz, MeOD-*d*$_4$) δ 179.63, 155.83, 153.49, 142.14, 141.63, 137.71, 130.18, 128.85, 127.25, 126.05, 122.64, 122.48, 112.74, 102.87, 50.94, 31.67, 30.42, 29.37, 29.07, 26.32, 24.85, 22.33, 13.04. HRMS (ESI) m/z caled for: $C_{30}H_{31}N_2O_2^+$ $[M]^+$: 451.2380, found 451.2381.

Synthesis of compound 10: Linoleic acid (1.00 g, 3.57 mmol, 1.0 eq) and 2,2′-dithiodiethanol (0.28 g, 1.78 mmol, 0.5 eq) were solubilized in dichloromethane (DCM, 20 mL), subsequently followed by the introduction of EDCI (1.36 g, 7.14 mmol, 2.0 eq) and DMAP (0.22 g, 1.78 mmol, 0.5 eq). The reaction mixture was agitated at ambient temperature for 12 h. Upon completion (monitored by TLC), the mixture was diluted with 20 mL of DCM and subsequently washed with 0.2 M aqueous HCl, water, and saturated brine in succession. The organic layer was dehydrated using anhydrous Na_2SO_4, subjected to filtration, and subsequently concentrated under reduced pressure. The residue was purified using silica gel column chromatography (PE/EA = 2:1), yielding compound **10** as a white liquid (0.60 g, 80% yield). ^{1}H NMR (600 MHz, $CDCl_3$) δ 5.42 – 5.30 (m, 4H), 4.35 (t, *J* = 6.7 Hz, 2H), 3.89 (t, *J* = 5.8 Hz, 2H), 2.93 (t, *J* = 6.7 Hz, 2H), 2.88 (t, *J* = 5.8 Hz, 2H), 2.77 (t, *J* = 6.9 Hz, 2H), 2.32 (t, *J* = 7.5 Hz, 2H), 2.25 (s, 1H), 2.05 (q, *J* = 7.1 Hz, 4H), 1.63 (t, *J* = 7.3 Hz, 2H), 1.35 (q, *J* = 7.1 Hz, 4H), 1.30 (dq, *J* = 9.4, 4.8 Hz, 10H), 0.89 (t, *J* = 6.8 Hz, 3H). ^{13}C NMR (151 MHz, $CDCl_3$) δ 173.17, 129.65, 129.46, 127.49, 127.33, 61.59, 59.65, 41.05, 36.48, 33.63, 30.95, 29.02, 28.77, 28.58, 28.52, 26.63, 26.62, 25.06, 24.31, 22.00, 13.51. HRMS (ESI) m/z caled for: $C_{22}H_{40}O_3S_2Na$ $[M+Na]^+$:439.2316, found 439.2319.

Synthesis of compound IAA-SS-LA: A combination of compound **10** (200 mg, 0.48 mmol, 1.0 equivalent) and compound **9** (216 mg, 0.48 mmol, 1.0 equivalent) in DCM (10 mL) was subjected to EDCI (183 mg, 0.96 mmol, 2.0 equivalents) and DMAP (30 mg, 0.24 mmol, 0.5 equivalents) and stirred at ambient temperature for 4 h. The course of the reaction was monitored by TLC. Following completion, the reaction mixture was diluted with 20 mL of DCM and subsequently washed in succession with 0.2 M aqueous HCl, water, and saturated brine. The organic layer was dehydrated using anhydrous Na_2SO_4, subsequently filtered, and concentrated under reduced pressure. The crude product was refined using silica gel column chromatography (CH_2Cl_2/MeOH = 30:1), yielding IAA-SS-LA as a dark purple solid (204 mg, 50% yield). ^{1}H NMR (600 MHz, MeOD-*d*$_4$) δ 8.34 (d, *J* = 15.1 Hz, 1H), 8.05 – 8.02 (m, 2H), 7.88 (s, 2H), 7.69 – 7.65 (m, 1H), 7.49 – 7.43 (m, 5H), 7.17 (dd, *J* = 15.2, 2.4 Hz, 1H), 6.71 (d, *J* = 8.8 Hz, 2H), 5.82 (s, 2H), 5.41 – 5.19 (m, 4H), 4.50 (t, *J* = 6.3 Hz, 2H), 4.26 (t, *J* = 6.2 Hz, 2H), 3.31 (p, *J* = 1.6 Hz, 1H), 3.03 (t, *J* = 6.4 Hz, 2H), 2.91 (dd, *J* = 7.2, 5.4 Hz, 2H), 2.73 (t, *J* = 6.7 Hz, 2H), 2.28 – 2.22 (m, 2H), 2.05 (p, *J* = 3.5 Hz, 4H), 2.01 (ddd, *J* = 9.4, 6.7, 2.3 Hz, 4H), 1.86 (s, 6H), 1.53 (dd, *J* = 9.8, 4.6 Hz, 3H), 1.30 – 1.22 (m, 17H), 0.88 – 0.85 (m, 3H). ^{13}C NMR (151 MHz, MeOD-*d*$_4$) δ 179.54, 173.60, 165.54, 155.89, 153.47, 142.13, 141.59, 139.60, 130.28,

129.93, 129.61, 129.51, 128.91, 127.76, 127.67, 127.28, 126.58, 122.76, 122.58, 112.69, 102.92, 62.76, 61.77, 50.94, 36.97, 36.75, 33.60, 31.29, 29.43, 29.40, 29.28, 29.11, 28.83, 28.78, 28.76, 28.74, 26.83, 26.78, 26.54, 26.52, 25.23, 24.87, 24.62, 22.27, 13.20, 13.18. HRMS (ESI) m/z caled for: $C_{52}H_{69}N_2O_4S^{2+}$ $[M]^+$: 849.4693, found 849.4697.

1.3. Combination drug treatment analysis

Cells (3,000/well, 100 μL) were cultured in 96-well plates for 12 h for attachment. ETP-CM-RGD and IAA-SS-LA were co-administered at predetermined molar ratios for 48 h. After treatment, 20 μL of MTT solution (5 mg/mL) was introduced to each well, followed by incubation at 37 °C for 2 h. Thereafter, the medium was removed, and 100 μL of DMSO was introduced to solubilize the formazan crystals. Absorbance (490 nm) was read on a FlexStation 3 microplate reader (Molecular Devices). Percentage growth inhibition across the concentration matrix was uploaded to SynergyFinder and evaluated by the HSA synergy model to generate synergy scores.

1.4. Preparation of E-R@ISSL

E-R@ISSL were synthesized using a conventional nanoprecipitation technique. ETP-CM-RGD (10.0 mg) and IAA-SS-LA (15.6 mg) were precisely measured (molar ratio 1:2) and fully dissolved in 100 μL DMSO to create a transparent organic precursor solution. The solution was subsequently introduced dropwise into 10 mL of ultrapure water at 30 °C under vigorous magnetic stirring. After complete addition, the suspension was agitated for an extra 30 min to facilitate self-assembly. The resultant nanoparticles were collected by centrifugation at 8000 rpm and subsequently freeze-dried to get the lyophilized E-R@ISSL powder for further use. ETP-CM-RGD NPs and IAA-SS-LA NPs were prepared utilizing an identical methodology for comparison. The hydrodynamic diameter, zeta potential, and polydispersity index (PDI) of the three nanoparticle formulations were assessed using dynamic light scattering (DLS, Zetasizer Nano-ZS, Malvern Instruments, UK), while their morphologies were examined via transmission electron microscopy (TEM, JEM-1400, JEOL, Japan).

To determine the encapsulation efficiency (EE) and drug loading (DL) of the two prodrug components in E-R@ISSL, the feeding amounts of ETP-CM-RGD and IAA-SS-LA were recorded as $W_{ETP\text{-}CM\text{-}RGD}$ and $W_{IAA\text{-}SS\text{-}LA}$. After centrifugation (10,000 × g, 10 min, 4 °C), the supernatant was filtered (0.22 μm) and analyzed by HPLC. Free ETP-CM-RGD and IAA-SS-LA in the filtrate were denoted as $W_{free\ ETP\text{-}CM\text{-}RGD}$ and $W_{free\ IAA\text{-}SS\text{-}LA}$, respectively. The precipitated fraction was dialyzed, freeze-dried, and weighed to obtain the mass of the dried nanoparticles (W_{NPs}), The EE and DL of ETP-CM-RGD and IAA-SS-LA were calculated according to the following equations:

$$\mathrm{EE}(\%) = \frac{W_{ETP-CM-RGD} - W_{free\ ETP-CM-RGD}}{W_{ETP-CM-RGD}} \times 100\%$$

$$\mathrm{DL}(\%) = \frac{W_{ETP-CM-RGD}}{W_{NPs}} \times 100\%$$

$$\mathrm{EE}(\%) = \frac{W_{IAA-SS-LA} - W_{free\ IAA-SS-LA}}{W_{IAA-SS-LA}} \times 100\%$$

$$\mathrm{DL}(\%) = \frac{W_{IAA-SS-LA} - W_{free\ IAA-SS-LA}}{W_{NPs}} \times 100\%$$

All studies were conducted in triplicate, and findings are expressed as mean ± SD.

HPLC conditions: Shimadzu SPD-16 system equipped with a C18 column (4.6 × 250 mm, 5 μm); mobile phase: methanol-water gradient: 0-5 min, 30-75% methanol; 5-10 min,75-95% methanol; 10-12 min, 95% methanol; 12-15 min, revert to 30% methanol; flow rate 1.0 mL·min^{-1}; detection wavelength 280 nm; injection volume 10 μL; column temperature 30 °C. Standard curve range: 0.03125-1 mg·mL^{-1}, confirmed for linearity and recovery.

1.5. Molecular dynamics simulation of nanoparticle assembly and spectroscopic evaluation

All-atom molecular dynamics (MD) simulations were conducted utilizing GROMACS 2020.7 to examine the

co-assembly behavior of ETP-CM-RGD and IAA-SS-LA. Molecular geometries were initially optimized with the Hartree-Fock method with the 6-31G* basis set in Gaussian 16. Merz-Kollman charges were computed to derive atomic electrostatic potentials.

The simulation system comprised ETP-CM-RGD and IAA-SS-LA in a 1:2 molar ratio, totaling 60 molecules randomly allocated within a cubic box of 12 × 12 × 12 nm^3. The system was solvated with SPC/E water and energy-minimized using the conjugate gradient method (maximum force 100 $kJ \cdot mol^{-1} \cdot nm^{-1}$, 10,000 iterations, step size 0.01 nm). Periodic boundary conditions were implemented in all directions, with long-range electrostatics addressed using the particle-mesh Ewald approach and van der Waals interactions cutoff at 1.0 nm.

Subsequent to energy minimization, the system experienced 1 ns of NPT equilibration (temperature 298.15 K, pressure 1 bar) with the V-rescale thermostat and Berendsen barostat, followed by 1 ns of NVT equilibration to achieve stabilization of temperature and volume. A 100 ns production MD simulation was subsequently performed under NPT conditions utilizing the Nosé-Hoover thermostat and Parrinello-Rahman barostat, with a 2 fs timestep and a 1.2 nm threshold for nonbonded interactions. All bonds involving hydrogen were constrained. Frames were saved every 20 ns (0–100 ns), visualized in VMD 1.9.3, and processed with built-in GROMACS analysis tools.

MD-predicted π–π stacking and hydrogen bonding were validated spectroscopically. UV-Vis spectra (MAPADA, UV-3100PC) of ETP, ETP-CM-RGD, IAA-SS-LA, and E-R@ISSL were recorded from 200 to 700 nm to detect shifts in aromatic absorption peaks. FTIR spectra (Thermo, Nicolet iN10) were obtained from KBr pellets of lyophilized samples over 4000 to 400 cm^{-1} to probe intermolecular hydrogen bonding. Shifts in the aromatic absorption bands provided further evidence of π–π stacking.

1.6. *In vitro* verification of CES2/GSH-responsive drug release mechanisms

E-R@ISSL nanoparticles equivalent to 5.5 mg of ETP-CM-RGD were dispersed in 10 mL PBS and incubated at 37 °C with gentle agitation to evaluate CES2/GSH dual-responsive release. Release media were configured as (i) PBS, (ii) 0.01 U/mL CES2, (iii) 10 mM GSH, and (iv) 0.01 U/mL CES2 + 10 mM GSH. At predetermined time points (0, 2, 4, 8, 12 h), 200 μL aliquots were withdrawn and centrifuged at 3,000 rpm for 10 min, and the supernatant was collected for analysis, and an equal volume of fresh buffer was added to maintain a constant total volume. ETP and IAA-SH concentrations were determined by HPLC using a C18 column (4.6 × 250 mm, 5 μm). The mobile phase (methanol/water) was run at 1.0 mL/min under the following gradient: 0-5 min, 30%-75% methanol; 5-10 min, 75%-95% methanol; 10-15 min, 95%-20% methanol. Detection was performed at 280 nm, with an injection volume of 10 μL and a column temperature of 30 °C. Quantification was accomplished utilizing standard calibration curves for ETP and IAA-SH (0.03125-1 mg/mL), which were confirmed for linearity and recovery. Supernatants from the 12 h incubation under the combined condition (0.01 U/mL CES2 + 10 mM GSH) were further analyzed by LC-MS (Thermo UPLC-ISQ EC) to identify major cleavage products. To evaluate the concentration-dependent responsiveness, release tests were performed in PBS containing varying concentrations of GSH (0, 5, and 10 mM) and CES2 activity (0.005 U/mL, 0.01 U/mL, and 0.02 U/mL). The cumulative release profiles were subsequently illustrated. All experiments were conducted in duplicate to guarantee repeatability.

1.7. Dynamic light scattering (DLS) analysis under stimulatory conditions and TEM observation after stimulation

To investigate the stimulus-induced disassembly behavior of E-R@ISSL, the nanoassemblies were incubated in PBS (pH 7.4), 10 mM GSH, 0.01 U CES2, or 10 mM GSH + 0.01 U CES2 at 37 °C. The final concentration of E-R@ISSL in each medium was adjusted to 0.5 mg/mL, and samples were incubated under gentle shaking (100 rpm). At 0, 2, 4, 8, and 12 h, aliquots were collected for DLS analysis to monitor changes in hydrodynamic size distribution. For morphological validation, the sample treated with the dual-stimulus condition (10 mM GSH + 0.01 U CES2) for

12 h was further subjected to TEM. Briefly, the sample was deposited onto a carbon-coated copper grid, negatively stained, air-dried, and imaged by TEM. The resulting morphology was compared with that of untreated nanoparticles to evaluate stimulus-induced structural disruption and disassembly.

1.8. Responsive release behavior under physiologically relevant conditions

To further evaluate the responsive release behavior of E-R@ISSL under more physiologically relevant conditions, release studies were carried out in PBS (pH 7.4), FBS, 10% mouse plasma/heparin in PBS, and tumor homogenate supernatant. E-R@ISSL was dispersed in each medium to a final concentration of 0.5 mg/mL and incubated at 37 ℃ under gentle shaking (100 rpm). At predetermined time points (0, 2, 4, 8, and 12 h), aliquots were withdrawn and immediately mixed with 3 volumes of ice-cold acetonitrile to terminate the reaction and precipitate proteins. After vortexing for 30 s, the samples were centrifuged at 12,000 × g for 10 min at 4 ℃, and the supernatants were collected for HPLC analysis. Released ETP and IAA-SH were quantified by HPLC using calibrated standard curves. For the preparation of tumor homogenate supernatant, freshly isolated NSCLC tumor tissues were homogenized in 3 volumes (w/v) of pre-cooled PBS buffer (10 mM, pH 7.4) on ice. The homogenates were first centrifuged at 5,000 × g for 10 min at 4 ℃ to remove large tissue debris, and if necessary, further centrifuged at 12,000 × g for 10 min at 4 ℃. The resulting supernatants were used for subsequent release studies. The results are presented as mean ± SD (n =3).

1.9. Evaluation of colloidal stability of E-R@ISSL

The long-term stability of E-R@ISSL was evaluated from both physicochemical and biological perspectives. For storage-related physicochemical stability, E-R@ISSL was dispersed in deionized water or PBS (pH 7.4) and stored at 4 ℃ for up to 21 days. At predetermined time points (0, 1, 3, 5, 7, 9, 11, 13, 15, 17, 19, and 21 days), aliquots were collected for measurement of hydrodynamic size and PDI by DLS. Zeta potential was measured in deionized water under low-ionic-strength conditions. The retention of ETP-CM-RGD and IAA-SS-LA in water and PBS was further determined by HPLC after disruption of the nanoassembly with an appropriate organic solvent. For biological stability under protein-containing conditions, E-R@ISSL was incubated in FBS and 10% mouse plasma/heparin in PBS at 37 ℃, and the hydrodynamic size and PDI were monitored over time by DLS. Because direct zeta-potential measurements in FBS or plasma-containing media are strongly affected by high ionic strength, conductivity, and abundant background proteins/electrolytes, particle size and PDI were used as the primary indicators of colloidal stability in these biologically relevant media. All experiments were performed in triplicate, and the results are presented as mean ± SD.

1.10. Hemolysis assay of E-R@ISSL

Fresh mouse red blood cells (RBCs) were collected with heparin anticoagulation, isolated by centrifugation at 4000 rpm for 10 min, and washed three times with 10 mM PBS (pH 7.4). The obtained RBCs were resuspended in PBS (pH 7.4) at a density of 1.0×10^8 cells/mL. RBC suspensions were then incubated with E-R@ISSL at different concentrations (calculated based on the total prodrug content) at 37 °C for 2 h. PBS and distilled water were used as the negative and positive controls, respectively. Because E-R@ISSL exhibited background absorbance around 540 nm, an additional material blank group (E-R@ISSL + PBS, without RBCs) was included for each tested concentration to correct for background interference. After incubation, the samples were centrifuged at 4000 rpm for 10 min, and the absorbance of the supernatants was measured at 540 nm. The hemolysis rate was calculated according to the following equation:

$$Hemolysis\ rate(\%) = \frac{(A_{Sample} - A_{Blank}) - A_{PBS}}{A_{Triton} - A_{PBS}}$$

Where A_{sample} is the absorbance of the supernatant from the RBC+ E-R@ISSL group, A_{blank} is the absorbance of

the E-R@ISSL + PBS (without RBCs) group. A_{PBS} is the absorbance of the RBC + PBS negative control group and A_{Triton} is the absorbance of RBC + 0.1% Triton X-100 positive control group. All experiments were performed in triplicate, and the results are presented as mean ± SD.

1.11. Difference in protein expression between ITGAV and ITGB5

The protein expression levels of ITGAV and ITGB5 in lung adenocarcinoma (LUAD) and lung squamous cell carcinoma (LUSC) were investigated utilizing the Human Protein Atlas (HPA) database (https://www.proteinatlas.org). Relative protein expression data in tumor and normal lung tissues were obtained from the CPTAC (Clinical Proteomic Tumor Analysis Consortium)—Protein Relative Expression datasets. Differential expression was visualized using bar plots and box plots.

1.12. Immunohistochemical examination of ITGAV and ITGB5 in pulmonary carcinoma

The immunohistochemistry (IHC) expression of ITGAV and ITGB5 proteins in lung cancer tissues was examined through the HPA database, utilizing the LUNG CANCER—Protein expression dataset. Representative IHC images and staining intensity data were extracted. Protein expression was evaluated according to staining intensity, classified as strong, moderate, weak, or negative.

1.13. Detection of receptor expression

Flow cytometry (FCM) was employed to assess protein expression across the four cell lines. Cells (~1×10^7/tube) were fixed, permeabilized, and stained overnight with primary antibody, followed by species-matched anti-rabbit IgG secondary antibody. The geometric mean fluorescence intensity was normalized to isotype control IgG to quantify expression. For immunofluorescence imaging, cells on coverslips were fixed (4% PFA, 20 min), blocked (5% BSA/PBS, 1 h), and incubated with anti-αvβ5 primary antibody in PBS overnight. After PBS rinses, secondary antibody IgG (H+L) were applied for 1 h in darkness. Nuclei were stained with DAPI and coverslips mounted with anti-fade medium before confocal imaging (FV4000, Olympus, Japan).

1.14. Molecular docking

The structures of αvβ5 (PDB ID: 7S47) and the RGD peptide were obtained from the PubChem database in SDF format. Molecular docking was conducted with Molecular Operating Environment (MOE) version 2019.01.

1.15. Differential expression analysis of GSH-related genes

The differential expression of several glutathione (GSH)-related genes in lung adenocarcinoma (LUAD) and lung squamous cell carcinoma (LUSC) was examined utilizing the Bioinformatics Toolbox platform. Tumor and normal tissue specimens from the TCGA-LUAD/LUSC datasets were chosen for study. Raw RNA-seq data underwent normalization and log2 transformation.

1.16. GSH detection and analysis

Reduced glutathione (GSH) was measured with a GSH Colorimetric Assay Kit. Supernatants from centrifuged cell samples (4,500 × g, 10 min) were combined with Reagent III and Reagent II (100 μL each) in a 96-well plate, mixed at 300 rpm for 1 min, and incubated for 5 min before reading absorbance at 405 nm with a microplate reader (Molecular Devices, FlexStation 3, USA).

1.17. Cell viability assay

Cell viability was determined by MTT assay. Exponentially growing cells were seeded in 96-well plates (3,000 cells/well) and treated with compounds at the indicated concentrations for 48 h (n = 3). Viability was assessed by adding 20 μL MTT (5 mg/mL), incubating for 2 h at 37 °C, removing the medium, and dissolving the formazan product in 100 μL DMSO. Absorbance at 490 nm was measured on a microplate reader.

1.18. Colony formation assay

LLC and PC-9 cells in the logarithmic growth phase were seeded onto 6-well plates at a density of 500-1000

cells/well. Subsequent to cell attachment, the cells were subjected to various formulations, including ETP-CM-RGD (1.2 μM), E-R@ISSL (0.6 μM), and IAA-SS-LA (2.4 μM). After 48 h of drug exposure, the medium was replaced with fresh serum-free medium, and the cells were further cultivated for 10 to 14 days. The culture medium was refreshed every three days until discernible colonies formed in the control well. Thereafter, the medium was eliminated, and cells were washed twice with PBS, fixed in 4% PFA for 10 min, and stained with 0.1% crystal violet for 15 min at ambient temperature. After rinsing with PBS and air-drying, plates were imaged. Colonies containing more than 50 cells were scored as positive using ImageJ.

1.19. Cell morphology observation

LLC and PC-9 cells were seeded into 6-well plates and cultured overnight. After attachment, the cells were exposed to ETP-CM-RGD, E-R@ISSL, or IAA-SS-LA for 24-48 h, after which morphological changes were examined under an inverted optical microscope and photographed.

1.20. Cell proliferation assay

Cells plated in 12-well plates received the indicated treatments for 24 h and were then incubated with 10 μM EdU for 2 h. Proliferating cells were labeled with the BeyoClick™ EdU-647 kit. After a 2 h pulse with 10 μM EdU, cells were fixed in 4% PFA for 15 min and processed with Click Additive Solution. DAPI was applied for nuclear counterstaining, and images were captured on a confocal microscope.

1.21. Cell apoptosis assay

Cells grown in 6-well plates were exposed to ETP-CM-RGD, E-R@ISSL, or IAA-SS-LA for 48 h. Post treatment, both floating and attached cells were harvested, rinsed two times with cold PBS, and reconstituted in 195 μL binding buffer. After the addition of 5 μL Annexin V-FITC and 10 μL propidium iodide (PI), the samples were incubated for 15 min at ambient temperature in the dark before analysis on a BD FACSVerse flow cytometer (BD Biosciences, USA). Viable, early apoptotic, late apoptotic, and necrotic populations were quantified. Every experiment was conducted in triplicate.

1.22. Nuclear morphological changes

LLC and PC-9 cells were inoculated onto glass coverslips in 24-well plates and permitted to adhere overnight. Following attachment, the cells were subjected to various formulations (ETP-CM-RGD, E-R@ISSL, and IAA-SS-LA) for 48 h. Subsequent to treatment, the cells were rinsed twice with PBS, fixed in 4% PFA at ambient temperature for 15 min, then stained with DAPI in the dark for 5 min. The coverslips were subsequently mounted, and nuclear morphology was seen using a confocal microscope.

1.23. Immunofluorescence analysis of γ-H2AX

DNA double-strand breaks were assessed by γ-H2AX immunofluorescence. Treated cells on coverslips were fixed (4% PFA/PBS, 15 min), permeabilized (0.5% Triton X-100/PBS, 10 min), blocked (3% BSA/PBS, 1 h), and probed with anti-γ-H2AX antibody overnight at 4 °C. Alexa Fluor 488-conjugated secondary antibody (goat anti-rabbit IgG, 1:500; Molecular Probes) was applied for 30 min at room temperature. Confocal images were acquired on a confocal microscope.

1.24. Comet assay

DNA strand breaks were assessed by alkaline comet assay. Treated cells (24 h) were trypsinized, washed, and resuspended in PBS (2×10^4 cells/mL). The suspension (400 μL) was combined with low-melting-point agarose at 40 °C, layered onto slides, and gelled. Lysis proceeded for 8 h at 37 °C in neutral buffer under light-protected conditions. Washed slides were electrophoresed at 20 V for 25 min, stained with PI (2.5 μg/mL, 20 min), and imaged on an Olympus IX73 microscope. DNA migration was quantified as tail moment (tail DNA% × tail length) with Comet Assay IV software.

1.25. Analysis of human 8-hydroxydeoxyguanosine (8-OHdG)

8-OHdG concentrations were determined using a Human 8-hydroxydeoxyguanosine ELISA Kit. After 24 h treatment with ETP-CM-RGD, E-R@ISSL, or IAA-SS-LA, 100 μL of cell culture supernatant was collected and added to the pre-coated wells. HRP-labeled detection antigen (100 μL) was added, and the plate was incubated at 37 °C for 60 min. Following five washes, freshly prepared substrate solution (H_2O_2: 0.1% TMB = 1:1000 μL/well) was introduced per well and incubated for 15 min at 37 °C. The reaction was halted using 50 μL of stop solution, and absorbance at 450 nm was read on a microplate reader.

1.26. MitoTracker staining and confocal colocalization

LLC and PC-9 cells were seeded in confocal dishes and cultured for 12 h. The cells were subsequently exposed to IAA or E-R@ISSL for 2 h. Mitochondria were stained with MitoTracker™ Deep Red FM for 15 min following medication removal. The dye solution was eliminated, and the cells were rinsed twice with PBS. Nuclei were counterstained with Hoechst 33342 for 5 min, followed by two additional PBS washes. Ultimately, pictures were obtained with a confocal laser scanning microscope.

1.27. Mitochondrial morphological changes

LLC and PC-9 cells were seeded in confocal dishes and cultured for 12 h. The cells were subsequently subjected to various formulations (ETP-CM-RGD, E-R@ISSL, and IAA-SS-LA) for 12 h. After drug removal, mitochondria were stained with MitoTracker™ Deep Red FM for 15 min. The dye solution was removed, and cells were rinsed twice with PBS. Nuclei were counterstained with Hoechst 33342 for 5 min, followed by two washes with PBS. Ultimately, pictures were obtained with a confocal laser scanning microscope.

1.28. Reactive oxygen species (ROS) assay

LLC and PC-9 cells (1×10^6 cells/well) were grown in 6-well plates and cultured overnight before 24 h drug exposure. For FCM, cells were incubated with 10 μM DCFH-DA in serum-free medium for 30 min at 37 °C in darkness, washed with PBS, and immediately analyzed. DCF fluorescence was recorded in the FITC channel. For fluorescence imaging, after medium removal, treated cells were directly stained with 10 μM DCFH-DA under the same conditions and imaged under a fluorescence microscope.

1.29. Determination of $NADP^+$/NADPH levels and the $NADP^+$/NADPH ratio

Subsequent to the specified treatments, cells were harvested by discarding the culture medium and washing twice with ice-cold PBS. Cells were lysed on ice with a NADP(H) extraction buffer, and lysates were clarified by centrifugation at 12,000 × g (10 min, 4 °C). The supernatants were utilized for future measurements. Total NADP(H) and NADPH levels were measured following the manufacturer's guidelines of the $NADP^+$/NADPH assay kit. The kit selectively distinguishes $NADP^+$ from NADPH through an enzymatic degradation step. Samples or standards were combined with the detection working reagent in 96-well plates, and absorbance/fluorescence was measured using a microplate reader. A standard curve was generated for quantification. $NADP^+$ levels were derived from the total NADP(H) and NADPH, or ascertained directly according to the kit protocol, after which the $NADP^+$/NADPH ratio was computed. Data were adjusted to total protein content (BCA test) or cell count and analyzed statistically.

1.30. Mitochondrial membrane potential (MMP) assay

LLC and PC-9 cells (1×10^6/well) were cultured in 6-well plates overnight. After 24 h exposure to ETP-CM-RGD, E-R@ISSL, or IAA-SS-LA, cells were collected, stained with JC-1 working solution (1 mL) at 37 °C for 20 min, and analyzed by FCM for MMP changes. For imaging, cells were directly stained with JC-1 following medium removal and photographed using LSCM.

1.31. ATP detection and analysis

LLC and PC-9 cells were treated with ETP-CM-RGD, E-R@ISSL, or IAA-SS-LA for 24 h, then rinsed with

PBS and lysed with lysis buffer (200 μL/well). Cell lysates underwent centrifugation at 12,000 × g for 5 min to pellet debris. ATP detection working solution (100 μL) was added and permitted to equilibrate at room temperature for 5 min to consume background ATP. Sample or ATP standard (20 μL) was then introduced and thoroughly mixed to quantify the relative light units (RLU).

1.32. Western blot analysis

Treated cells were washed with cold PBS and extracted in RIPA buffer containing protease inhibitors and PMSF. After centrifugation, proteins were separated by 10% SDS-PAGE and transferred onto PVDF membranes. After blocking (5% non-fat milk/TBST, 2 h), membranes were incubated with primary antibodies overnight at 4 °C. Subsequent to three TBST washing, HRP-conjugated secondary antibodies were applied for 2 h at room temperature. Protein bands were visualized using an ECL kit and quantified with ImageJ.

1.33. Tumor model

C57BL/6*J* mice (6-7 weeks old, 18-20g) were purchased from SPBF (Beijing) Biotechnology Co., Ltd. All animal experiments were approved by the Animal Research Ethics Committee of Chengdu University of Traditional Chinese Medicine and conducted in accordance with the relevant guidelines (Animal Use License No. SYXK (Chuan) 2025-0294). Mice were housed individually in SPF animal facilities of Chengdu University of Traditional Chinese Medicine Research Institute. To establish the tumor model, mice were inoculated with 1×10^6 LLC cells into the right flank. When tumor volumes reached approximately 70 mm^3, the mice were randomly assigned into experimental groups ($n = 6$).

1.34. *In vivo* imaging of mice

A syngeneic LLC tumor model was developed in C57BL/6*J* mice. Upon tumor volumes reaching around 80-150 mm^3, tumor-bearing mice were randomly allocated into groups and intravenously administered IAA or E-R@ISSL at similar dosages via the tail vein ($n \geq 3$). At 2, 4, 8, 12, and 24 h post-injection, mice were sedated with inhaled isoflurane and underwent whole-body fluorescence imaging utilizing an *in vivo* small-animal imaging system. All imaging parameters, including excitation/emission filters, exposure duration, and gain, were standardized between groups, and fluorescence signals inside the tumor area of interest (ROI) were measured. Following the final imaging time point (24 h), the mice were killed, and tumors were promptly removed for *ex vivo* fluorescence imaging to assess tumor accumulation and tissue distribution.

1.35. Pharmacokinetic analysis

Healthy mice ($n = 10$) were fasted for 12 h with free access to water and injected intravenously via the tail vein with IAA or E-R@ISSL (5 mg/kg). Blood was drawn from the retro-orbital plexus at 0.083, 0.25, 0.5, 1, 2, 4, 8, 12, and 24 h post-injection ($n = 6$ per time point), collected into anticoagulant tubes, and centrifuged (3,500 rpm, 20 min). Supernatant (200 μL) was diluted to 1.0 mL with PBS, and fluorescence was measured at 560–650 nm. Untreated mouse plasma provided the blank value. Drug concentrations were calculated from net fluorescence intensity using a calibration regression equation. Plasma concentration-time profiles were fitted to a two-compartment model with DAS 2.0 software to estimate pharmacokinetic parameters.

1.36. Hematoxylin and eosin (H&E) and immunohistochemistry

Tumor tissues and major organs were fixed in 4% PFA, paraffin-embedded, and sectioned at 4 μm thickness. The sections were dewaxed, rehydrated, stained with hematoxylin and eosin (H&E), dehydrated, and mounted with neutral resin. For Ki67 immunohistochemical analysis, tumor paraffin sections were incubated with anti-Ki67 antibodies. For TUNEL, γ-H2AX, Cyt C, and GPX4 immunohistochemical staining, tumor paraffin sections were washed with PBS, treated with proteinase K for 30 min, and then incubated with TUNEL reaction solution. Nuclei were counterstained with DAPI, and all section images were captured using a digital pathology slide scanning system

(Hamamatsu, S60, Japan).

1.37. Immunofluorescence analysis of HMGB1/CRT

LLC and PC-9 cells grown on coverslips in 12-well plates were treated with ETP-CM-RGD, E-R@ISSL, or IAA-SS-LA for 24 h, then fixed with 4% PFA in PBS (15 min), permeabilized with 0.5% Triton X-100 in PBS, and incubated overnight at 4 °C with the primary antibody HMGB1/CRT. After PBS washes, cells were stained with Alexa Fluor 647-conjugated goat anti-rabbit IgG secondary antibody for 1 h at room temperature in the dark. Fluorescence pictures were acquired via confocal microscopy.

1.38. Measurement of lipid peroxidation (MDA) levels

Malondialdehyde (MDA) levels in cellular or tissue samples were measured using a Lipid Peroxidation MDA Assay Kit. Cells were washed with PBS, collected, and homogenized in lysis buffer, then incubated on ice for 10 min. The lysates were centrifuged at 12,000 × g for 10 min at 4 °C, and the resultant supernatants were utilized for analysis. One hundred microliters of the sample or standard solution were thoroughly combined with 200 μL of the thiobarbituric acid working reagent and incubated in a boiling water bath at 100 °C for 15 min. The reaction mixtures were swiftly cooled on ice to ambient temperature and subsequently transferred to a 96-well plate. The absorbance at 532 nm was quantified with a microplate reader. MDA levels were determined using the standard curve and normalized to the protein concentration. All experiments were conducted in duplicate.

1.39. Immunofluorescence analysis of GPX4

The protocol for immunofluorescence detection of GPX4 mirrors that of HMGB1/CRT, with the addition of Fer-1 as required, while all other procedures remain consistent.

1.40. Confocal Imaging of lipid peroxidation using BODIPY 581/591 C11

Lipid peroxidation was evaluated with the Lipid Peroxidation Assay Kit. LLC and PC-9 cells were cultivated on coverslip-equipped 12-well plates and subjected to the indicated formulations (ETP-CM-RGD, E-R@ISSL, or IAA-SS-LA, with or without Fer-1) for 24 h. Upon completion of the therapy, the medium was removed, and cells were delicately rinsed twice with cold PBS. A 2 μM working solution of BODIPY 581/591 C11 was prepared as instructed. Then, 500 μL of the dye solution was added to each well, and cells were incubated at 37 °C for 30 min in darkness. After incubation, the dye solution was eliminated, and cells were rinsed threefold with PBS. Optional fixing was conducted with 4% PFA for 10 min, succeeded by washing with PBS. Coverslips were affixed to glass slides, and fluorescence pictures were acquired via a laser scanning confocal microscope. The oxidized variant of BODIPY 581/591 C11 was observed in the green channel (Ex/Em ≈ 488/510 nm), whereas the reduced variant was observed in the red channel (Ex/Em ≈ 561/590 nm). The imaging parameters remained same across all groups.

1.41. Measurement of lipoxygenase (LOX) activity

Lipoxygenase (LOX) activity in cellular or tissue samples was evaluated with a commercial Lipoxygenase Activity Assay Kit (Fluorometric Method) in accordance with the manufacturer's guidelines. Cells were washed with PBS, collected, and homogenized in lysis buffer on ice for 10 min. After centrifugation at 12,000 × g for 10 min at 4 °C, the supernatants were collected. Each supernatant was mixed with the fluorescent LOX substrate working solution to a final substrate concentration of 50 μM and incubated at 37 °C for 30 min in darkness. The reaction mixtures were then transferred to a black 96-well plate, and fluorescence was quantified using a microplate reader (Ex=500 nm, Em=536 nm). Blank and standard controls were included in each assay. LOX activity was determined by fluorescence intensity and normalized to protein concentration.

1.42. Flow cytometric analysis of intracellular ferrous iron (Fe^{2+})

Intracellular Fe^{2+} levels were measured using a Cell Ferrous Iron (Fe^{2+}) Fluorometric Assay Kit modified for FCM. Briefly, treated cells were washed, trypsinized, centrifuged (300 × g, 5 min), and reconstituted in the Fe^{2+} probe

working solution. After 30 min incubation at 37 °C in darkness with gentle agitation every 10 min, cells were rinsed twice with PBS and resuspended in 500 µL PBS. FITC fluorescence (Ex/Em = 488/520 nm) was recorded for ≥10,000 events per sample on a flow cytometer with standardized acquisition settings. Mean fluorescence intensity (MFI) values were extracted using FlowJo to quantify intracellular Fe^{2+} concentrations.

1.43. FCM analysis of apoptosis

Apoptosis was assessed utilizing an Annexin V-FITC/PI Apoptosis Detection Kit. Briefly, both adherent and floating cells from treated and control groups were collected, washed twice with PBS, and harvested using mild trypsinization without EDTA. After centrifugation at 300 × g for 5 min, cell pellets were washed once with cold PBS and resuspended in binding buffer at ~1 × 10^6 cells/mL. A 195 µL aliquot of this suspension was transferred to a flow tube, stained with 5 µL Annexin V-FITC and 10 µL PI, gently mixed, and incubated at room temperature in the dark for 10-15 min. Binding buffer (400 µL) was then added to each tube, and samples were analyzed immediately on a flow cytometer (FITC: Ex/Em = 488/530 nm; PI: Ex/Em = 535/617 nm). A minimum of 10,000 events were documented per sample with consistent gating across all groups. Data were analyzed with FlowJo to quantify early apoptotic (Annexin V^+/PI^-), late apoptotic (Annexin V^+/PI^+), and necrotic (Annexin V^-/PI^+) populations.

1.44. FCM analysis of tumor immune microenvironment

To evaluate the immunomodulatory effects of the therapies, immune cell populations in tumor tissues were analyzed by FCM. Tumor specimens were aseptically obtained from mice, chopped into small fragments, and digested in RPMI-DMEM medium supplemented with 1 mg/mL collagenase type IV and 0.1 mg/mL DNase I at 37 °C for 30 min with moderate agitation. The processed tissues were filtered using 70 µm cell strainers (BD Falcon) and rinsed twice with PBS supplemented with 2% FBS to get single-cell suspensions. To analyze tumor-infiltrating immune cells, single-cell suspensions were incubated with anti-mouse CD16/CD32 antibody (Fc block) for 10 min at 4 °C, followed by staining with fluorophore-conjugated monoclonal antibodies targeting CD45, CD11c, CD80, CD86, CD3, CD4, CD8, and CD49b (BioLegend or eBioscience). Mature dendritic cells (DCs) were characterized as $CD45^+$ $CD11c^+$ $CD80^+$ $CD86^+$ cells; $CD4^+$ and $CD8^+$ T cells were recognized as $CD45^+$ $CD3^+$ $CD4^+$ and $CD45^+$ $CD3^+$ $CD8^+$ populations, respectively; and natural killer (NK) cells were classified as $CD45^+$ $CD3^-$ $CD49b^+$ cells. For the examination of regulatory T cells (Tregs) in the tumor, single-cell suspensions were stained with surface antibodies targeting CD4 and CD3, followed by intracellular labeling for Foxp3 utilizing a fixation/permeabilization kit (eBioscience, USA) in accordance with the manufacturer's guidelines. Tregs are characterized as $CD4^+$ $CD3^+$ $Foxp3^+$ cells. Dead cells were eliminated utilizing a Fixable Viability Dye (eBioscience), and fluorescence compensation was executed employing single-stained and fluorescence-minus-one (FMO) controls. Data were obtained using a BD LSRFortessa flow cytometer and processed with FlowJo software (version 10.8, Tree Star Inc., USA). A minimum of 100,000 $CD45^+$ events were collected per sample to guarantee accurate quantification.

1.45. Creation of an orthotopic LLC-luc lung tumor model and *in vivo* bioluminescence imaging

LLC-luc cells (0.5 × 10^5 in 50 µL) were mixed with an equal volume of Matrigel (50 µL), drawn into a pre-chilled insulin syringe, and kept on ice until injection. Mice were sedated with a 1% sodium pentobarbital solution at a dosage of 5 mg/kg and immobilized. Hair in the left chest and axillary area was removed to completely expose the left thoracic wall. A ~1 cm transverse skin incision was made approximately 1 cm below the left anterior axillary line using ophthalmic scissors. The heart pulsation served as an anatomical reference, and the incision was carefully displaced horizontally to the left to locate the left lung, characterized as a white, solid tissue contrasting with the dark-red myocardium. Upon verifying the injection site, the cell/Matrigel mixture was administered into the left lung with the needle implanted to a depth of approximately 3 mm at a nearly perpendicular angle. Following the injection, the needle was maintained in position for 5-10 s to reduce backflow. A minimal quantity of gentamicin-infused saline

was subsequently administered to the wound, and the incision was secured with 1-2 sutures. Erythromycin ophthalmic ointment was subsequently administered to the incision site to promote wound healing and avert infection, thereby concluding the model installation. Mice were administered an intraperitoneal injection of D-luciferin (15 mg/mL, 200 µL each mouse) for bioluminescence imaging (BLI). After 5 min, the mice were sedated with isoflurane for approximately 5 min and positioned in the imaging chamber. Images were acquired with a 10 s exposure time. The same mice were imaged on postoperative days 5, 10, 15, 20, 25, and 30 to monitor tumor growth longitudinally using *in vivo* small-animal imaging system (Tanon, ABLX6, China).

1.46. Hematological assessment in murine models

At the experimental conclusion, complete blood samples (about 0.5-1.0 mL) were obtained from mice through the retro-orbital venous plexus utilizing EDTA-K2 anticoagulant vacuum tubes. After gentle inversion to mix, complete blood count (CBC) analysis was performed within 4 h of collection. Hematological parameters were quantitatively assessed with an automated hematology analyzer (BC-2800 Vet) in accordance with the manufacturer's guidelines. The parameters analyzed comprised white blood cell count (WBC), red blood cell count (RBC), hemoglobin concentration (HGB), hematocrit (HCT), mean corpuscular volume (MCV), mean corpuscular hemoglobin (MCH), mean corpuscular hemoglobin concentration (MCHC), platelet count (PLT), and a five-part differential of WBCs, which included both percentages and absolute counts of neutrophils, lymphocytes, monocytes, eosinophils, and basophils. All data are expressed as mean ± standard deviation (SD). Group comparisons were conducted using one-way analysis of variance (ANOVA), with $P < 0.05$ being statistically significant.

1.47. Statistical analysis

Statistical significance was determined by Student's t-test (two-group comparisons) or one-way analysis of variance (ANOVA, multiple-group comparisons). A P value less than 0.05 was considered significant. All analyses were performed in GraphPad Prism version 9.0.

2. Supplementary Figures

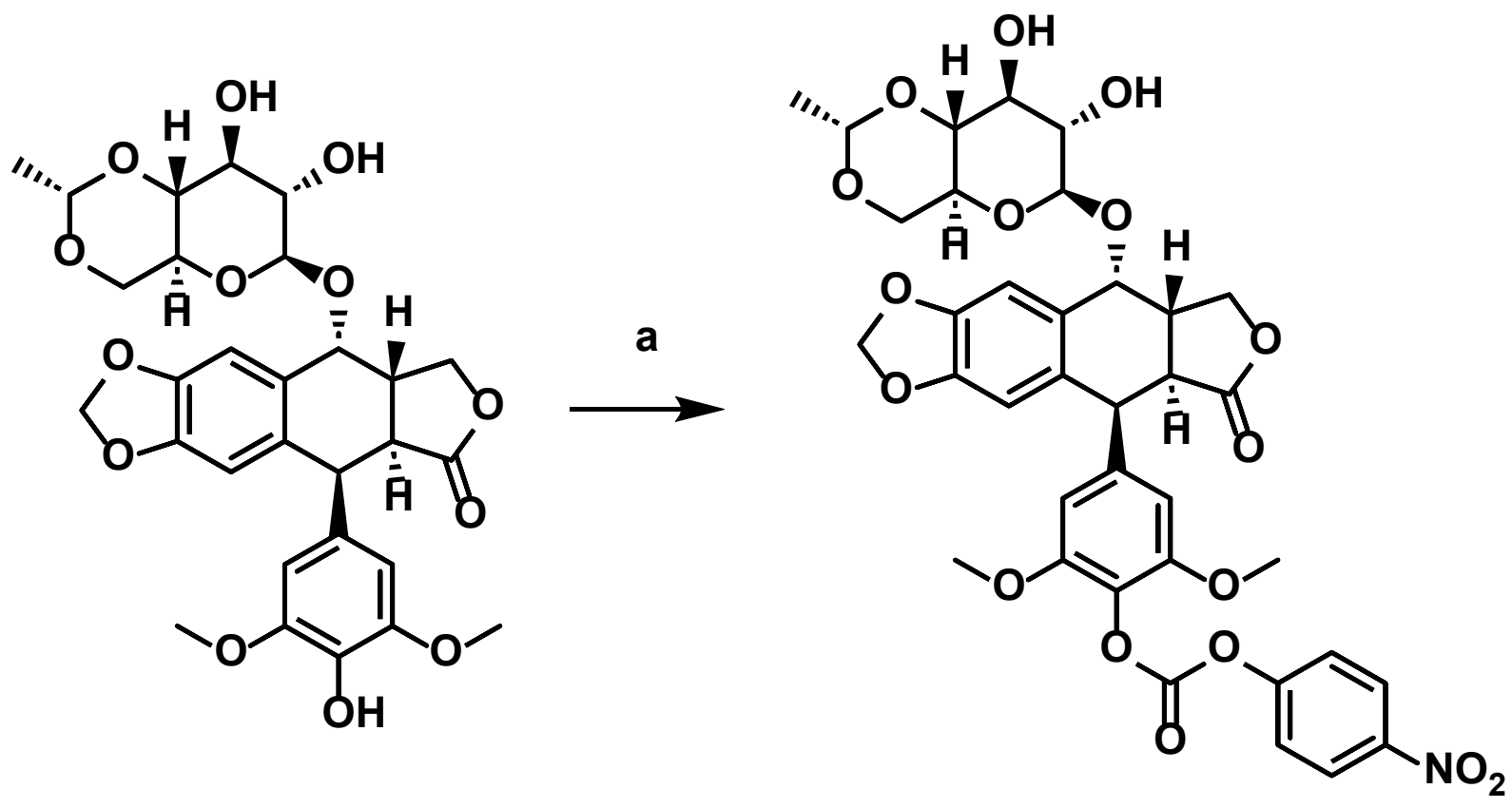

ETP ETP-PNP

Scheme S1. Reagents and conditions:(a) 4-Nitrophenyl chloroformate, Et_3N, THF, rt, 4 h.

Fig. S1. (A) Root mean square deviation (RMSD) profiles of E-R@ISSL throughout the molecular dynamics simulation. (B) The evolution of the radius of gyration (Rg) of E-R@ISSL during the simulation. (C) Variations in solvent-accessible surface area (SASA) of E-R@ISSL during the simulation duration. (D) UV vis absorption spectra of ETP, ETP-CM-RGD, IAA-SS-LA, and E-R@ISSL. (E) Comparison of FT-IR spectra for ETP, ETP-CM-RGD, IAA-SS-LA, and E-R@ISSL.

Fig. S2. (A) *In vitro* drug release of E-R@ISSL at pH=7.4. (B) *In vitro* drug release of E-R@ISSL in 0.01 U/mL CES2. (C) *In vitro* drug release of E-R@ISSL in 10 mM glutathione. (D) *In vitro* drug release of E-R@ISSL in 0.01 U/mL CES2 and 10 mM GSH. (E) *In vitro* cumulative release profiles of ETP from E-R@ISSL under various simulated physiological and tumor microenvironmental conditions: 0 U/mL CES2 with 0 mM GSH, 0.01 U/mL CES2 with 0 mM GSH, 0.02 U/mL CES2 with 0 mM GSH, and 0.01 U/mL CES2 with 10 mM GSH. (F) *In vitro* cumulative release profiles of IAA-SH from E-R@ISSL under various simulated physiological and tumor microenvironmental conditions: 0 U/mL CES2 with 0 mM GSH, 0 U/mL CES2 with 5 mM GSH, 0 U/mL CES2 with 10 mM GSH, and

0.01 U/mL CES2 with 10 mM GSH. (G) The mass spectrogram of ETP-CM-RGD. (H) The mass spectrogram of IAA-SS-LA. (I) The mass spectrogram of ETP. (J) The mass spectrogram of RGD-NH2. (K) The mass spectrogram of IAA-SH. (L) The mass spectrogram of LA-SH.

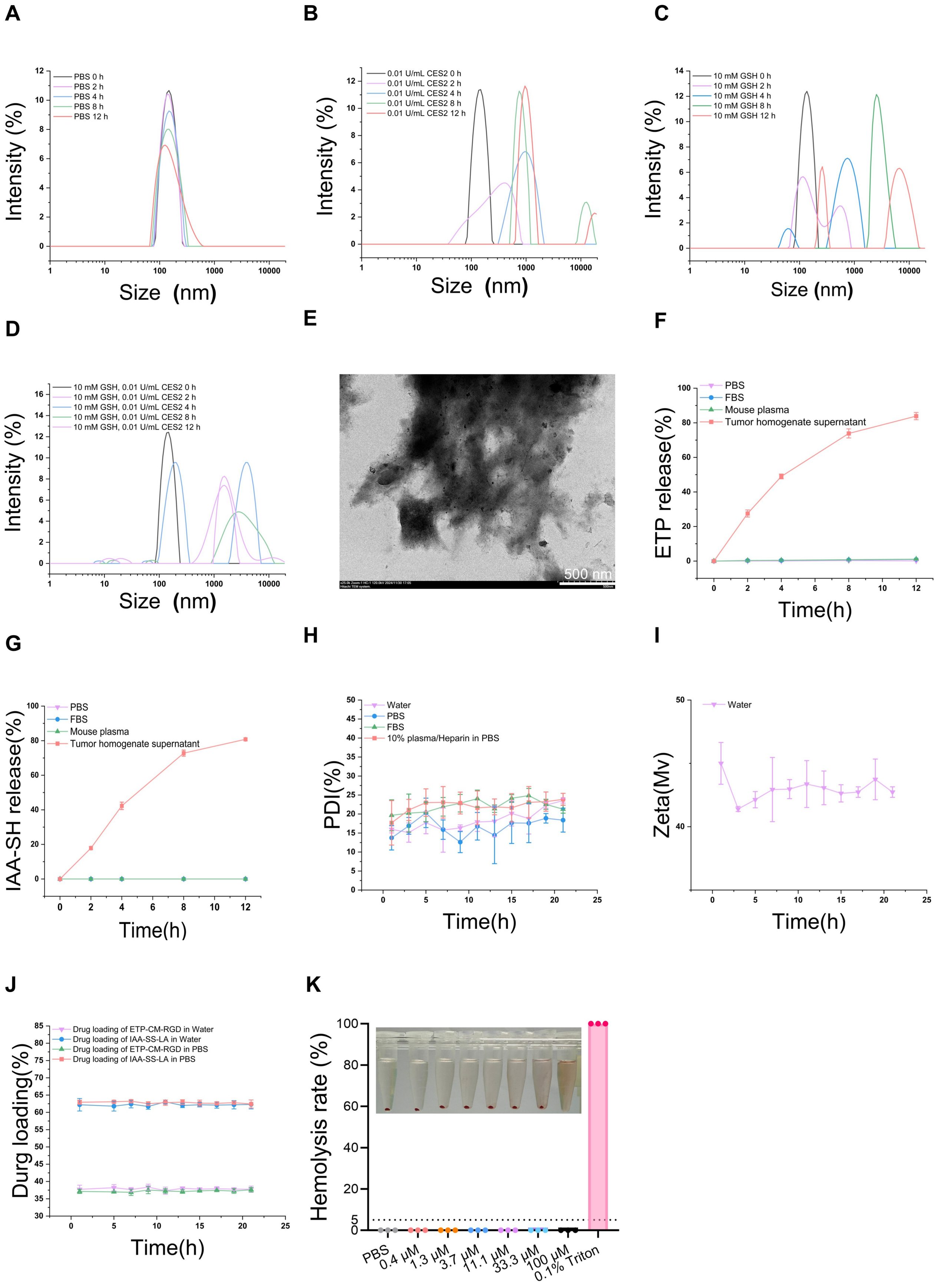

Fig. S3. (A) Hydrodynamic size distribution of E-R@ISSL incubated in PBS (pH 7.4) for 0, 2, 4, 8, and 12 h. (B)

Hydrodynamic size distribution of E-R@ISSL incubated with 10 mM GSH for 0, 2, 4, 8, and 12 h. (C) Hydrodynamic size distribution of E-R@ISSL incubated with 0.01 U/mL CES2 for 0, 2, 4, 8, and 12 h. (D) Hydrodynamic size distribution of E-R@ISSL incubated with 10 mM GSH and 0.01 U/mL CES2 for 0, 2, 4, 8, and 12 h. (E) Representative TEM image of E-R@ISSL after stimulation, showing the disruption of the original nanoparticle morphology and the formation of irregular flocculent aggregates, Scale bar = 500 nm. (F) ETP release profiles from E-R@ISSL in PBS, FBS, mouse plasma, and tumor homogenate supernatant. (G) IAA-SH release profiles from E-R@ISSL in PBS, FBS, mouse plasma, and tumor homogenate supernatant. (H) Time-dependent changes in the PDI of E-R@ISSL in deionized water, PBS, FBS, and 10% plasma/heparin in PBS. (I) Time-dependent changes in the zeta potential of E-R@ISSL in deionized water. (J) Drug-loading retention profiles of ETP-CM-RGD and IAA-SS-LA in deionized water and PBS. (K) Hemolysis rates of RBCs treated with different concentrations of E-R@ISSL and representative photographs of the corresponding samples. PBS and 0.1% Triton X-100 were used as the negative and positive controls, respectively.

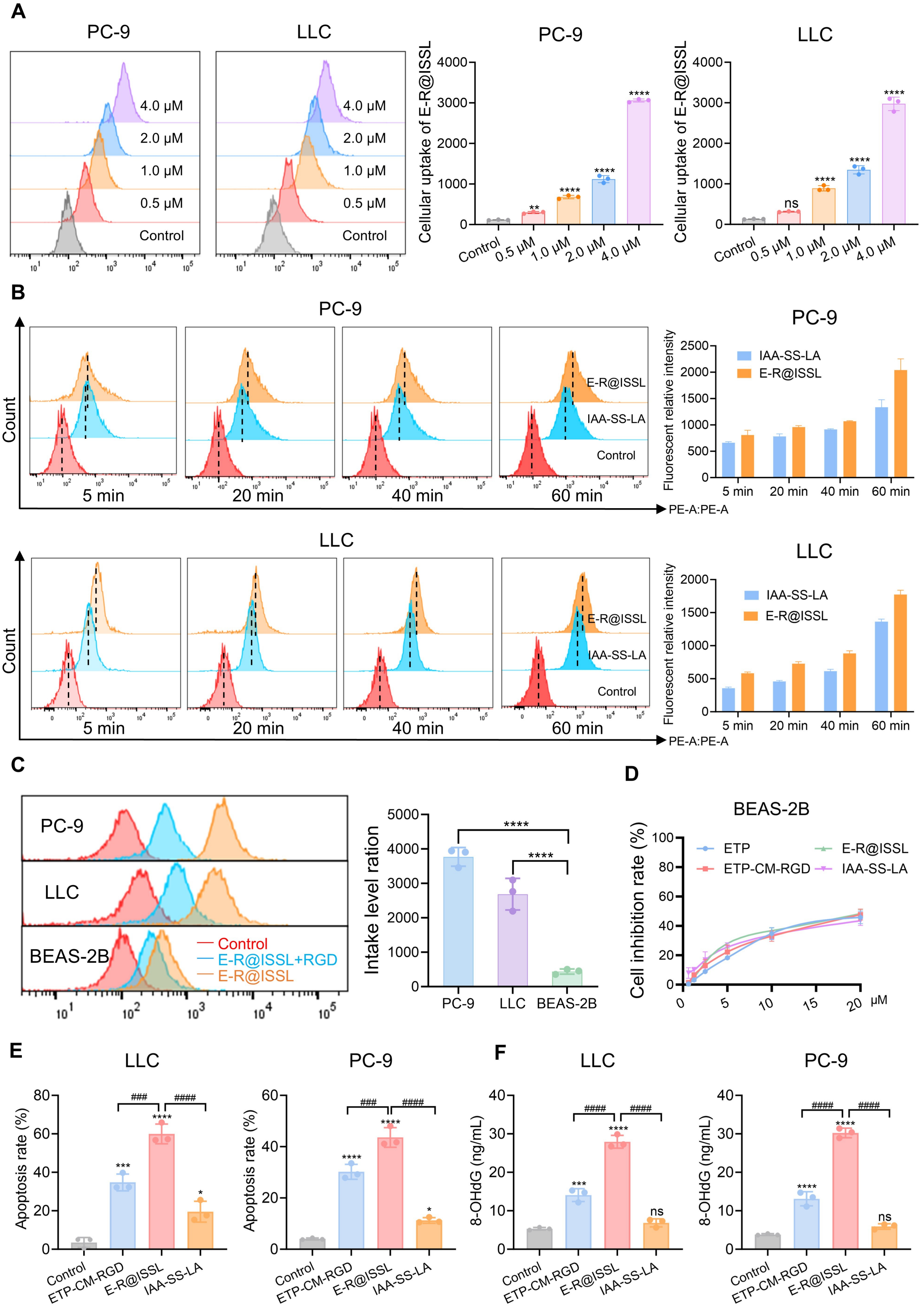
A
PC-9
LLC
4.0 μM
2.0 μM
1.0 μM
0.5 μM
Control
Cellular uptake of E-R@ISSL
Control
0.5 μM
1.0 μM
2.0 μM
4.0 μM
ns
B
Count
5 min
20 min
40 min
60 min
E-R@ISSL
IAA-SS-LA
Control
PE-A:PE-A
Fluorescent relative intensity
C
BEAS-2B
Control
E-R@ISSL+RGD
E-R@ISSL
Intake level ration
D
Cell inhibition rate (%)
ETP
ETP-CM-RGD
μM
E
Apoptosis rate (%)
F
8-OHdG (ng/mL)

Fig. S4. Cellular uptake, selective cytotoxicity, and DNA damage induced by E-R@ISSL in NSCLC cells. (A) FCM analysis showing the concentration-dependent cellular uptake of E-R@ISSL in LLC and PC-9 cells. (B) FCM analysis showing the time-dependent cellular uptake of E-R@ISSL and IAA-SS-LA in LLC and PC-9 cells. (C) RGD-mediated selective cellular uptake of E-R@ISSL in NSCLC cells. (D) Cytotoxicity evaluation of ETP, ETP-CM-RGD, E-R@ISSL, and IAA-SS-LA in BEAS-2B cells. (E) Quantitative evaluation of apoptosis in NSCLC cells following different drug treatments. (F) The 8-OHdG level variations in NSCLC cells following different drug treatments. ns, no substantial change, **$p < 0.01$, ***$p < 0.001$, ****$p < 0.0001$, vs. the control group. ##$p < 0.01$, ###$p < 0.001$, ####$p < 0.0001$, vs. the E-R@ISSL group.

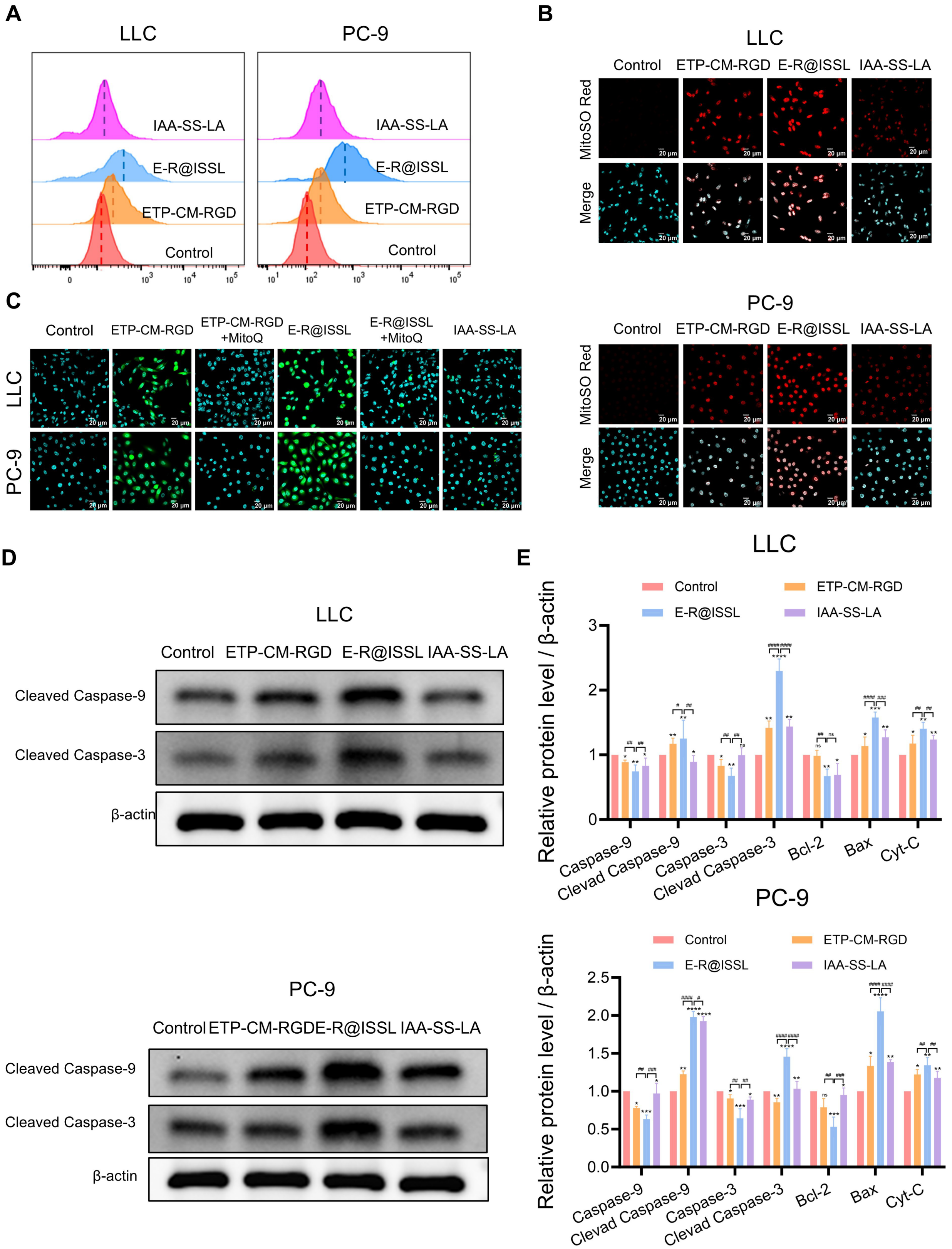

Fig. S5. Impacts of E-R@ISSL on mitochondrial ROS levels. (A) Dynamics of ROS levels in NSCLC cells induced by different treatments. (B) Mitochondrial superoxide fluorescence in LLC and PC-9 cells following different treatments. (C) Levels of ROS in NSCLC cells treated after MitoQ pretreatment. (D) Western blot analysis

of cleaved caspase-9 and cleaved caspase-3 expression in LLC and PC-9 cells following treatment with Control, ETP-CM-RGD, E-R@ISSL, and IAA-SS-LA. β-actin was used as the loading control. (E) Quantitative analysis of apoptosis-related protein expression in LLC and PC-9 cells after different treatments. Protein levels were normalized to β-actin. *$p < 0.05$, **$p < 0.01$, ***$p < 0.001$, ****$p < 0.0001$, vs. the control group. #$p < 0.05$, ##$p < 0.01$, ###$p < 0.001$, ####$p < 0.0001$, vs. the E-R@ISSL group.

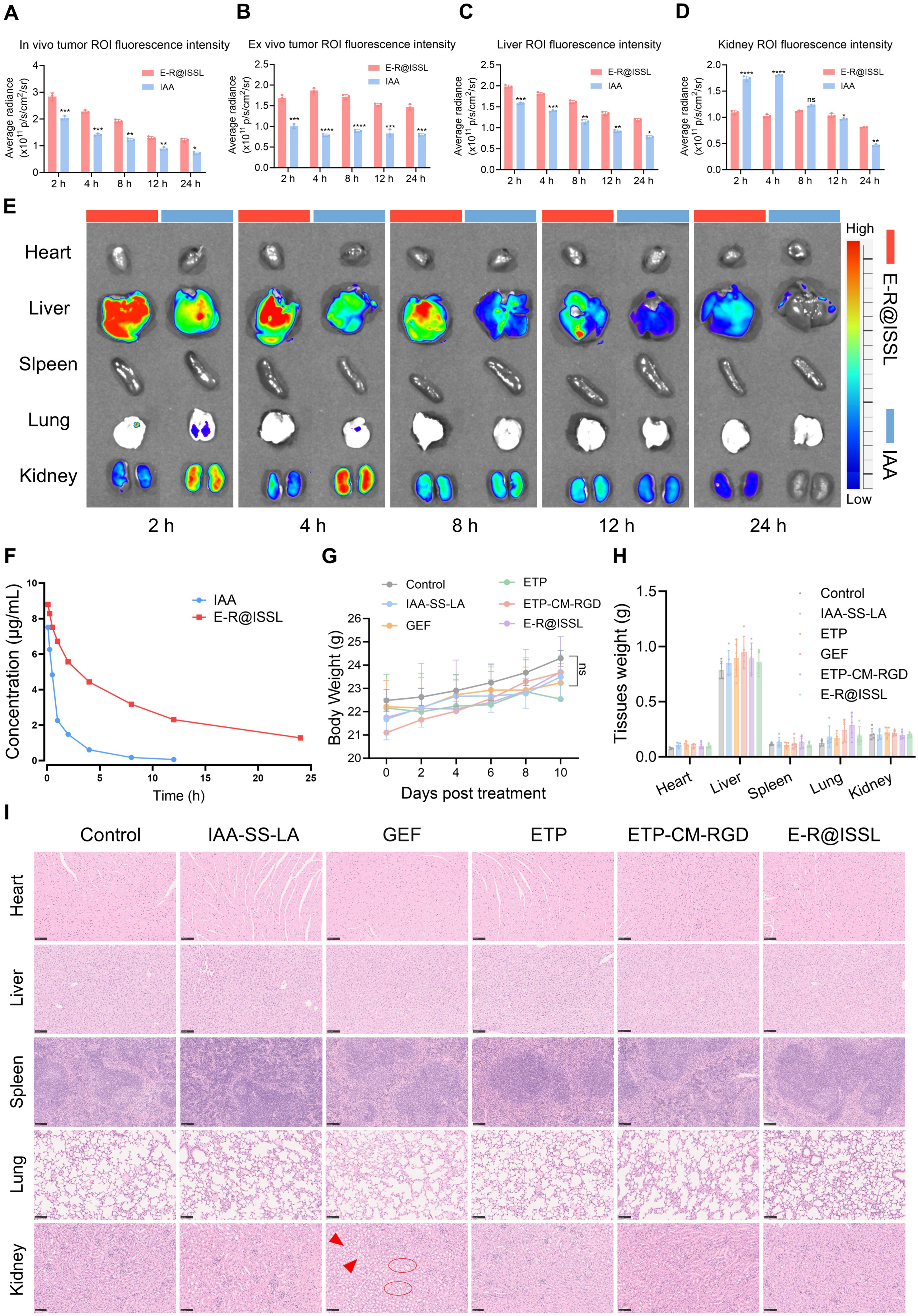
A
In vivo tumor ROI fluorescence intensity
B
Ex vivo tumor ROI fluorescence intensity
C
Liver ROI fluorescence intensity
D
Kidney ROI fluorescence intensity
E-R@ISSL
IAA
Average radiance
2 h
4 h
8 h
12 h
24 h
E
Heart
Liver
Slpeen
Lung
Kidney
High
Low
F
Concentration (μg/mL)
Time (h)
G
Control
IAA-SS-LA
GEF
ETP
ETP-CM-RGD
E-R@ISSL
Body Weight (g)
Days post treatment
ns
H
Tissues weight (g)
Heart
Liver
Spleen
Lung
Kidney
I
Control
IAA-SS-LA
GEF
ETP
ETP-CM-RGD
E-R@ISSL
Heart
Liver
Spleen
Lung
Kidney

Fig. S6. E-R@ISSL exhibits favorable biosafety in NSCLC. (A) Quantitative analysis of *in vivo* tumor ROI fluorescence intensity. (B) Quantitative analysis of *ex vivo* tumor ROI fluorescence intensity. (C) Quantitative analysis of liver ROI fluorescence intensity. (D) Quantitative analysis of kidney ROI fluorescence intensity. (E) *Ex vivo* fluorescence images of major organs and tumors at different time points after administration of E-R@ISSL or IAA. (F) Plasma concentration-time curves for IAA and E-R@ISSL. (G) Weight fluctuation trajectories for each cohort during the treatment duration. (H) The weights of heart, liver, spleen, lungs, and kidneys across various treatment groups. (I) H&E staining photos of major organs (heart, liver, spleen, lung, and kidney), scale bar = 100 µm. ns, no substantial change, *$p < 0.05$, **$p < 0.01$, ***$p < 0.001$, ****$p < 0.0001$.

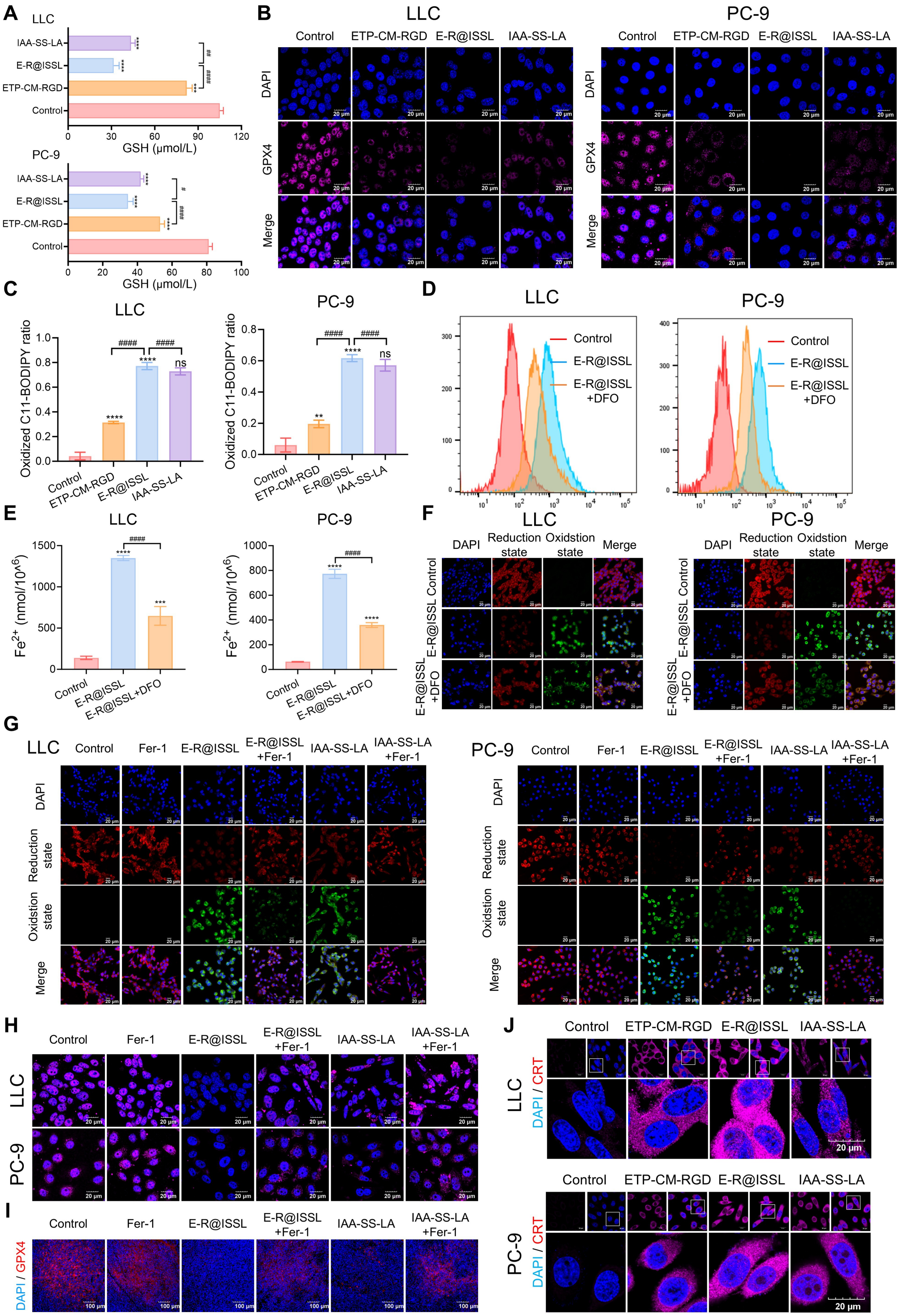
A
LLC
IAA-SS-LA
E-R@ISSL
ETP-CM-RGD
Control
GSH (μmol/L)
PC-9
B
LLC
PC-9
Control
ETP-CM-RGD
E-R@ISSL
IAA-SS-LA
DAPI
GPX4
Merge
20 μm
C
LLC
PC-9
Oxidized C11-BODIPY ratio
ns
D
LLC
PC-9
Control
E-R@ISSL
E-R@ISSL +DFO
E
LLC
PC-9
Fe2+ (nmol/10^6)
E-R@ISSL+DFO
F
LLC
PC-9
DAPI
Reduction state
Oxidstion state
Merge
G
LLC
PC-9
Fer-1
E-R@ISSL +Fer-1
IAA-SS-LA +Fer-1
H
I
DAPI / GPX4
100 μm
J
DAPI / CRT

Fig. S7. E-R@ISSL revealed mechanisms of inducing ferroptosis, activating immune cells, and enhancing antitumor immune responses. (A) Variations in GSH levels following treatment with various pharmaceuticals. (B) Immunofluorescence imaging of GPX4 alterations following various pharmacological interventions. (C) Quantitative analysis of the oxidized C11-BODIPY ratio in LLC and PC-9 cells after different treatments. (D) FCM analysis of intracellular Fe^{2+} levels with DFO treatment. (E) Quantitative analysis of intracellular Fe^{2+} levels with DFO treatment. (F) Fluorescence imaging of lipid peroxidation in LLC and PC-9 cells after E-R@ISSL treatment with or without DFO. (G) Immunofluorescence imaging of lipid peroxidation in Fer-1. (H) Immunofluorescence imaging of GPX4 alterations in NSCLC cells subjected to Fer-1 treatment. (I) Expression of GPX4 in tumor tissue following Fer-1 therapy. (J) Representative confocal microscopy pictures depicting CRT exposure following diverse pharmacological treatments of NSCLC cells. vs. Control: ns, no substantial change, ***p* < 0.01, ****p* < 0.001, *****p* < 0.0001; vs. E-R@ISSL: #*p* < 0.05, ##*p* < 0.01, ####*p* < 0.0001.

Fig. S8. E-R@ISSL enhances immunogenic cell death-associated ATP release and activates antitumor immune responses in tumor tissues. (A) ATP concentration in cellular supernatant. (B) FCM investigation of dendritic cell maturation ($CD80^+CD86^+$) in tumor tissues. (C) Representative FCM analysis illustrating the percentage of effector T cells ($CD4^+FCD8^+$) within tumor tissues. (D) Proportions of NK cells ($CD3^-CD49b^+$) within malignancies. (E) Representative FCM analysis illustrating the percentage of regulatory T cells (Tregs, $CD4^+Foxp3^+$) within tumor tissues. vs. Control: ns, no substantial change, ***p* < 0.01, ****p* < 0.001, *****p* < 0.0001; vs. E-R@ISSL: ##*p* < 0.01, ###*p* < 0.001, ####*p* < 0.0001.

Table S1 Compartmental pharmacokinetic parameters of E-R@ISSL and free IAA.

Compartmental parameters	Unit	E-R@ISSL	IAA
$t_{1/2}$	h	5.85	0.53
$AUC_{(0-t)}$	mg/L*h	72.867	10.552
$AUC_{(0-\infty)}$	mg/L*h	90.208	10.561
C_{max}	mg/L	8.27	7.51
T_{max}	h	0.250	0.083
$MRT_{(0-t)}$	h	8.148	2.136
$MRT_{(0-\infty)}$	h	13.85	2.42
CL	$L/h/m^2$	0.177	1.526

3 ^{1}H NMR (A) and ^{13}C NMR (B) of final compounds

A

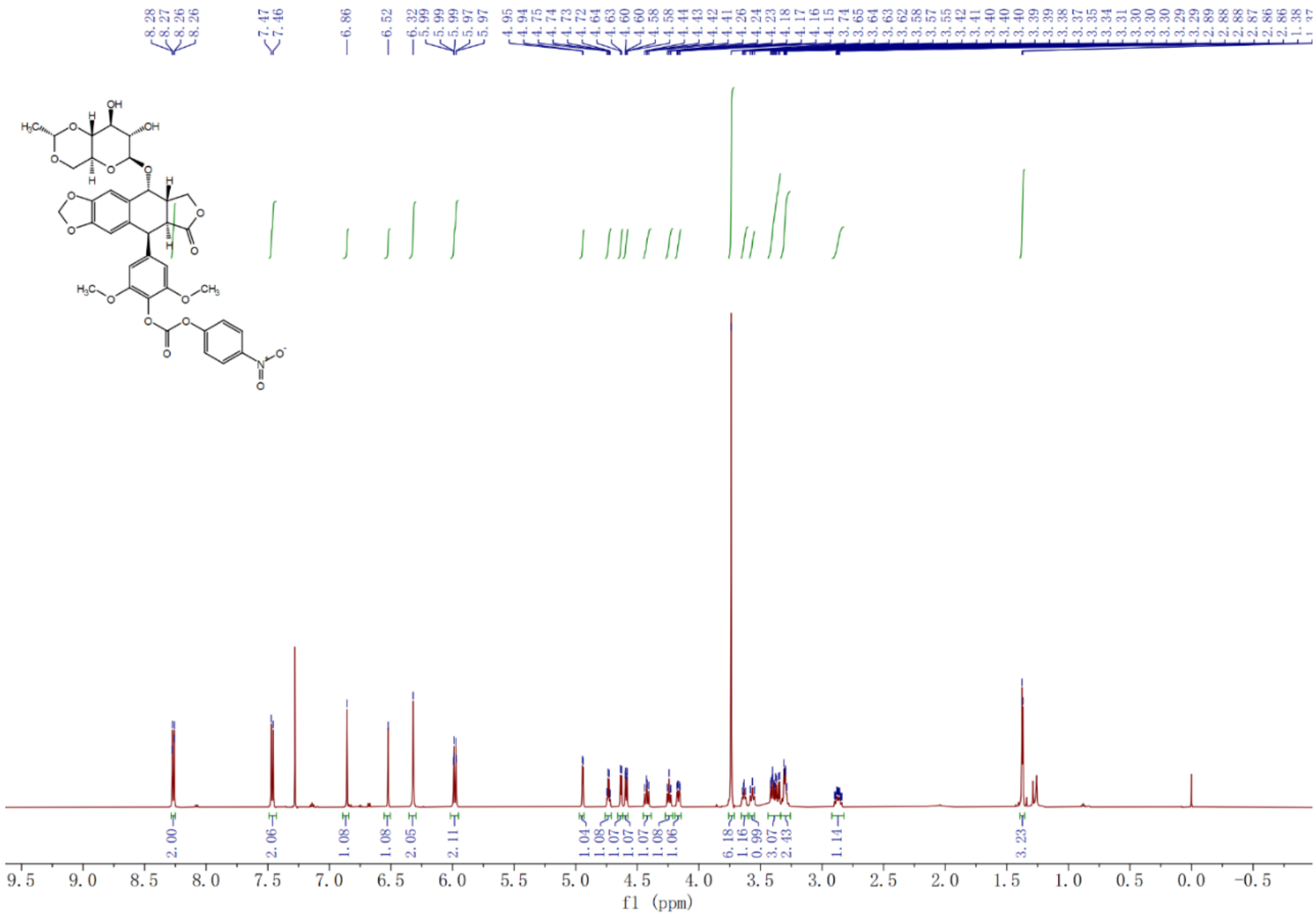

B

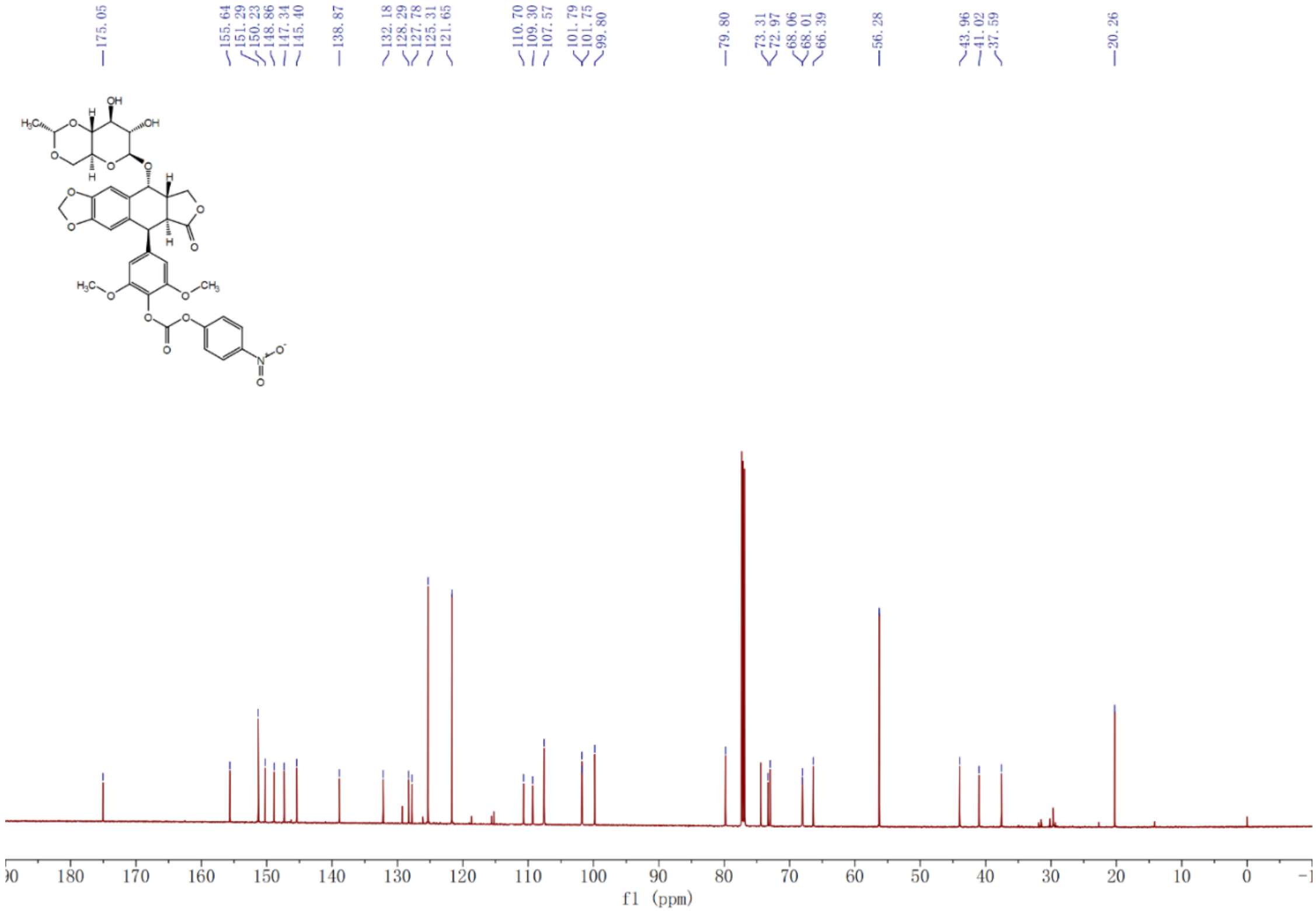

Fig. S9. ^{1}H-NMR (A) and ^{13}C-NMR (B) of ETP-PNP

A

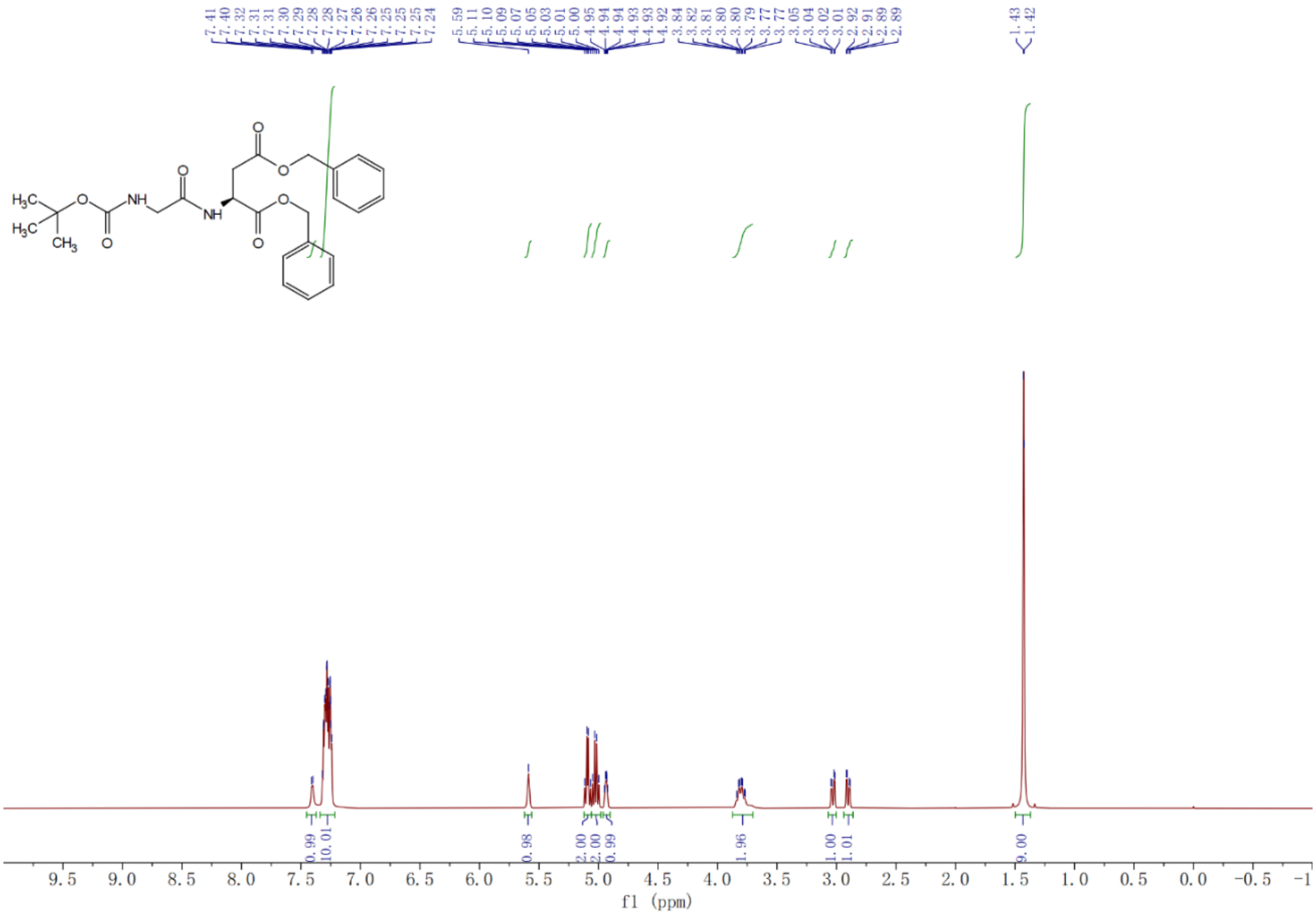

B

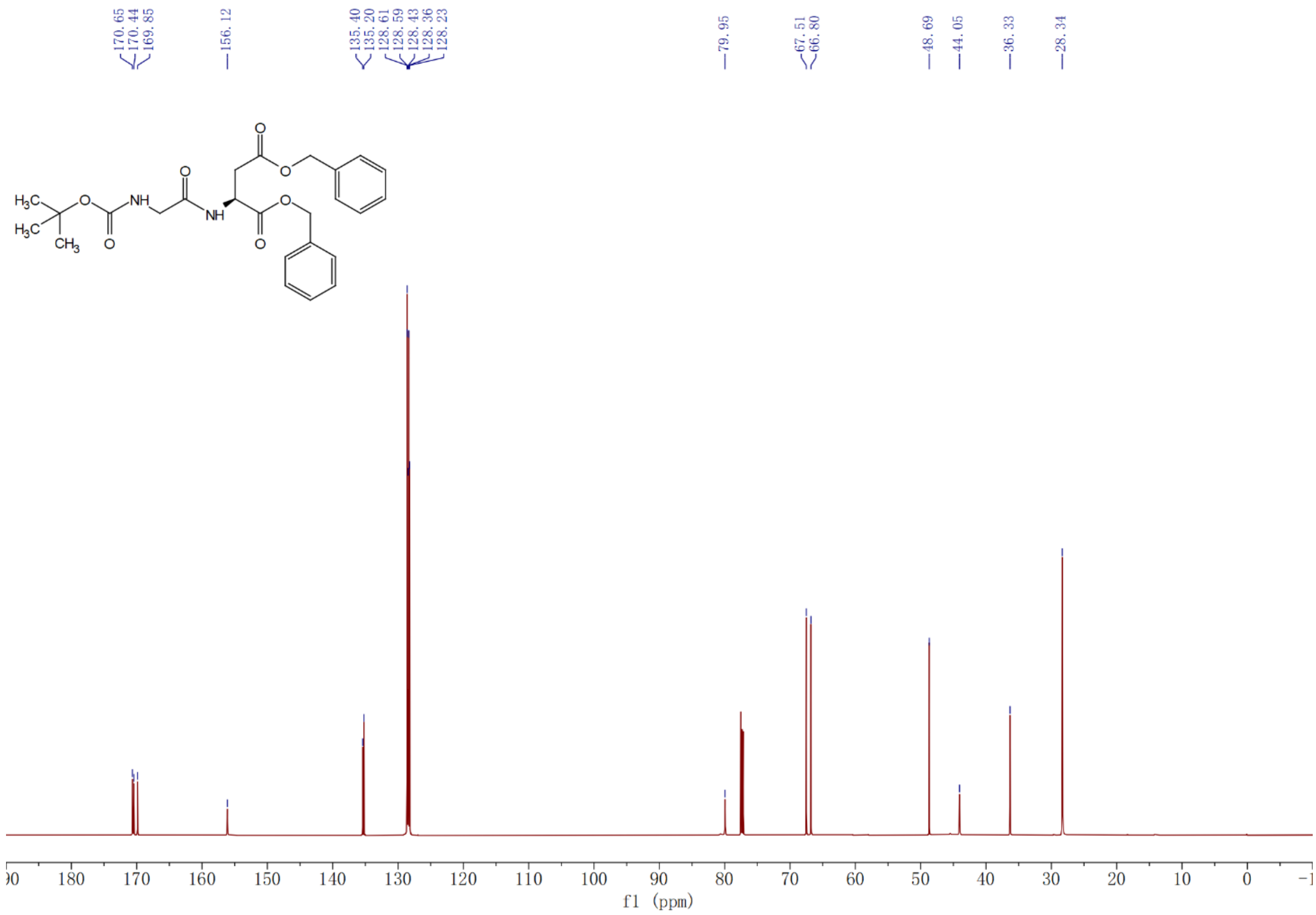

Fig. S10. ^{1}H-NMR (A) and ^{13}C-NMR (B) of Compound 1

A

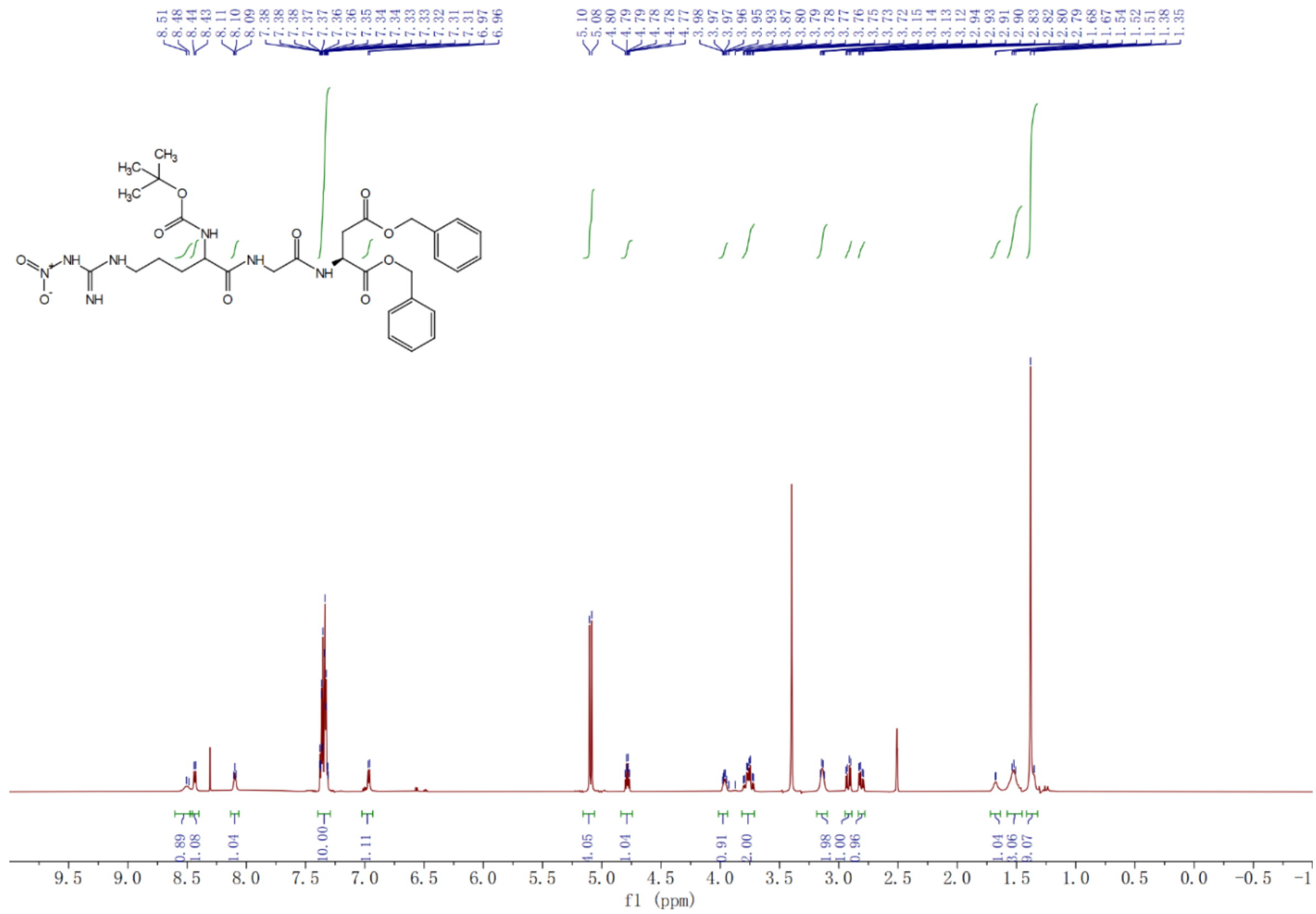

B

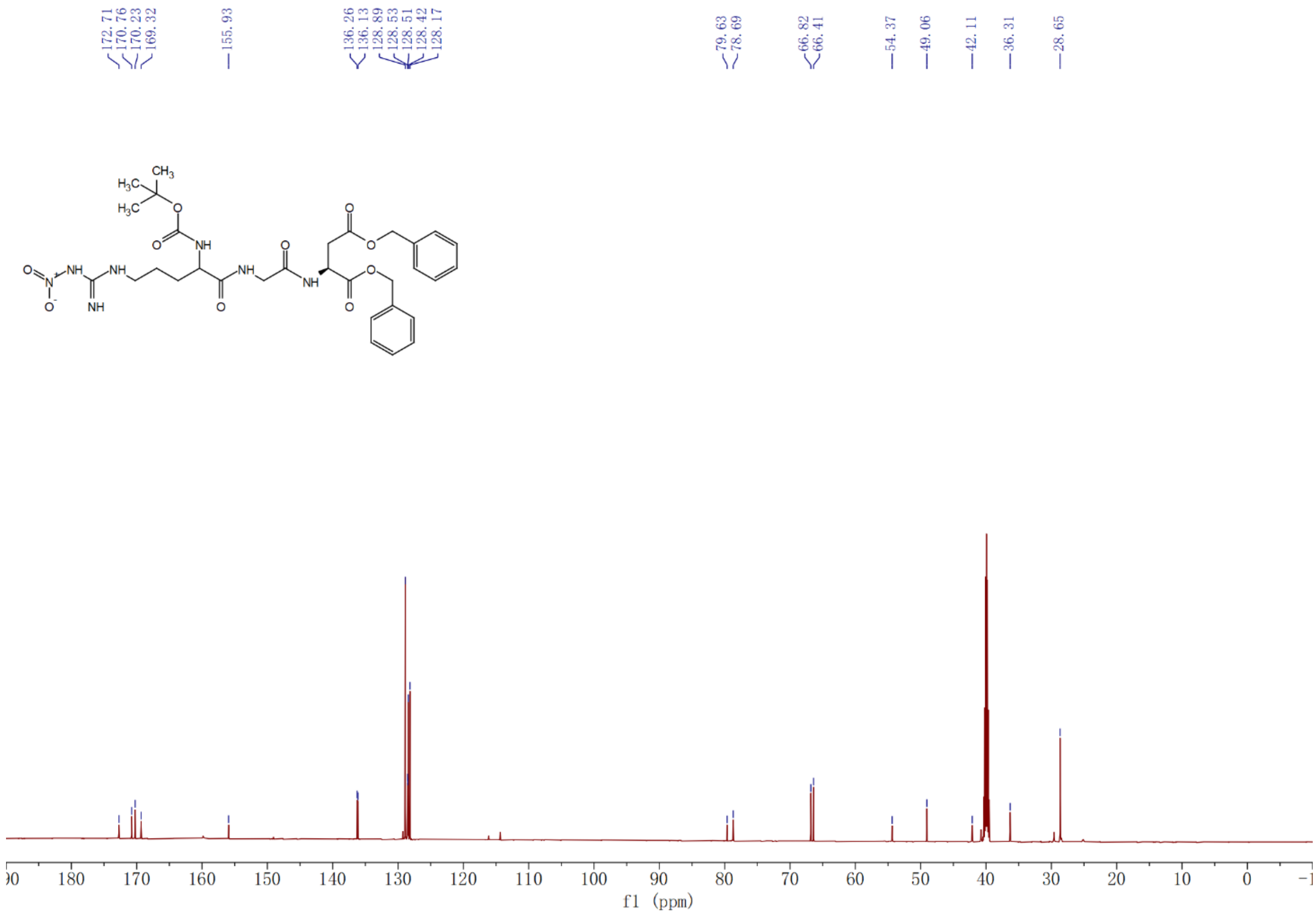

Fig. S11. ^{1}H-NMR (A) and ^{13}C-NMR (B) of Compound 3

A

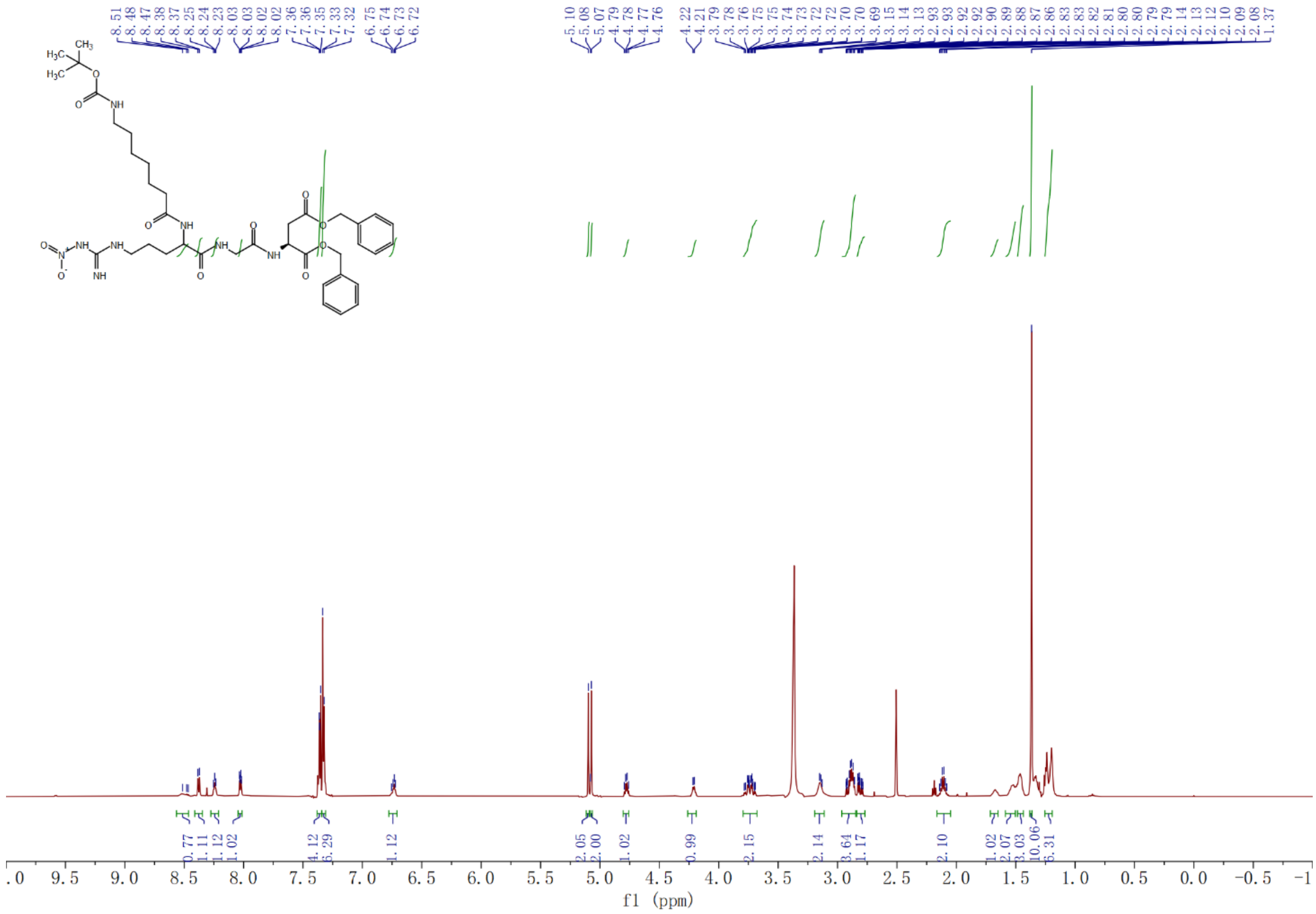

B

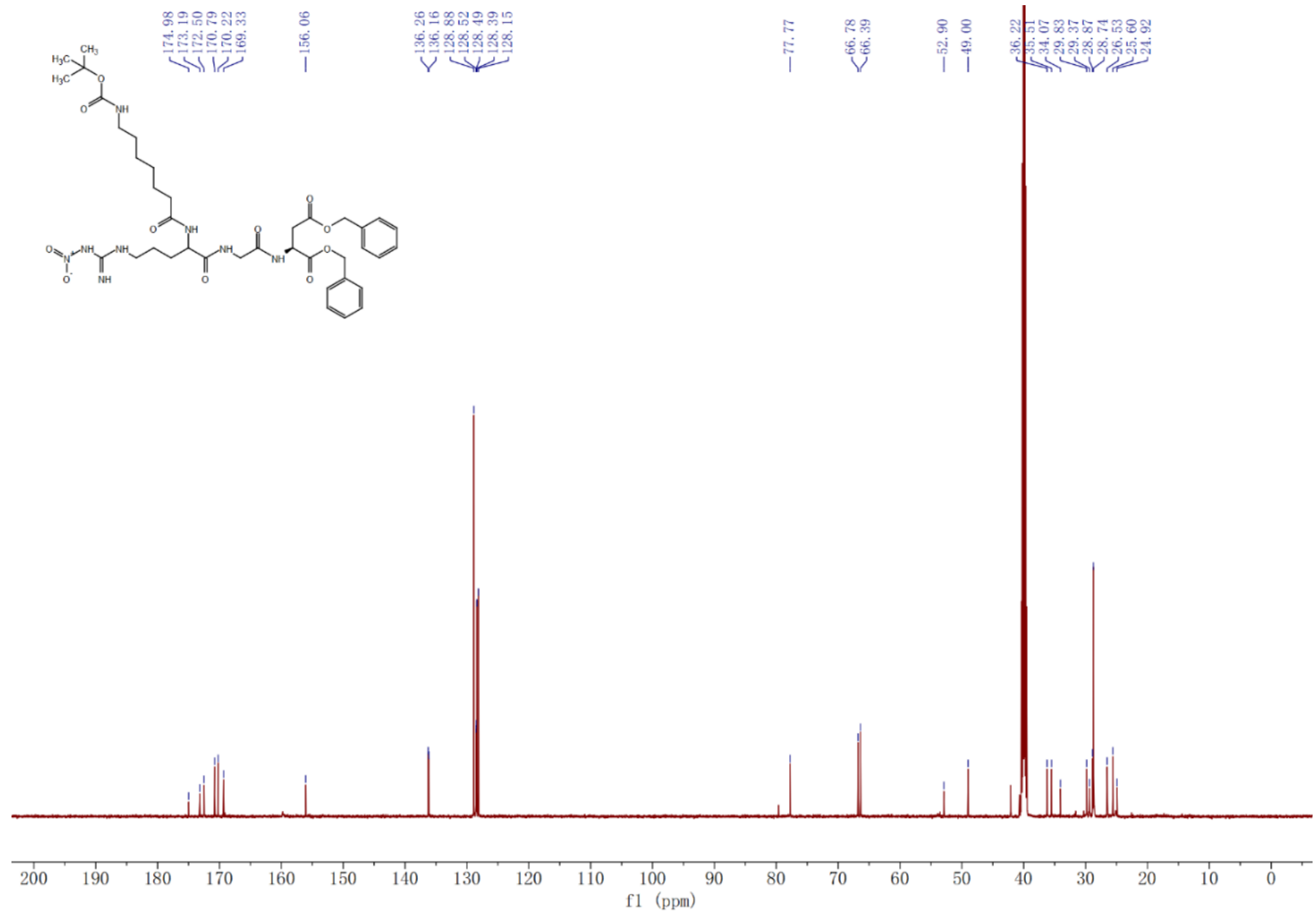

Fig. S12. ^{1}H-NMR (A) and ^{13}C-NMR (B) of Compound 5

A

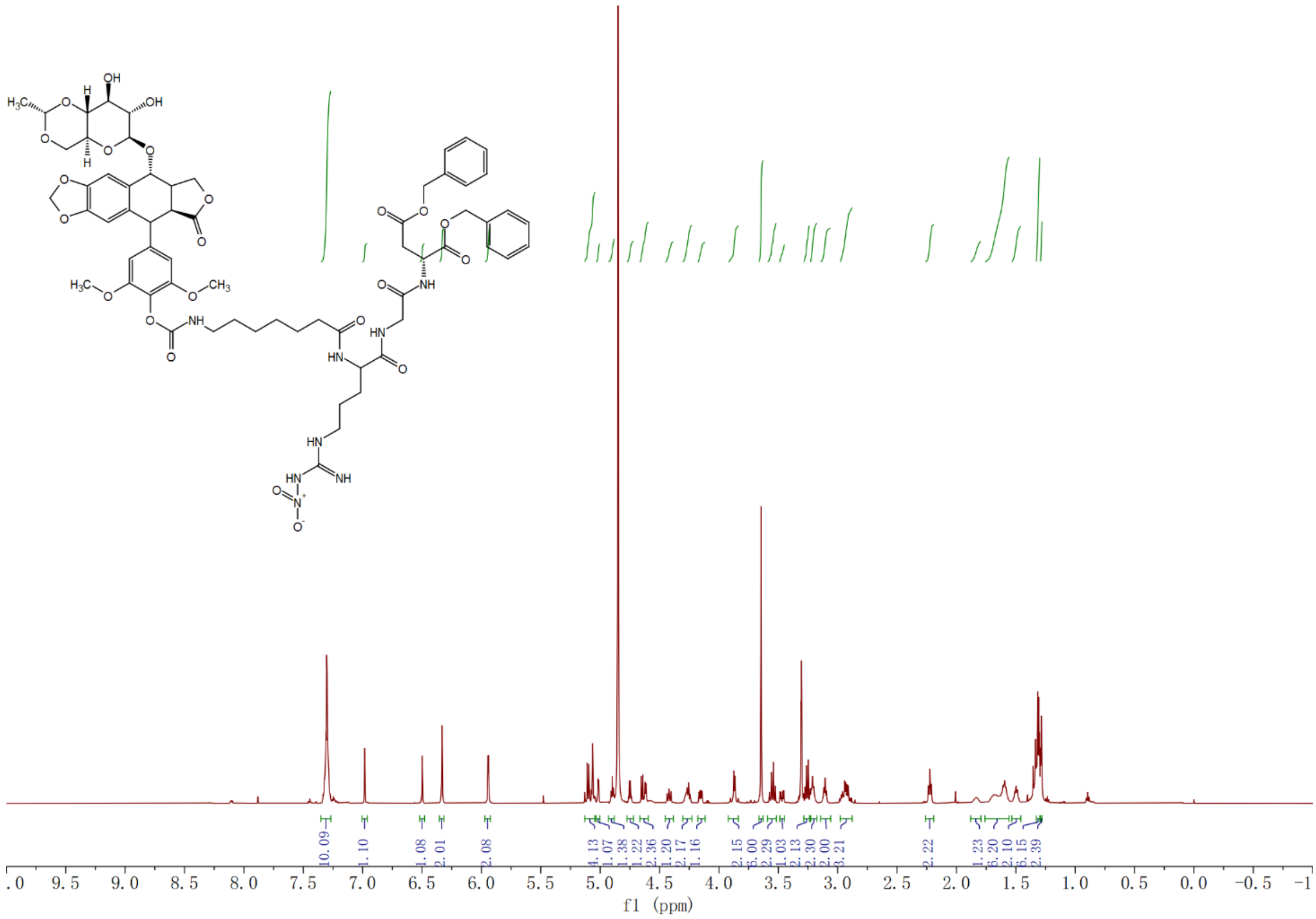

B

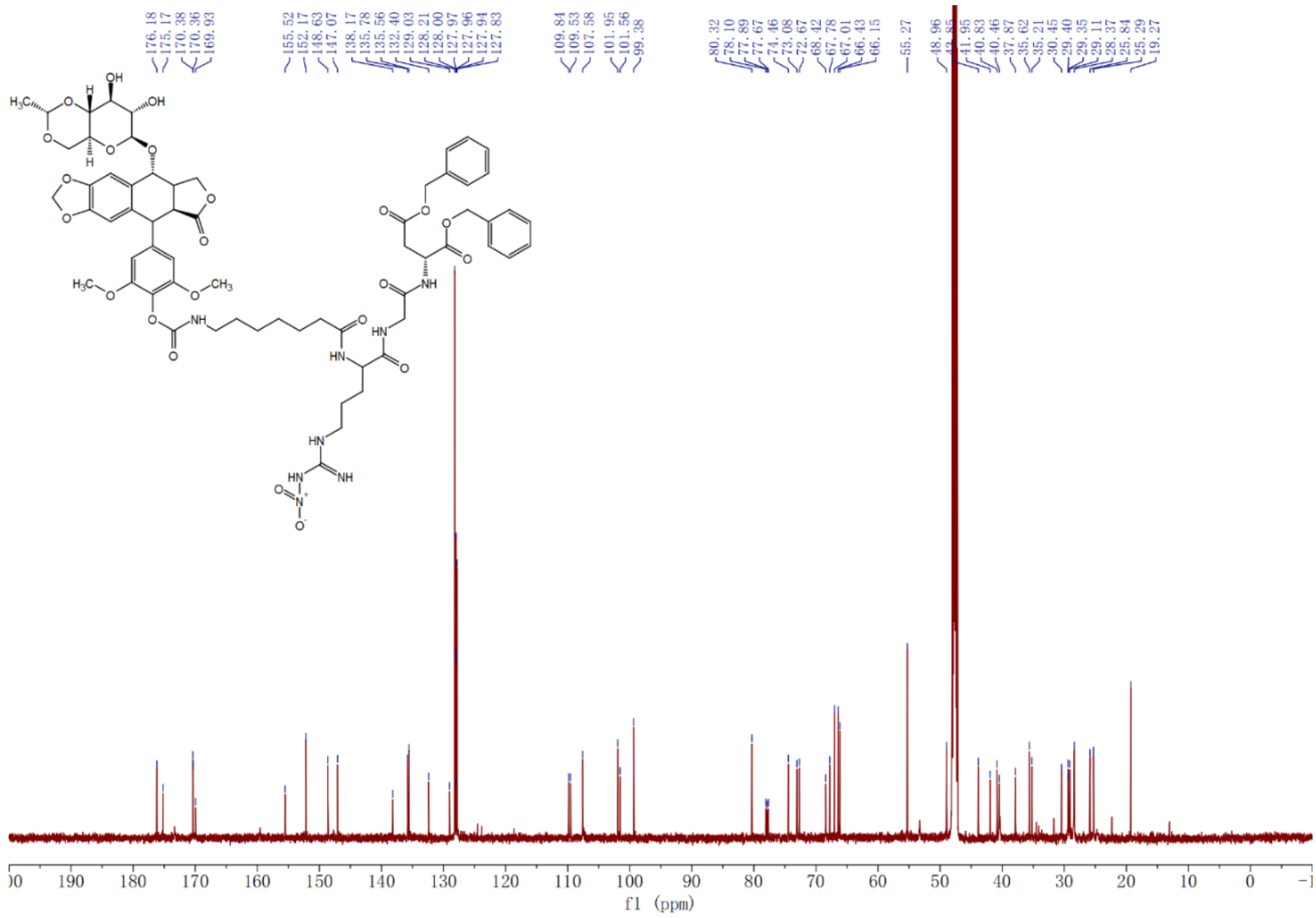

Fig. S13. ^{1}H-NMR (A) and ^{13}C-NMR (B) of Compound 7

A

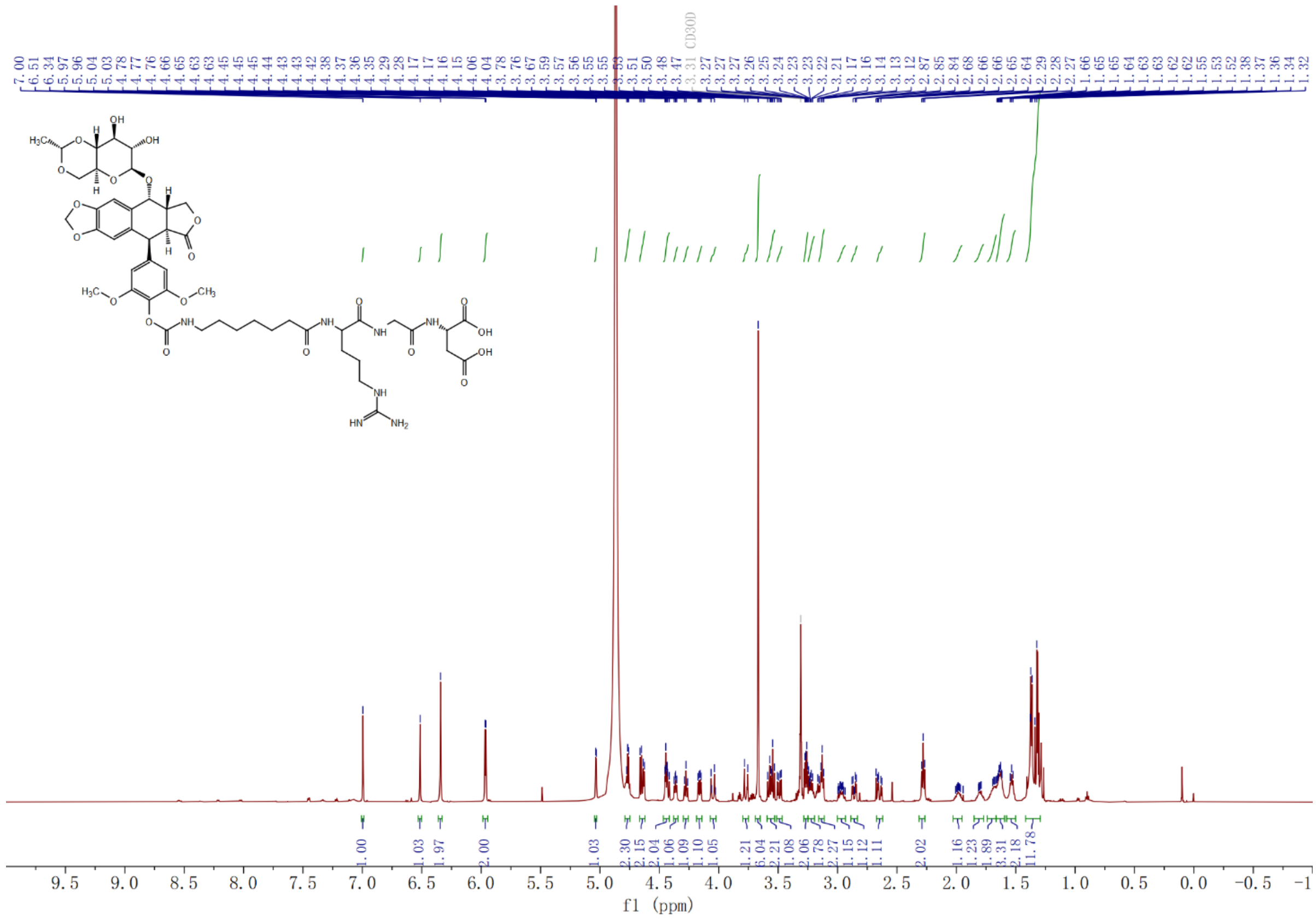

B

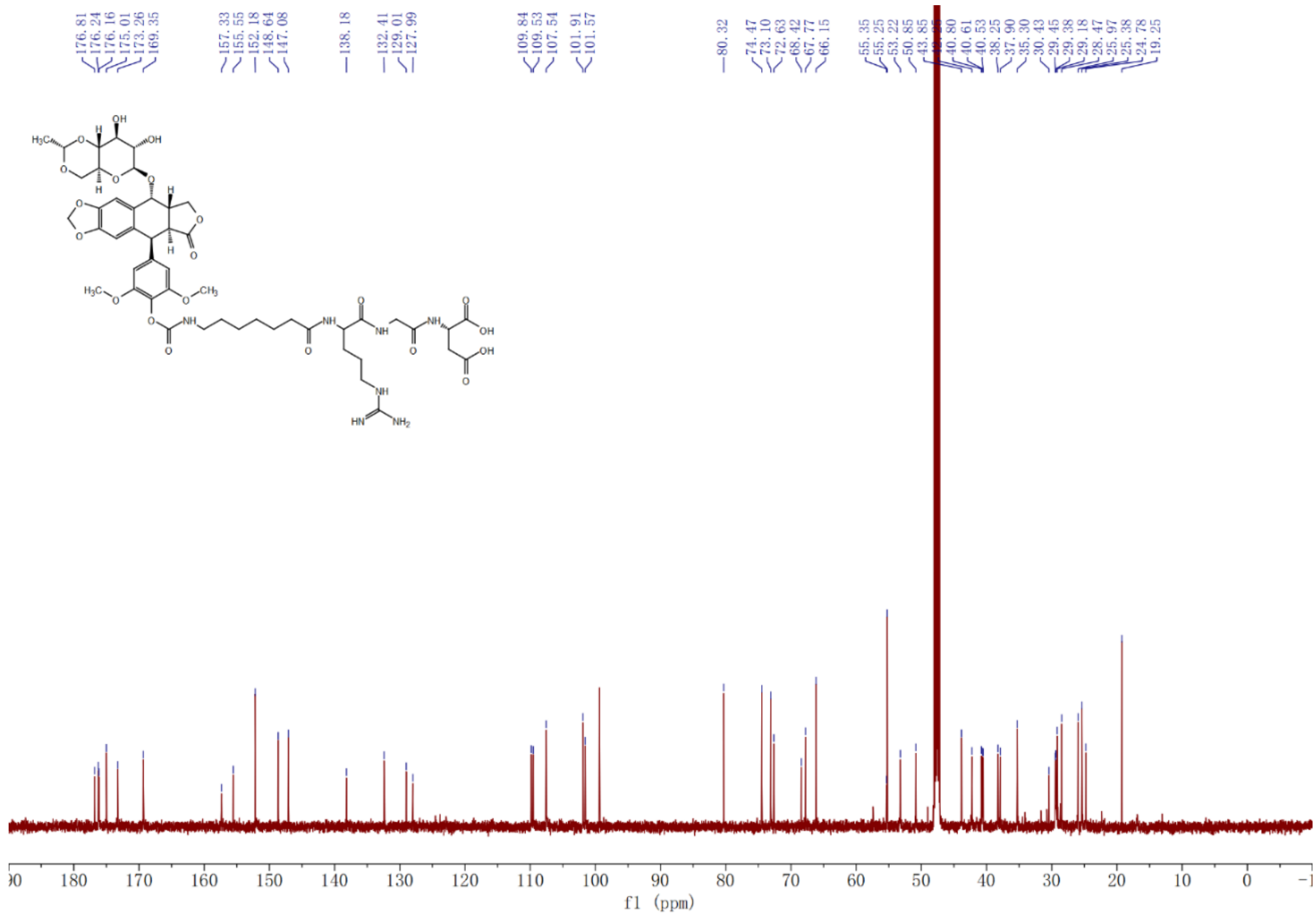

Fig. S14. ^{1}H-NMR (A) and ^{13}C-NMR (B) of ETP-CM-RGD

A

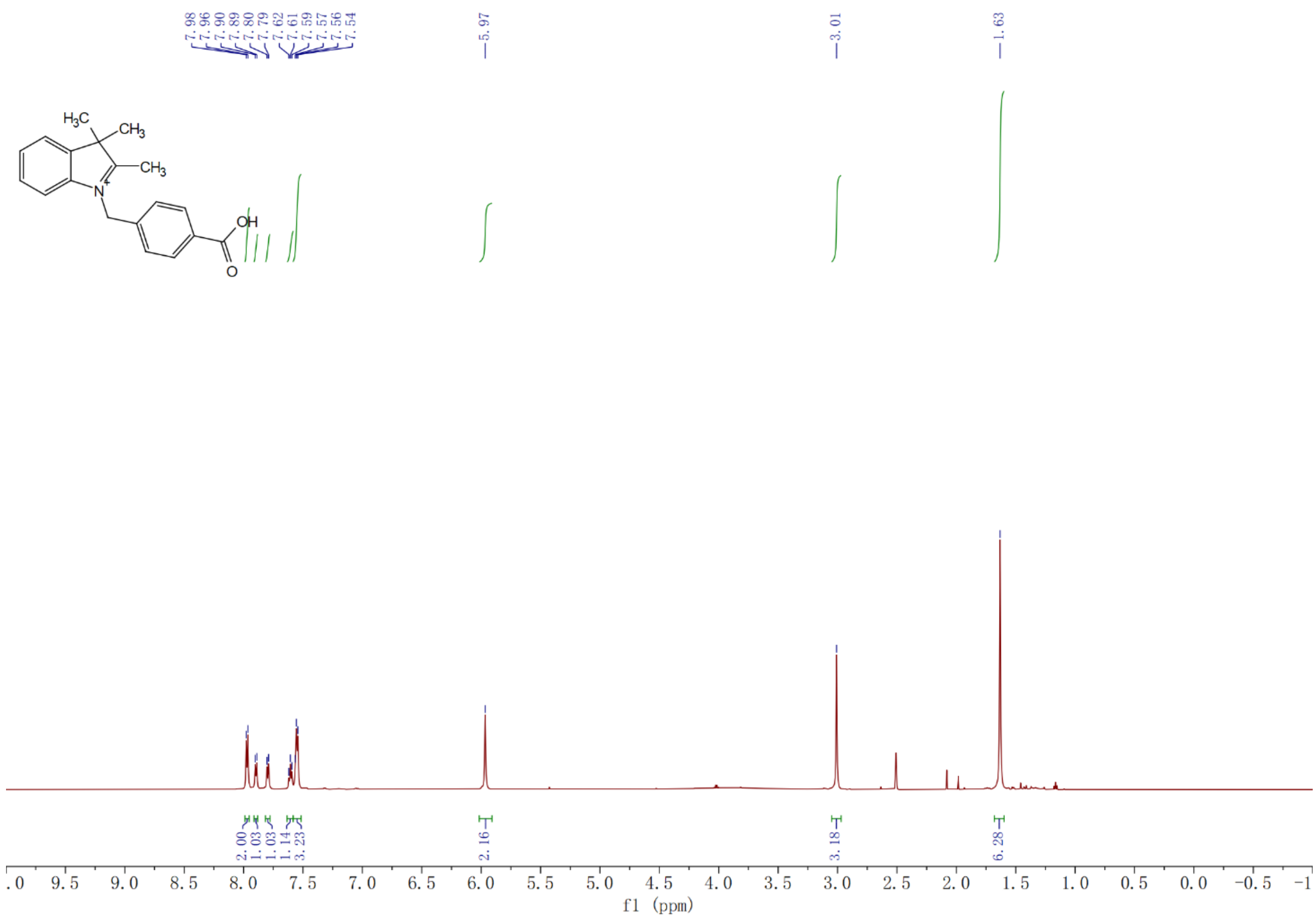

B

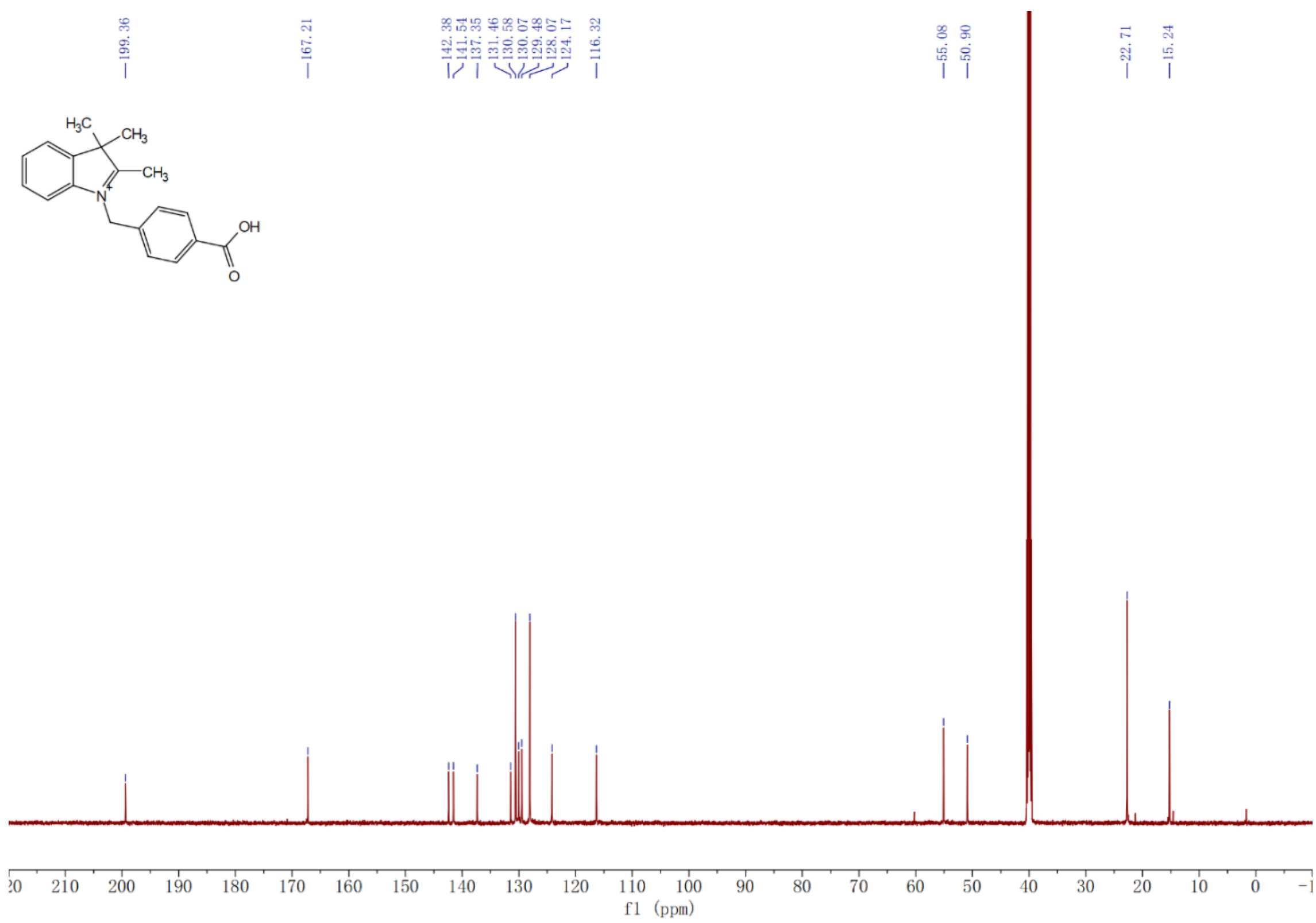

Fig. S15. ^{1}H-NMR (A) and ^{13}C-NMR (B) of Compound 8

A

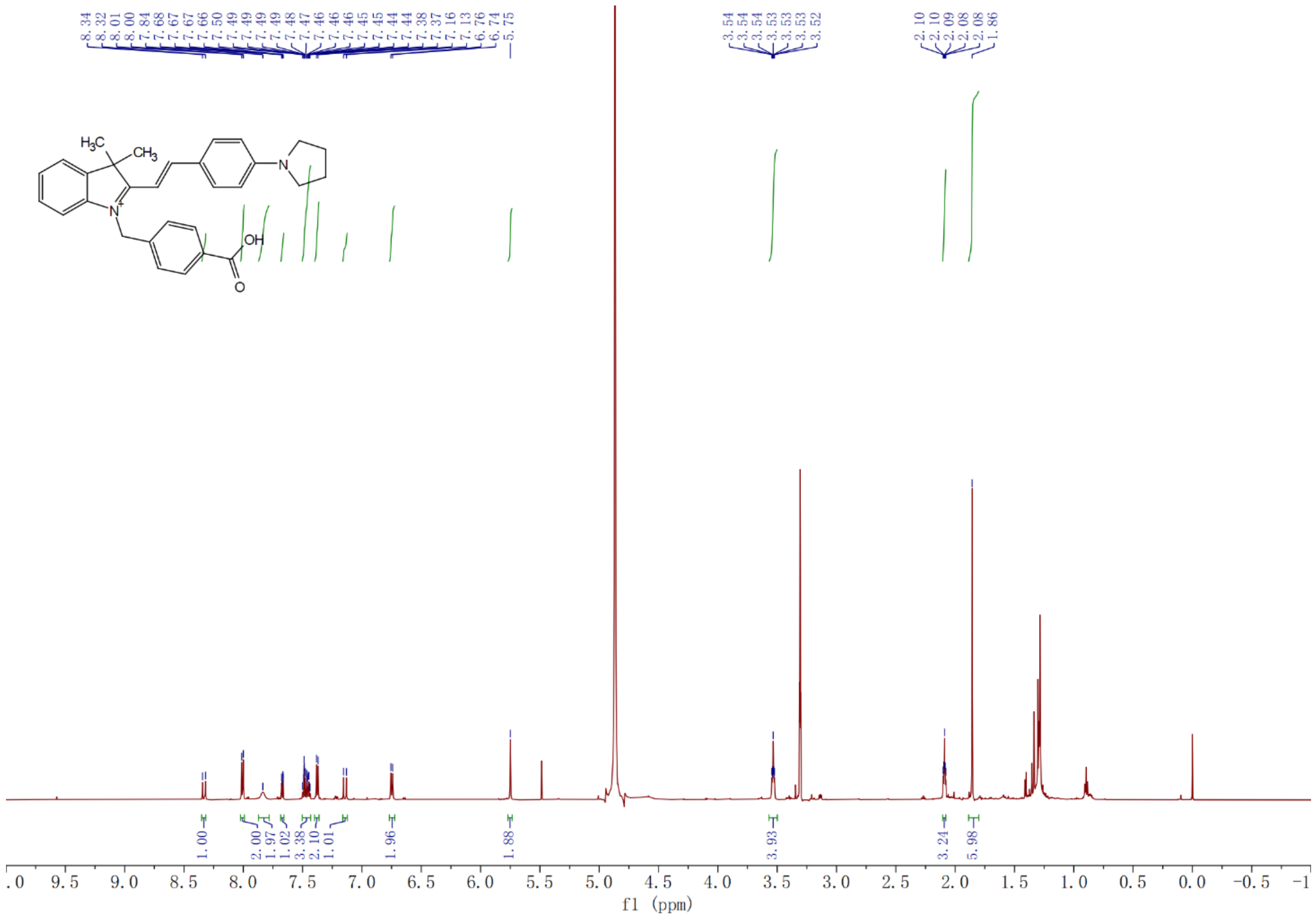

B

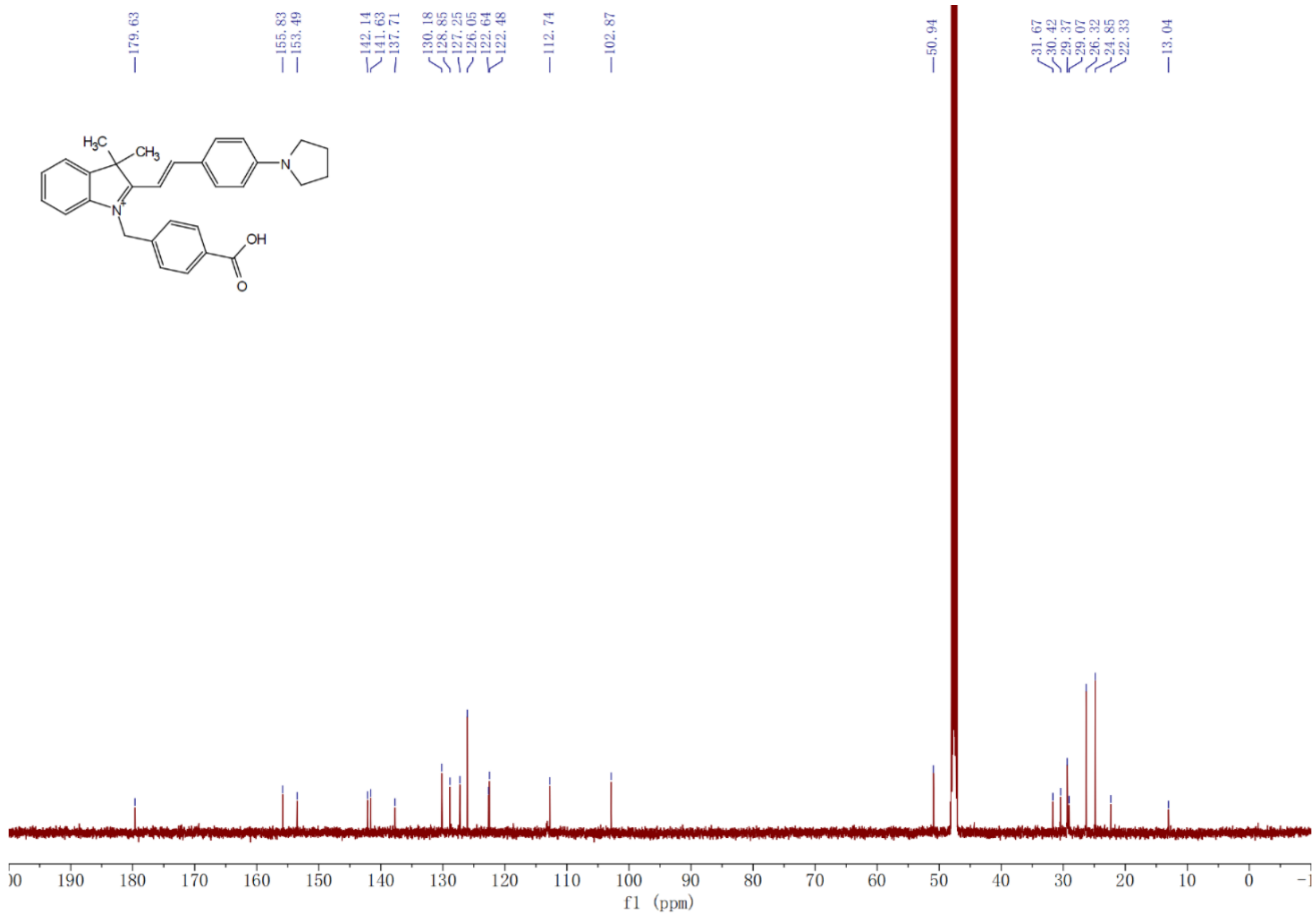

Fig. S16. ^{1}H-NMR (A) and ^{13}C-NMR (B) of Compound 9

A

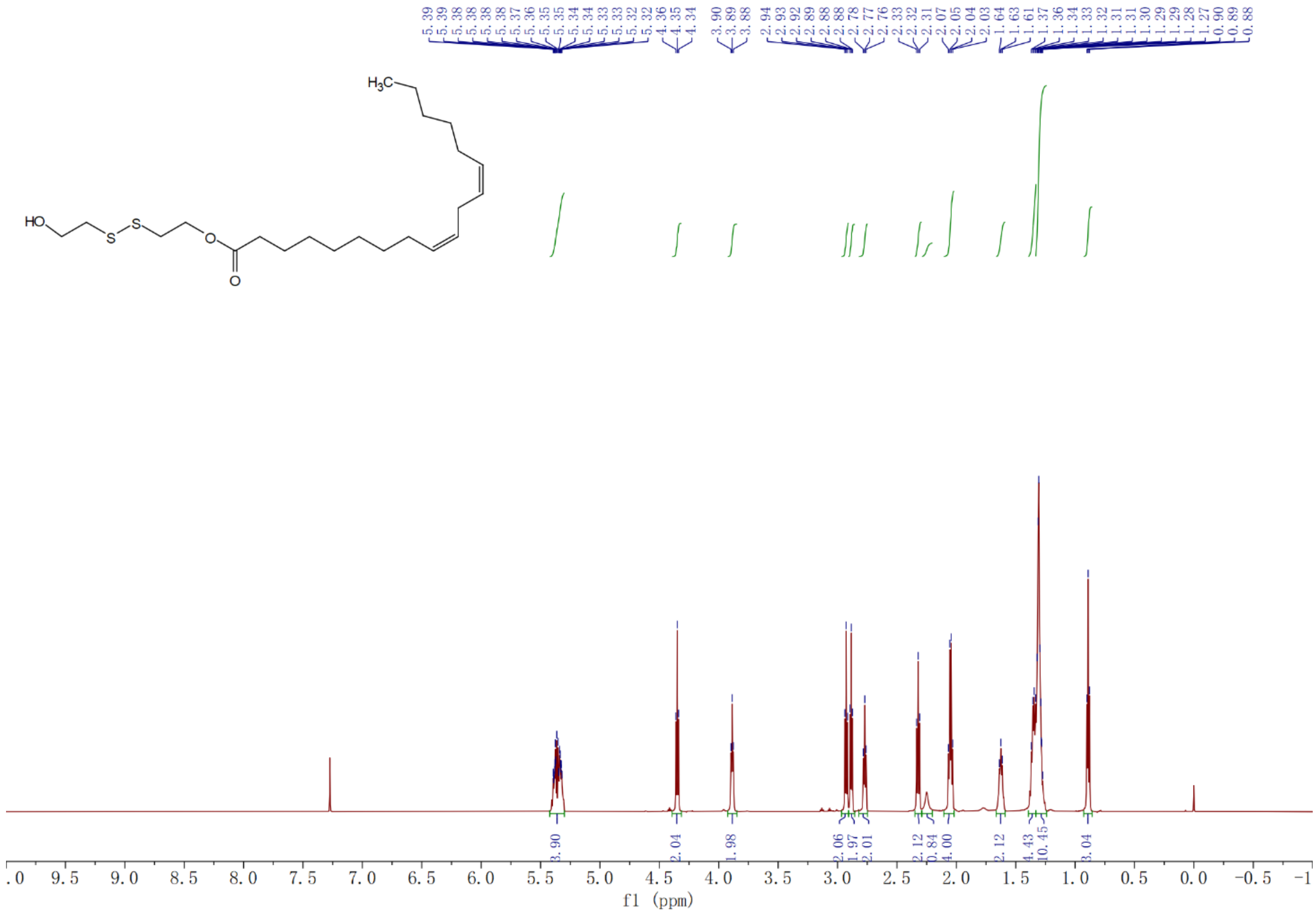

B

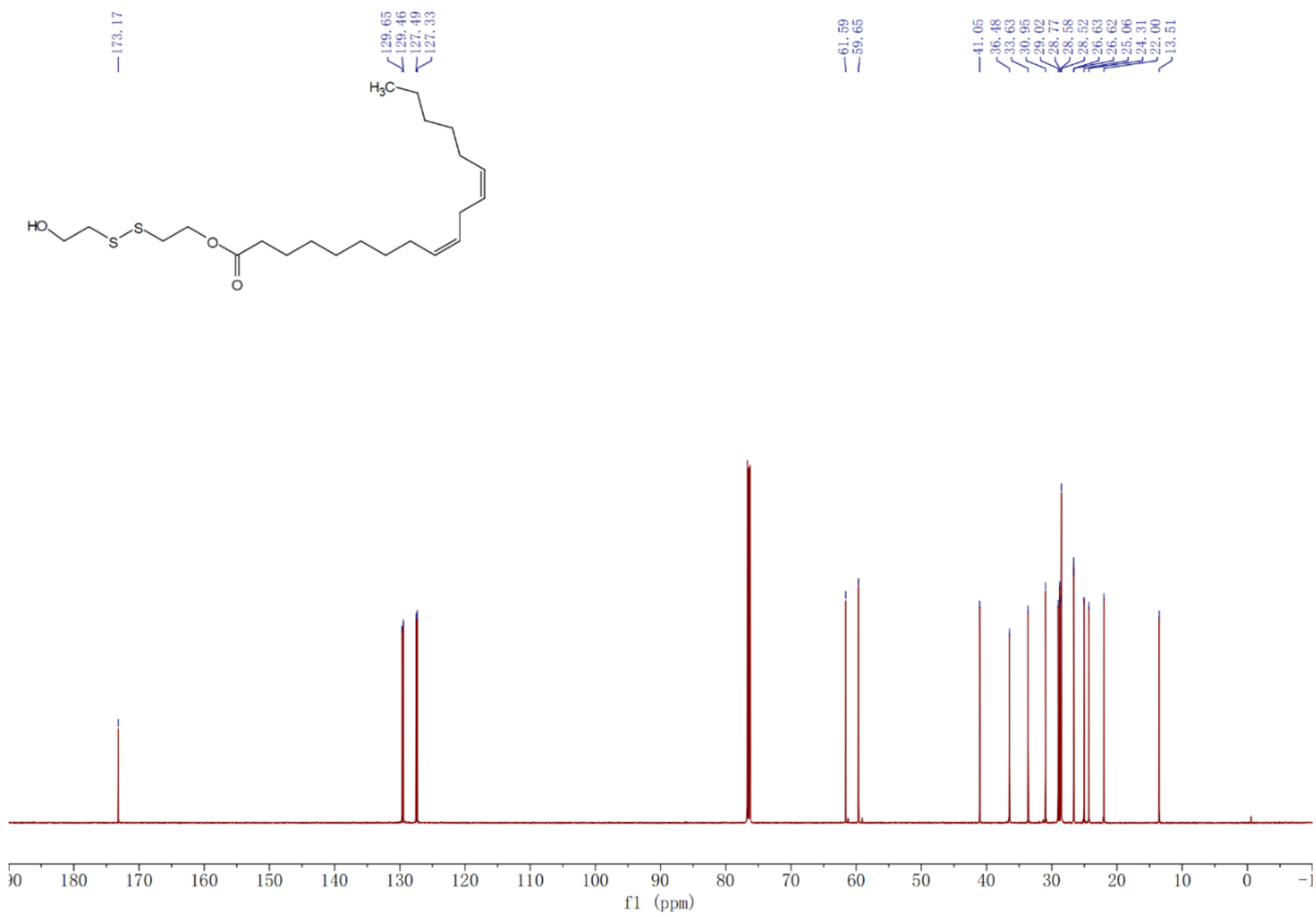

Fig. S17. ^{1}H-NMR (A) and ^{13}C-NMR (B) of Compound 10

A

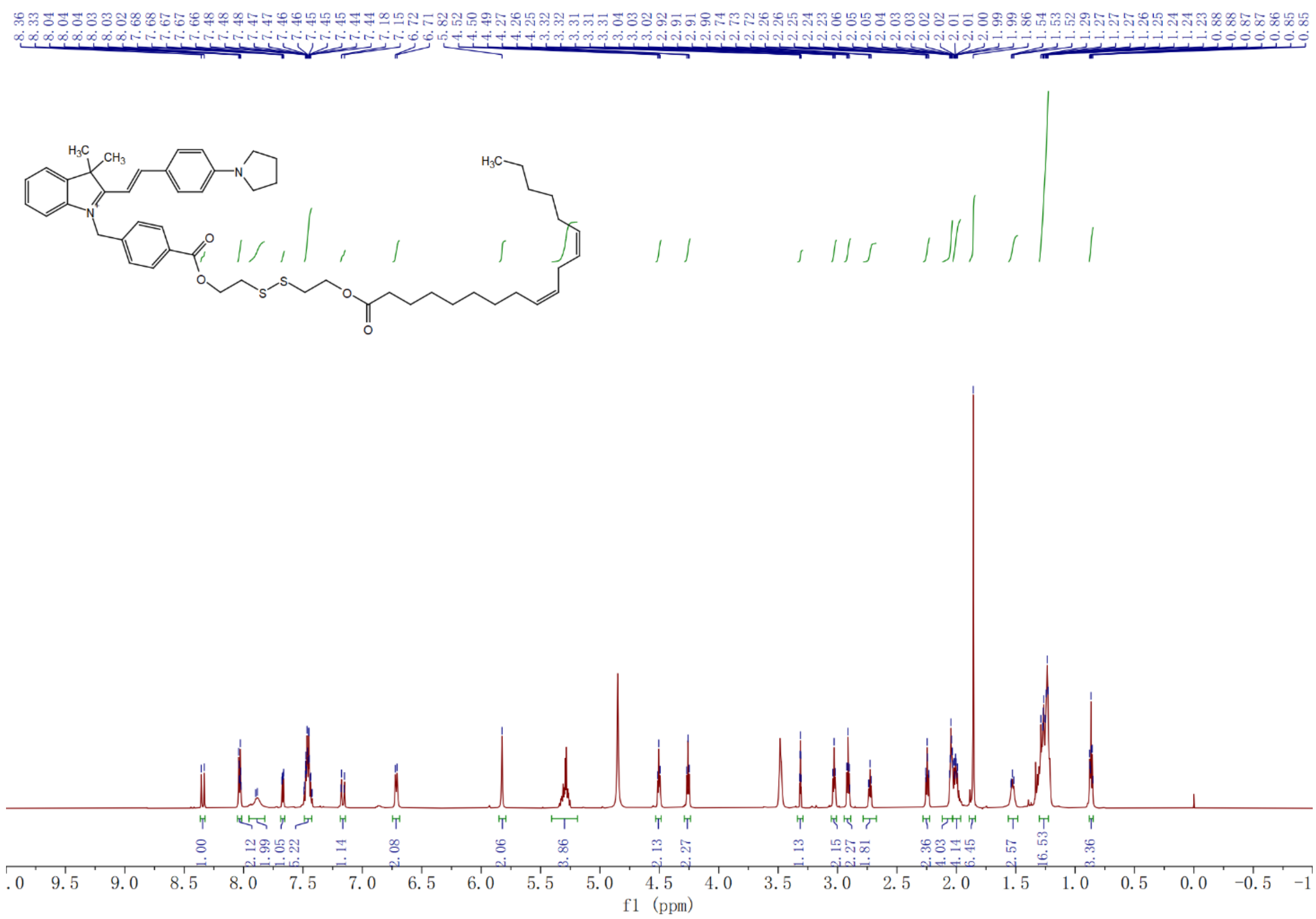

B

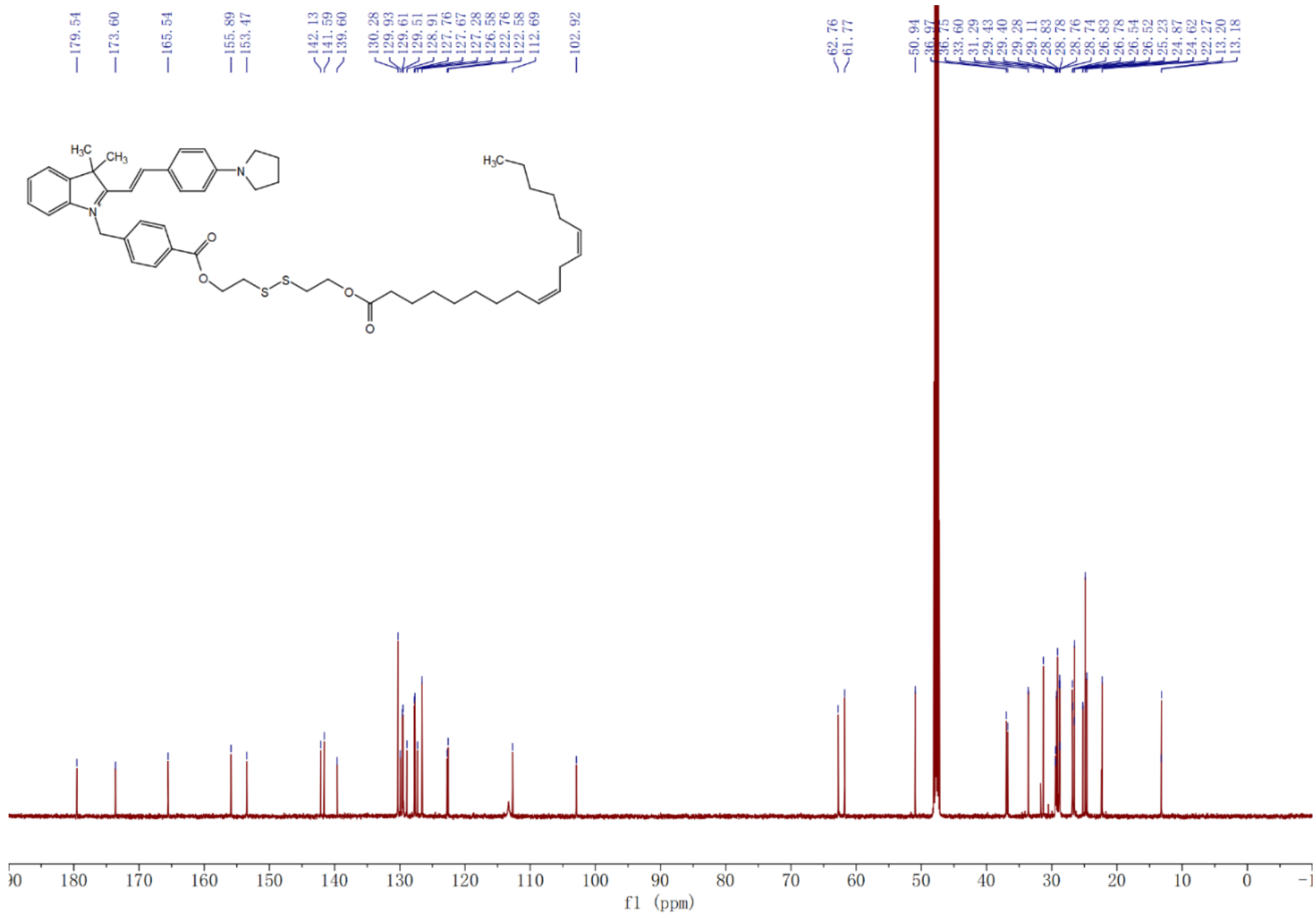

Fig. S18. ^{1}H-NMR (A) and ^{13}C-NMR (B) of IAA-SS-LA
